# Multigram-scale chemoenzymatic synthesis of diverse aminopolycarboxylic acids as potential metallo-β-lactamase inhibitors†

**DOI:** 10.1039/d3ob01405c

**Published:** 2023-12-12

**Authors:** Mohammad Faizan Bhat, Alejandro Prats Luján, Mohammad Saifuddin, Peter Fodran, Gerrit J. Poelarends

**Affiliations:** a Department of Chemical and Pharmaceutical Biology, Groningen Research Institute of Pharmacy, University of Groningen Antonius Deusinglaan 1 9713 AV Groningen The Netherlands g.j.poelarends@rug.nl +31-50-3633354 https://www.rug.nl/staff/g.j.poelarends/ https://twitter.com/gpoelarends

## Abstract

Toxin A, a precursor to naturally occurring aspergillomarasmine A, aspergillomarasmine B, lycomarasmine and related aminopolycarboxylic acids, was synthesized as the desired (2*S*,2′*S*)-diastereomer on a multigram-scale (>99% conversion, 82% isolated yield, dr > 95 : 5) from commercially available starting materials using the enzyme ethylenediamine-*N*,*N*′-disuccinic acid lyase. A single-step protection route of this chiral synthon was developed to aid *N*-sulfonylation/-alkylation and reductive amination at the terminal primary amine for easy derivatization, followed by global deprotection to give the corresponding toxin A derivatives, including lycomarasmine, in moderate to good yields (23–66%) and with high stereopurity (dr > 95 : 5). Furthermore, a chemoenzymatic route was developed to introduce a click handle on toxin A (yield 72%, dr > 95 : 5) and its cyclized congener for further analogue design. Finally, a chemoenzymatic route towards the synthesis of photocaged aspergillomarasmine B (yield 8%, dr > 95 : 5) was established, prompting further steps into smart prodrug design and precision delivery. These new synthetic methodologies have the prospective of facilitating research into the finding of more selective and potent metallo-β-lactamase (MBL) inhibitors, which are urgently needed to combat MBL-based infections.

## Abstract 2

Chemoenzymatic syntheses of various aminopolycarboxylic acids, including click-handle-modified and photocaged derivatives, are presented. These strategies may facilitate development of effective metallo-β-lactamase inhibitors to fight infections.

## Graphical abstract

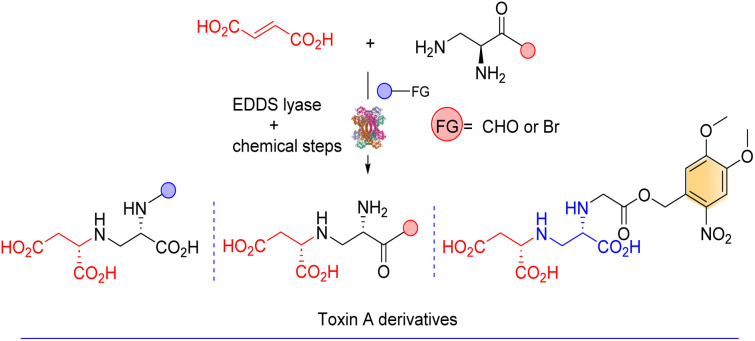

Antibacterial resistance remains one of the biggest public health challenges, especially because of the rate and magnitude at which it is acquired. While β-lactam containing drugs continue to be the most effective and widely used class of antibiotics,^1^ exhaustive and improper use has led to the rapid development of resistance among microorganisms.^2^ The mechanism of resistance mainly involves two enzyme classes, serine-β-lactamases (SBLs) and metallo-β-lactamases (MBLs), that cleave the β-lactam ring of the antibiotics and render them ineffective.^3^ While combination therapies with co-drugs (clavulanic acid, sulbactam, and tazobactam) are available to fight SBL producing bacteria, there is no clinically approved treatment for the highly resistant MBL producing bacteria.^4^ In particular, since its discovery in 2008, bacteria expressing New Delhi metallo-β-lactamase-1 (NDM-1) have emerged as one of the most clinically relevant challenges.^5^

Promisingly, the aminopolycarboxylic acid aspergillomarasmine A (AMA, Fig. 1) was discovered from a screen of 500 fungal natural products and identified as a selective and potent inhibitor of NDM-1 with an IC_50_ in the low-micromolar range.^6^ Interest in AMA as a scaffold for further development of drug candidates led to several total synthesis reports for AMA and analogues, based on a late-stage oxidation strategy (14 steps, 4% yield),^7^ an approach employing *o*-nosyl aziridine as a key intermediate (9 steps, 1% yield),^8^ a sulfamidate approach (6 steps, 19% yield),^9^ a Mitsunobu approach (6 steps, 28% yield),^10^ and an attractive biocatalytic strategy using AMA synthase (Fig. 1).^11^ Interestingly, biocatalytic approaches gain momentum in the synthesis of natural products.^12–15^ We have previously developed a chemoenzymatic route to AMA (Fig. 1), aspergillomarasmine B (AMB), toxin A (the natural precursor to AMA and AMB) and analogous compounds.^16^ This synthetic route benefits from a highly regio- and enantioselective carbon–nitrogen bond-forming step catalyzed by ethylenediamine-*N*,*N*′-disuccinic acid lyase (EDDS lyase). However, several limitations such as difficult purification and over-alkylation, resulting in relatively low product yields, inspired us to explore alternative routes towards the synthesis of diverse toxin A derivatives.

**Fig. 1:**
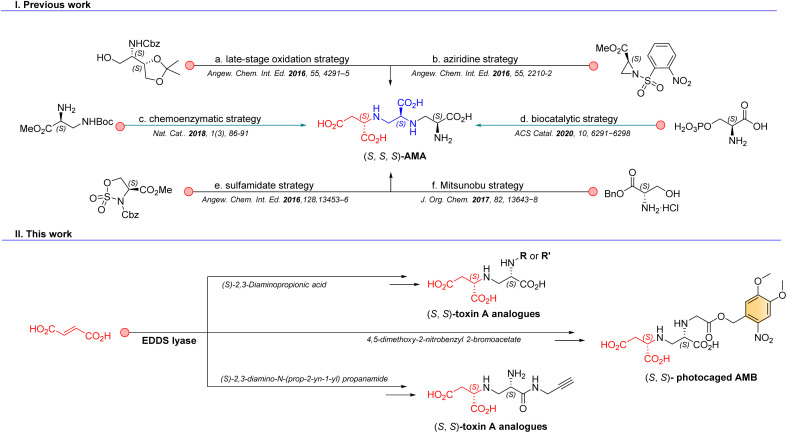
Synthesis of complex aminopolycarboxylic acids. (I) Previous chemical, chemoenzymatic and biocatalytic synthesis strategies towards the aminopolycarboxylic acid AMA and (II) our chemoenzymatic synthesis strategies towards related aminopolycarboxylic acids (*i.e.*, toxin A derivatives).

Along these lines, we envisaged that protection of the carboxylic groups of toxin A (compound 2 in Scheme 1) would lend the primary amine free for the synthesis of broad range of aminopolycarboxylic acids. Furthermore, to facilitate further derivatization of the toxin A pharmacophore, a new retrosynthetically designed chemoenzymatic route with inclusion of a propargylamine click handle was also anticipated. In contrast to our previous work,^16,17^ where an excess of chiral amine 1 (Scheme 1) was used to push the equilibrium of the enzymatic reaction, we decided to use an excess of fumarate in our new method to achieve multigram-scale synthesis of toxin A. We reasoned that the use of an excess of the chiral amine is not economically feasible, and furthermore it demands the use of a time and resource consuming purification procedure, which is based on a two-steps ion-exchange chromatography protocol, that eventually results in a significant loss of product yield.

**Scheme 1:**
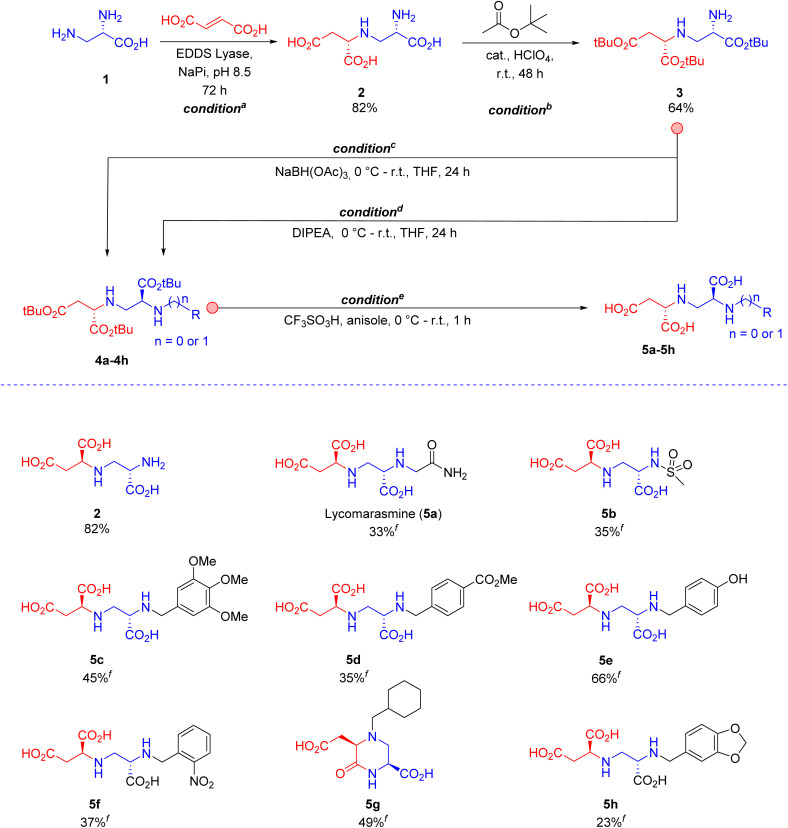
(Chemo)enzymatic synthesis of toxin A (2) and derivatives 5a–5h. Conditions and reagents: ^*a*^ Diamine substrate (1, 5.0 g, 35.6 mmol), fumaric acid (20.6 g, 177.9 mmol) and purified EDDS lyase (0.005 mol% based on diamine substrate 1) in buffer (200 mL, 50 mM Na_2_HPO_4_, pH 8.5), at room temperature for 72 h. ^*b*^ 200 mL *t*BuOAc, 2 mL 70% HClO_4_, at room temperature for 48 h. ^*c*^ Corresponding aromatic aldehyde precursors for products 5c–5h, NaBH(OAc)_3_ (2 equiv.), in THF, 0 °C to room temperature for 24 h. ^*d*^ Corresponding bromo or sulfonyl derivatives for products 5a and 5b, respectively; DIPEA (2 equiv.), in THF, 0 °C to room temperature for 24 h. ^*e*^ Trifluoromethanesulfonic acid (5 equiv.), anisole (5 equiv.) in DCM, 0 °C to room temperature for 1 h. A slightly different protocol for workup was used for the synthesis of 5g (ESI†). ^*f*^ Isolated yield of final products 5a–5h from intermediates 4a–4h after full deprotection and ion-exchange chromatography. The absolute configuration of toxin A (2) was assigned by ^1^H NMR spectroscopy using an authentic standard with known (2*S*,2′*S*) configuration.^16^ With both stereogenic centres being derived from (2*S*,2′*S*)-2, products 5a–5h have the correct absolute configuration. The dr of 5a–h was determined by ^1^H NMR to be >95 : 5 (ESI Fig. S1–S26†), indicating that no noteworthy epimerization occurred during the derivatization of 2.

Accordingly, we started with 1 g of amine 1, a 4-fold molar excess of fumarate, and 0.1 mol% of EDDS lyase in 200 mL of aqueous buffer (pH = 8.5, NaPi). Under these reaction conditions, 92% conversion of amine 1 into toxin A (2) was observed after 48 h (analyzed by ^1^H NMR). Although these results were noteworthy, a two-step purification procedure, consisting of an anion-exchange step to remove the remaining amine and a cation-exchange step to remove the remaining fumarate, was still demanded. After optimization of the reaction conditions (*condition*^*a*^ in Scheme 1), we found that a higher concentration of the two substrates in 200 mL buffer (pH 8.5, NaPi), using a 5-fold molar excess of fumarate over amine 1 and a prolonged reaction time (72 h), allowed >99% conversion of the amine into toxin A even with a substantial reduction in biocatalyst loading (0.005 mol%). Gratifyingly, a single cation-exchange purification step afforded 7 g of toxin A (isolated yield of 82%) as the desired (2*S*,2′*S*)-diastereomer (diastereomeric ratio (dr) > 95 : 5 based on ^1^H NMR), with the (2*S*)-stereogenic centre being set by EDDS lyase and the (2′*S*)-stereogenic centre derived from the starting substrate 1 (Scheme 1).^16^ This method (see ESI† for details) was repeated several times to obtain a total of ∼100 g of toxin A (2, Scheme 1) for the follow-up chemistry.

Next, several methods were attempted for the protection of the carboxylic acid groups of compound 2, such as esterification (benzyl alcohol, methanol, ethanol) under acidic conditions (HCl, H_2_SO_4_, SOCl_2_, and *p*-TsOH), but unfortunately these conditions either gave a low yield or the cyclization into a previously reported inactive toxin A congener.^8^ In addition, a three-step protection–deprotection sequence (*cbz* protection of amine, *t*Bu protection of carboxylic acids, and *cbz* deprotection) also proved difficult. Finally, a catalytic amount of perchloric acid (*condition*^*b*^ in Scheme 1; see ESI† for details) and the use of *tert*-butyl acetate both as a solvent and *t*Bu protecting agent provided an adequate solution, leading to fully protected compound 3 in 64% yield, without the need to purify this intermediate for the next step.

To demonstrate the synthetic usefulness of intermediate 3, reductive amination (*condition*^*c*^, Scheme 1) and *N*-alkylation/*N*-sulfonylation (*condition*^*d*^) were employed for the efficient synthesis of intermediates 4a–4h (see ESI† for details) in moderate to good yield (24–85%). Finally, the previously published method by Koteva *et al*.^8^ was used to fully deprotect the isolated intermediates (*condition*^*e*^, ESI†) and give the corresponding substituted aminopolycarboxylic acid products 5a–5h (Scheme 1) in modest to good yield (23–66%) and with high dr values of >95 : 5.^8^ With both stereogenic centres being derived from (2*S*,2′*S*)-2, products 5a–5h have the correct absolute configuration. Notably, the natural product lycomarasmine 5a, that was previously inaccessible with our one-pot synthesis strategy,^16^ could now be synthesized with 13% overall yield.

Next, we reasoned that inclusion of a click-handle into the toxin A scaffold would promote further derivatization. Towards this end, we performed amidation of the starting (*S*)-2,3-diaminopropionic acid (1) with propargylamine, after a protection–deprotection sequence (Scheme 2) to give the click-handle installed precursor 8. To our delight, the synthesized intermediate 8 was readily accepted by EDDS lyase as a non-native substrate for the hydroamination reaction (conversion >99%, 72 h) with 0.1 mol% biocatalyst loading to yield the desired compound 9 in multigram amount (5.1 g, yield 72%, dr > 95 : 5). Although, this intermediate in itself is an important building block for click-chemistry, we went further and synthesized a *t*Bu protected open chain and methyl protected cyclic congener (10 and 11, respectively, Scheme 2). Hence, this method can be used in the future for further development of important cyclic and acyclic chiral synthons.

**Scheme 2:**
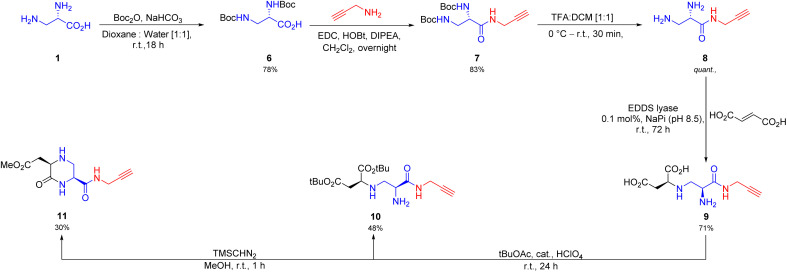
Chemoenzymatic synthetic route towards click-handle installed toxin A derivatives. The dr of 9 was determined to be >95 : 5 by ^1^H NMR and the absolute configuration was tentatively assigned as (*S*,*S*) based on analogy.

Finally, inspired by the recent advances in photocleavable prodrug design for precision therapy^18^ and safety,^18–21^ we embarked upon retrosynthetic analysis towards a photocleavable AMB (Scheme 3). We chose AMB for photocaging over AMA owing to its relatively easy synthesis but comparable inhibitory activity.^17^ Towards this end, *N*-alkylation of fully protected toxin A intermediate 3 with freshly synthesized photocaged-bromoacetic acid 12 (Scheme 3, see ESI† for details) was performed following our newly established synthetic method (*condition*^*d*^, see ESI† for details) to give 13 in 58% isolated yield. Finally, a standard deprotection procedure (*condition*^*e*^), gave the desired photocaged AMB product 14 (overall yield 8%; dr > 95 : 5 based on ^1^H NMR, with both stereogenic centres being derived from (2*S*,2′*S*)-2).

**Scheme 3:**
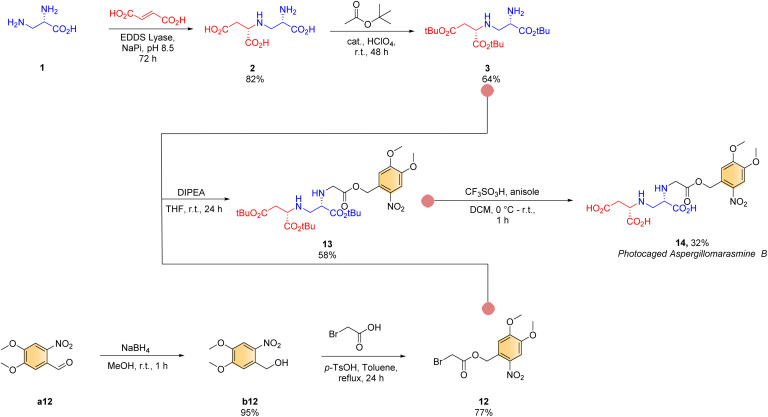
Chemoenzymatic route towards the synthesis of photocaged aspergillomarasmine B.

In conclusion, we have developed efficient chemoenzymatic routes for the facile and stereoselective synthesis of various complex aminopolycarboxylic acids at milligram- to gram-scale. We first optimized and scaled-up a biocatalytic methodology to prepare multigram amounts of (2*S*,2′*S*)-toxin A, with the (2*S*)-stereogenic centre being selectively installed by EDDS lyase and the (2′*S*)-stereogenic centre derived from the starting amine substrate. Subsequent chemical *N*-functionalization of this key chiral building block gives access to a series of toxin A derivatives with retention of configuration. Indeed, no significant epimerization has been observed during the *N*-functionalization procedures, providing the final products with high diastereomeric purity (dr > 95 : 5). We have also installed a click-handle into toxin A to further facilitate derivatization, enabling the preparation of large compound libraries for MBL inhibitor screening. Finally, inspired by recent developments in photocleavable prodrug design, we have used a retrosynthetic approach to chemoenzymatically prepare photocaged AMB. In future work, we aim to characterize this compound for its efficiency of uncaging and usefulness as MBL inhibitor in precision treatment of infections using model systems. Taken together, the new methodologies and compounds reported herein have the potential to facilitate research into the discovery of more potent and more selective MBL inhibitors, which are urgently needed to battle MBL-based infections.

## Experimental section

For detailed experimental procedures and characterization of compounds, see ESI.†

## Conflicts of interest

The authors declare no competing financial interests.

## Supplementary Material

OB-022-D3OB01405C-s001
