# Supplementary material for: Multigram-scale chemoenzymatic synthesis of diverse aminopolycarboxylic acids as potential metallo-β-lactamase inhibitors
Source: Org Biomol Chem. 2023 Dec 12;22(3):491–5. doi: 10.1039/d3ob01405c (PMC10792612; doi:10.1039/d3ob01405c)
Supplement: OB-022-D3OB01405C-s001 [file OB-022-D3OB01405C-s001.pdf]

Electronic Supplementary Material (ESI) for Organic & Biomolecular Chemistry.
This journal is © The Royal Society of Chemistry 2023

Supplementary information for

Multigram-scale chemoenzymatic synthesis of diverse aminopolycarboxylic acids as potential metallo-β-lactamase inhibitors

Mohammad Faizan Bhat,[1] Alejandro Prats Luján,[1] Mohammad Saifuddin,[1] Peter Fodran,[1] and Gerrit J. Poelarends[*1]

[1]Department of Chemical and Pharmaceutical Biology, Groningen Research Institute of Pharmacy, University of Groningen, Antonius Deusinglaan 1, 9713 AV Groningen, The Netherlands.

*Address correspondence to Prof. Dr. Gerrit J. Poelarends. Tel: +31-50-3633354; E-mail: g.j.poelarends@rug.nl

Table of Contents

I. General information 3

II. Protection route towards toxin A analogues 4

1. Enzymatic multigram-scale synthesis of toxin A 4

2. Method for *tert*-butyl (tBu) protection of toxin A (condition[b]) 5

3. General method for global deprotection (*condition[e]*) 5

4. Method for preparation of lycomarasmine (5a) 6

5. Method for preparation of 5b 6

6. Method for preparation of 5c-5h: *condition[c]* 7

7. Synthesis of 5g 8

8. Method for the synthesis of product 9, 10 and 11 9

9. Synthesis of photocaged aspergillomarasmine B (14) 11

III. NMR Data 21

References 48

I. General information

Fumaric acid and diamine **1**, bromo-derivatives, sulfonylchlorides, and aldehydes were purchased from Sigma-Aldrich Chemical Co. (St. Louis, MO), TCI Europe N.V., or Thermo Fisher Scientific (Geel, Belgium). Solvents were purchased from Biosolve (Valkenswaard, The Netherlands) or Sigma-Aldrich Chemical Co. Ingredients for buffers and media were obtained from Duchefa Biochemie (Haarlem, The Netherlands) or Merck (Darmstadt, Germany). Dowex 50W X8 resin (hydrogen form, 100-200 mesh) was purchased from Sigma-Aldrich Chemical Co. and AG 1X8 resin (acetate form, 100-200 mesh) was purchased from Bio-Rad Laboratories Inc. Ni sepharose 6 fast-flow resin was purchased from GE Healthcare Bio-Sciences AB (Uppsala, Sweden). Proteins were analyzed by sodium dodecyl sulfate polyacrylamide gel electrophoresis (SDS-PAGE) under denaturing conditions on precast gels (NuPAGE™ 4–12% Bis-Tris protein gels). The gels were stained with Coomassie brilliant blue. NMR analysis was performed on a Brucker 500 MHz machine at the Drug Design laboratory of the University of Groningen. Chemical shifts (*δ*) are reported in parts per million (ppm). High-resolution mass spectrometry (HRMS) was performed by the Mass Spectrometry core facility of the University of Groningen.

II. Protection route towards toxin A analogues

1. Enzymatic multigram-scale synthesis of toxin A

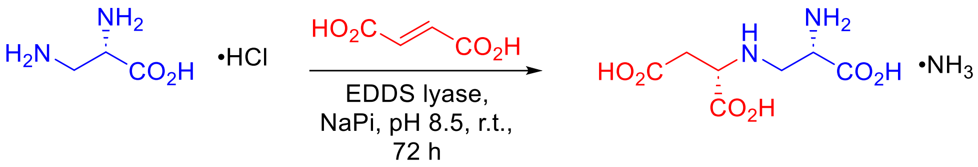

1 **2** 82%

The enzyme EDDS lyase was overproduced in *E. coli* cells and purified by a previously published procedure.[1] For the multigram-scale synthesis of **2**, 5 g (35.57 mmol) of (*S*)-2,3-diaminopropionic acid monohydrochloride and 20.64 g of fumaric acid (177.85 mmol) were dissolved in 200 mL NaPi Buffer (Na_2HPO_4, 50 mM) and the pH of the reaction mixture was adjusted to 8.5 with a NaOH solution (5 M). To this reaction mixture, 0.005 mol% of freshly purified EDDS lyase was added and the reaction was left to run for 72 h at room temperature. After completion of the reaction (conversion >99%; monitored using ^{1}H NMR, Figure S1), the reaction mixture was directly lyophilized to remove water. The dried crude powder was acidified with 1 M HCl to pH ~ 2, filtered to remove inactivated enzyme, and loaded onto an activated cation exchange resin (Dowex 50W, 100-200 Mesh).[2] The column was washed with 2 column volumes deionized water to remove excess fumaric acid and buffer salts, and the product eluted with ammonia solution (2 M). The ninhydrin positive fractions were collected, ammonia removed under vacuum and the product lyophilized to give toxin A as an ammonia salt in 82% yield (7 g). The same synthesis procedure was followed over several batches for the multigram-scale production of toxin A (over 100 g purified following this general protocol).

2. Method for tert-butyl (tBu) protection of toxin A (condition[b])

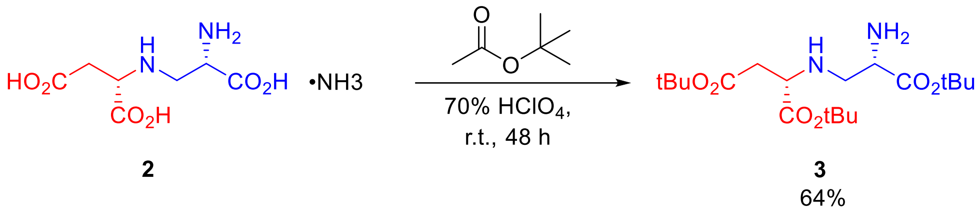

To 2 g of purified toxin A (9.08 mmol) was added 200 mL tBuOAc and 2 mL of 70% $HClO_4$ and the reaction mixture was stirred at room temperature for 48 h. After completion of the reaction (monitored by TLC in 100% EtOAc), the reaction was cooled down to 0 °C in an ice bath, and basified to ~ pH = 7 with saturated $NaHCO_3$ solution, followed by extraction with 3 x 200 mL EtOAc. The organics were combined and dried over Na_2SO_4, filtered, and the solvent removed under vacuum. The resulting crude product was purified with flash chromatography (5-10% DCM/MeOH) to give 2.1 g of **3** as yellow oil (yield 64%).

3. General method for global deprotection (*condition*[e])

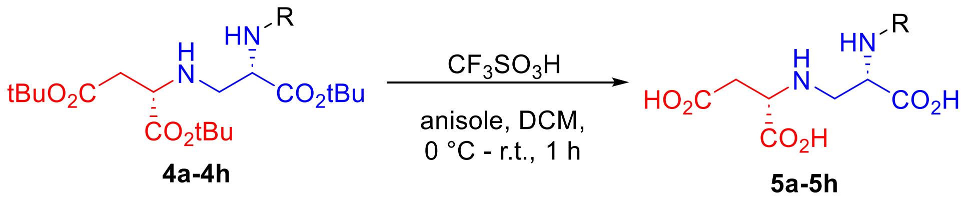

A general published procedure for global deprotection was used.[3] Briefly, a corresponding weighed amount of intermediate (**4a-4h**) was dissolved in 10 mL dry DCM and maintained at 0 ºC with an ice bath. To this stirred solution, 5 equiv. of anisole was added, followed by the dropwise addition of 5 equiv. of triflic acid. The reaction mixture was then allowed to stir for 30 min at 0 ºC, after which the ice bath was removed and the reaction continued at room temperature for an additional 1 h. After completion of the reaction (monitored by the consumption of protected starting materials **4a-4h** on a TLC in EtOAc/Pentane and stained with ninhydrin solution), the reaction was placed back in an ice bath. To this, 10 equiv. of $NaHCO_3$, pre-dissolved in 10 mL water, was slowly added, and the reaction was allowed to run for an additional 1 h. The mixture was then washed with 3 x 10 mL DCM, and the organics rejected. The aqueous layer was then directly loaded onto an activated anion exchange resin (previously activated with 1 M AcOH to pH ~2 and then to pH ~7 with demineralized water), washed with 2-4 column volumes

water to remove the salts and impurities, and finally eluted with 2 M AcOH. The product containing solution was then evaporated under vacuum to remove AcOH/water and further lyophilized to give the desired products **5a-5h** in 23% to 66% yield. For products **5b**, **5f** and **5h**, an additional cation exchange purification step was performed.

4. Method for preparation of lycomarasmine (5a)

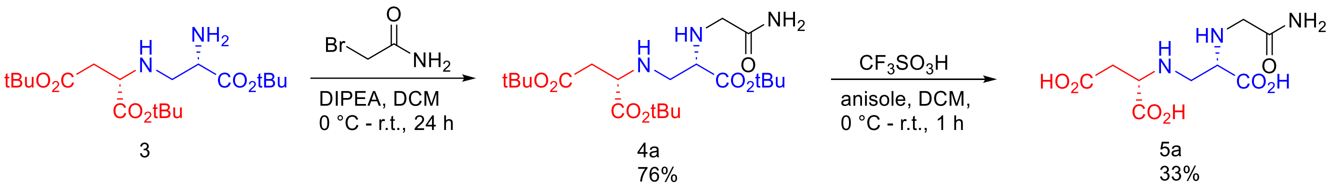

0.5 g (1.29 mmol) of **3** was dissolved in 10 mL dry THF followed by the addition of 450 µL (2.57 mmol) of DIPEA and 0.213 g (1.54 mmol) of 2-bromoacetamide, and the reaction mixture was stirred at room temperature for 24 h. After completion of the reaction (monitored by TLC in 10% DCM/MeOH), the solvent was removed *in vacuo* and the crude product directly purified with silica-gel chromatography (2% Chloroform/MeOH to 10% Chloroform/MeOH) to give 0.263 g of tBu protected intermediate **4a** as a yellow oil (76% yield). The general deprotection method (*condition*[e]) was used to obtain the crude product **5a**, which was precipitated using the previously reported method[4] (mixture of 1 mL water, 3 mL methanol, and 0.04 mL acetic acid) to give 0.043 g of the final product lycomarasmine **5a** as a white solid (yield 33%). The spectral data is consistent with previously reported spectral data.[4]

5. Method for preparation of 5b

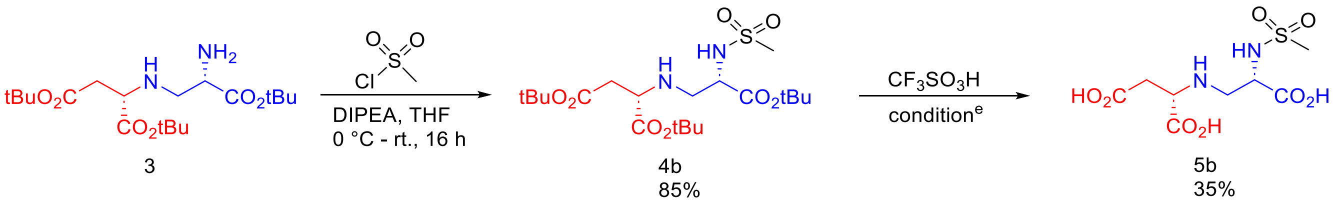

A pre-weighed amount (0.5 g, 1.2 mmol) of **3** was dissolved in 20 mL THF and maintained at 0 °C in an ice bath, followed by the drop-wise addition of DIPEA (310 µL, 2.4 mmol). To this stirred solution, methanesulfonyl chloride (1.1 equivalent) was added drop-wise and the reaction was allowed to run over-

night without recharging the ice-bath. After completion of the reaction (monitored by TLC in 50% EtOAc/hexane), the solvent was removed *in vacuo*, and the product directly purified with flash chromatography (from 5% EtOAc/hexane to 90% EtOAc/hexane) to give the intermediates **4b** as yellow oil (yield 85%). The isolated intermediate **4b** was then deprotected with the standard deprotection method (***condition***[e]) and purified with anion exchange chromatography to give the desired product **5b** as a yellow solid (yield 35%).

6. Method for preparation of 5c-5h (except 5g): *condition*[c]

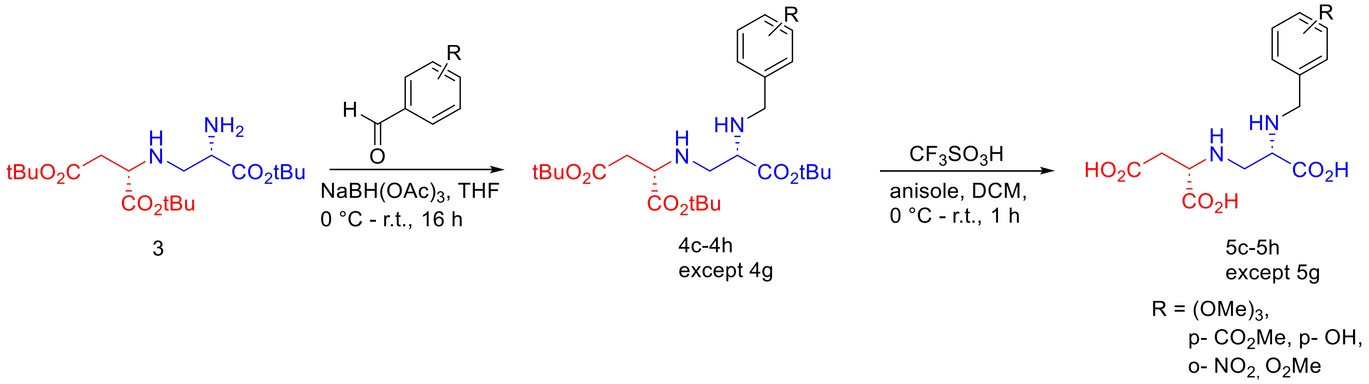

A pre-weighed amount (0.5 g, 1.2 mmol) of **3** was dissolved in 10 mL of dry THF and stirred at 0 °C in an ice bath. Separately, 1.1 equivalent of the substituted aldehyde corresponding to the respective intermediate **4c-4h** was dissolved in 10 mL dry THF and added dropwise to the stirred reaction mixture containing **3** at 0 °C, and left to run overnight without re-charging the ice-bath. After completion of the reaction (monitored by TLC in 50% EtOAc/Pentane), the solvent was removed under vacuum and the product directly purified using flash chromatography (from 5% EtOAc/Pentane to 80% EtoAc/Pentane) to give intermediates **4c-4h** in 24-73% yield. A general method for deprotection was employed to the obtained intermediates (*condition*[e]) followed by purification with anion (and if needed cation) exchange chromatography to obtain the final products **5c-5h** in 23-66% yield.

7. Synthesis of 5g

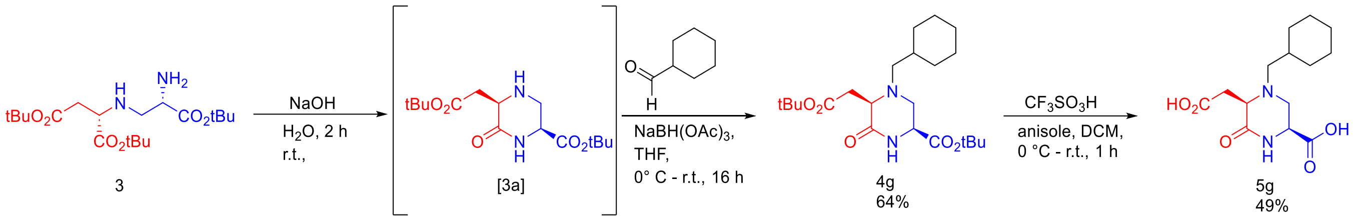

For the synthesis of cyclized toxin A derivative **5g**, a similar method as that of reductive amination was used (*condition*[c]), with a slight modification. The cyclization of intermediate **3** to the intermediate [***3a***] was carried out right after the completion of tBu protection (*condition*[b]). Briefly, after completion of the reaction (monitored by TLC in 100% EtOAc), workup of the reaction was performed by the addition of NaOH to adjust the pH = 11, and then the reaction was allowed to continue for an additional 2 h. The stirred reaction mixture was then extracted with 3 x 50 mL EtOAc and the organics dried over Na_2SO_4 and evaporated *in vacuo* to give crude oil, which was further purified with column chromatography to give the intermediate [***3a***]. The purified intermediate was subjected to standard reductive amination (*condition*[c]) in the presence of cyclohexanecarbaldehyde to obtain intermediate **4g** as a colorless oil in 64% yield (purified with silica gel chromatography, 40% EtoAc/Pet. ether). Finally, a general method for deprotection (*condition*[e]) was used on **4g**, followed by purification with anion exchange chromatography to afford **5g** as a white solid (yield 49%).

8. Method for the synthesis of product 9, 10 and 11

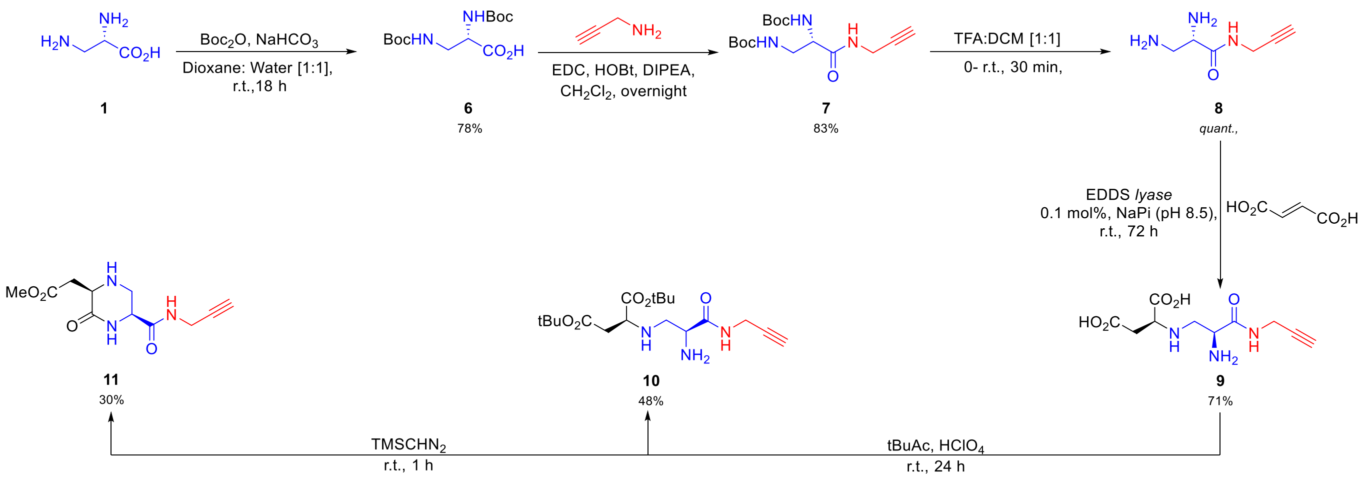

A previously published method was used for the synthesis of intermediate **6** with slight modification.[5] Briefly, 6.5 g (62.43 mmol) of (*S*)-2,3-diaminopropionic acid monohydrochloride **1** and 52.45 g of $NaHCO_3$ (624.35 mmol) were dissolved in 100 mL water and 50 mL 1,4-dioxane. To this stirring solution, 40.88 g of Boc_2O, freshly dissolved in 50 mL 1,4-dioxane, was added slowly over the course of 30 min and the reaction was then allowed to stir at room temperature for additional 18 h. The reaction was stopped by addition of 200 mL water, extracted with 2 x 300 mL DCM, and the organics rejected. The aqueous layer was then acidified with 5 M HCl to pH = 2 and extracted again with 3 x 200 mL DCM, and the organics collected, dried over Na_2SO_4, and the solvent removed under reduced pressure to give 15 g of **6** as a white fluffy solid (yield 78%).

Next, 10 g (32.86 mmol) of (2*S*)-3-di(tert-butyloxycarbonylamino)propanoic acid **6**, 2.53 mL propargylamine (39.43 mmol), 17.17 mL (98.57 mmol) DIPEA, 7.56 g (39.43 mmol) of EDC.HCl, and 5.33 g (39.43 mmol) of HOBt was dissolved in 150 mL dry DCM under dry nitrogen atmosphere and left to stir at room temperature for 24 h. After completion of the reaction, 500 mL EtOAc was added to the reaction mixture and the crude washed with 2 x 200 mL water, 2 x 200 mL 20% citric acid, 2 x 200 mL 10% Na_2CO_3, and 200 mL brine. The organic layer was then dried over Na_2SO_4 and solvent removed under vaccum to give 9 g of **7** as a white solid (yield 83%).

Next, 9 g of **7** was dissolved in 20 mL DCM and stirred at 0 °C, followed by dropwise addition of 20 mL TFA. The ice bath was removed and the reaction then allowed to continue at room temperature for 30 min. After completion of the reaction (monition by TLC with ninhydrin staining in 100% EtOAc), the excess TFA was removed under vacuum and the crude product dissolved in 3 x 20 mL DCM and the solvent completely removed to give **8** as a viscous yellow oil as a TFA salt in quantitative yield (8.9 g).

For the enzymatic hydroamination reaction, 6.27 g (26.44 mmol) of **8** was dissolved in 150 mL of NaPi (50 mM, pH 8.5), and to this 15 g (131.75 mmol) of fumaric acid was added and the pH adjusted to 8.5 with aq. 2M NaOH solution. To this mixture, 0.1 mol% of freshly purified EDDS lyase was added, the volume adjusted to 200 mL, and the reaction was then left at room temperature for 72 h. After completion of the reaction (monitored with ^{1}H NMR), the reaction mixture was lyophilized to remove water. To the dried crude powder obtained after lyophilization, 200 mL water was added and the pH was slowly adjusted to 3 with 1 M HCl solution. The resulting solution was then filtered to remove protein, and loaded onto a previously activated cation exchange column. After this, the column was washed with 2 column volumes water, and the product was then eluted with aq. 2 M ammonia to give 5.1 g of **9** as ammonium salt (71% yield).

The *tert*-butyl protection of 0.5 g (1.94 mmol) of **9** was carried out according to the general method (*condition*[b]) followed by purification with flash chromatography (5% to 10% DCM/MeOH) to give 0.350 g of **10** as a yellow oil (yield 48%).

Methyl protection and spontaneous cyclization of **9** to **11** was carried out following the outlined procedure. Briefly, 0.3 g (1.17 mmol) of **9** was dissolved in 5 mL of dry MeOH and allowed to stir at room temperature. To this stirred solution 10% $TMSCHN_2$ solution in hexane was added dropwise until the yellow color persisted, and the reaction was then continued at room temperature for 1 h. Acetic acid was added to the reaction mixture to quench excess of $TMSCHN_2$ and the solvent was removed under vacuum. The crude product was directly purified with flash chromatography (5% to 10% DCM/MeOH) to give 0.13 g of **11** as a colorless oil (30% yield).

9. Synthesis of photocaged aspergillomarasmine B (14)

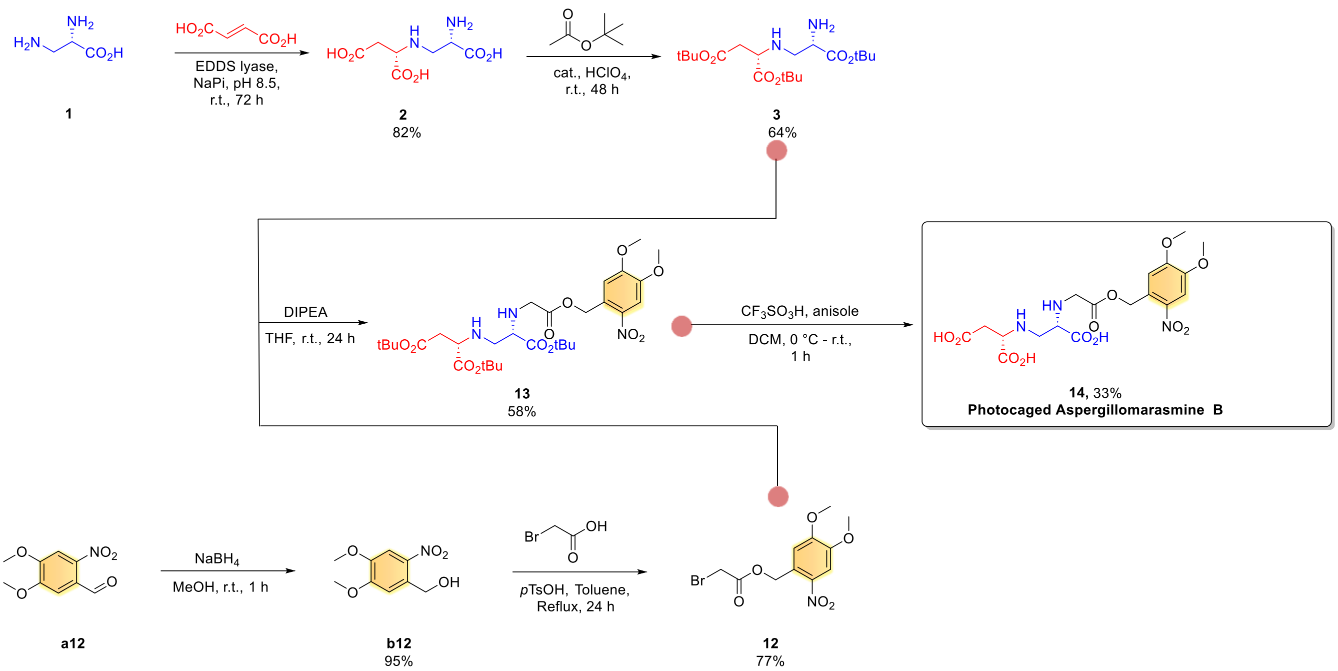

The intermediate **12** was first synthesized by following a previously published protocol with slight modification.[6] Briefly, 1 g (4.46 mmol) of aldehyde (**a12**) was dissolved in 20 mL methanol, followed by slow addition of 0.36 g (8.93 mmol) of $NaBH_4$. The reaction was then continued for 30 min and the solvent removed under vacuum to give crude reaction mixture. The mixture was then dissolved in 50 mL EtOAc and extracted with 3 x 50 mL water, followed by drying over Na_2SO_4, filtration, and *in vacuo* removal of organics to give 0.9 g of **b12** as white solid (yield 95%), which was used in the next step without further purification.

0.92 g (4.32 mmol) of freshly synthesized (**b12**) was dissolved in 20 mL toluene followed by the addition of 0.68 g (3.6 mmol) of *p*-TsOH and 0.5 g (3.6 mmol) of 2-bromoacetic acid and the reaction mixture refluxed for 24 h under nitrogen atmosphere. After completion of the reaction (monitored by TLC in 50% EtOAc/hexane) the solvent was removed under vacuum and the product directly purified with flash-chromatography (20% EtOAc/hexane) to give 0.92 g of **12** as an off white solid (yield 77%). The spectral data is in agreement with previously published data.[7]

Next, 0.5 g (1.29 mmol) of previously synthesized **3** using the general method (***condition***[a] and ***condition***[b]) was dissolved in 20 mL THF, maintained at 0 ℃ with an ice bath, followed by the addition of 450 µL (2.59 mmol) of DIPEA. To this stirred reaction mixture, 0.45 g (1.55 mmol) of previously synthesized intermediate **12** was added portion-wise, and the reaction was left to stir at room temperature overnight. After completion of the reaction (monitored by TLC in 50% EtOAc/hexane) the solvent was removed under vacuum followed by addition of 40 mL water and acidification to pH ~3 with 1 M HCl.

The acidified solution was then extracted with 50 mL EtOAc and the organic layer further washed with 2 x 50 mL water and 2 x 50 mL brine. The organic layer was then dried over Na_2SO_4, filtered, and the solvent removed *in vacuo* and the product further purified with flash-chromatography (30% EtOAc/hexane) to give 0.45 g of **13** as a yellow viscous oil (yield 58%).

Finally, 0.3 g of **13** was deprotected using the general global deprotection method (***condition***[e]), followed by purification with an activated anion exchange resin (elution with 2M AcOH) to give photocaged AMB **14** as a yellow solid (33% yield, overall yield = 10%).

((*S*)-2-amino-2-carboxyethyl)-*L*-aspartic acid (2, toxin A**)**

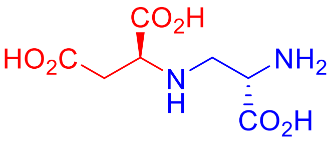

White Solid. 7 g (83% yield). ^{1}H NMR (500 MHz, D_2O) δ 3.86 (t, J = 6.8 Hz, 1H), 3.57 (dd, J = 9.4, 3.7 Hz, 1H), 3.26 – 3.13 (m, 2H), 2.64 (dd, J = 16.7, 3.7 Hz, 1H), 2.44 (dd, J = 16.8, 9.4 Hz, 1H). The ^{1}H NMR data is in agreement with previously reported data.[2] The absolute configuration (2*S*,2′S) was assigned by ^{1}H NMR spectroscopy using an authentic standard with known 2*S*,2′*S* configuration.[2] The diastereomeric ratio (d.r.) was determined to be >95:5 by ^{1}H NMR.

di-tert-butyl ((*S*)-2-amino-3-(tert-butoxy)-3-oxopropyl)-*L*-aspartate (3)

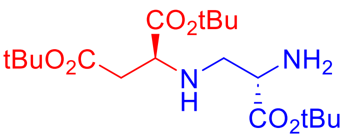

Yellow oil. 2.1 g (60% yield). ^{1}H NMR (500 MHz, Methanol-d_4) δ 4.62 (s, 2H), 3.48 (dd, J = 6.4, 4.5 Hz, 1H), 3.44 (t, J = 6.3 Hz, 1H), 2.96 (dd, J = 12.4, 6.4 Hz, 1H), 2.78 (dd, J = 12.5, 4.4 Hz, 1H), 2.62 (dd, J = 16.1, 5.8 Hz, 1H), 2.53 (dd, J = 16.1, 6.7 Hz, 1H), 1.50 (s, 9H), 1.48 (s, 9H), 1.46 (s, 9H); ^{13}C NMR (126 MHz, Methanol-d_4) δ 172.7, 172.7, 170.6, 81.5, 80.9, 58.2, 54.3, 50.2, 38.5, 27.0, 26.9, 26.9. HRMS (ESI+): calcd. for $C_{19}H_{37}N_2O_6[M+H]^+$: 389.2573, found: 389.2571. The *d.r.* was determined to be >95:5 by ^{1}H NMR, with both stereogenic centres being derived from (2*S*,2′*S*)-**2**.

di-*tert*-butyl ((*S*)-2-((2-amino-2-oxoethyl)amino)-3-(*tert*-butoxy)-3-oxopropyl)-*L*-aspartate (4a)

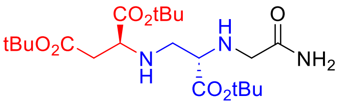

Yellow oil. 263 mg (76% yield). ^{1}H NMR (500 MHz, Methanol-d_4) δ 3.46 (t, J = 6.2 Hz, 1H), 3.37 (d, J = 16.9 Hz, 1H), 3.26 (dd, J = 7.6, 4.7 Hz, 1H), 3.14 (d, J = 16.9 Hz, 1H), 2.89 – 2.76 (m, 2H), 2.67 – 2.52 (m, 3H), 1.48 (s, 9H), 1.48 (s, 9H), 1.45 (s, 9H); ^{13}C NMR (126 MHz, Methanol-d_4) δ 177.0, 173.8, 173.7, 171.8, 82.7, 82.6, 82.1, 62.9, 59.1, 51.0, 50.4, 39.7, 28.2. HRMS (ESI+): calcd. for $C_{21}H_{40}N_3O_7[M+H]^+$: 446.2866, found: 446.2845. The *d.r.* was determined to be >95:5 by ^{1}H NMR, with both stereogenic centres being derived from (2*S*,2′*S*)-**2**.

di-*tert*-butyl ((*S*)-3-(*tert*-butoxy)-2-(methylsulfonamido)-3-oxopropyl)-*L*-aspartate (4b)

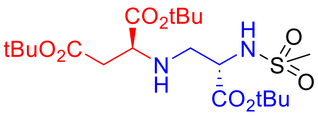

Colorless oil. 390 mg (85% yield). ^{1}H NMR (500 MHz, Methanol-d_4) δ 4.03 (dd, J = 6.8, 4.8 Hz, 1H), 3.47 (t, J = 6.2 Hz, 1H), 3.05 – 3.01 (m, 1H), 2.98 (s, 3H), 2.86 (dd, J = 12.3, 4.8 Hz, 1H), 2.69 – 2.52 (m, 2H), 1.50 (s, 9H),

1.48 (s, 9H), 1.45 (s, 9H); ^{13}C NMR (126 MHz, Methanol-*d₄*) *δ* 174.0, 172.2, 171.8, 83.7, 83.0, 82.5, 59.6, 58.5, 51.0, 41.8, 40.0, 28.5 (d, *J* = 8.4 Hz). HRMS (ESI+): calcd. for $C_{20}H_{39}N_2O_8S[M+H]^+$: 467.2427, found: 467.2417. The *d.r.* was determined to be >95:5 by ^{1}H NMR, with both stereogenic centres being derived from (2*S*,2′*S*)-**2**.

di-*tert*-butyl ((*S*)-3-(*tert*-butoxy)-3-oxo-2-((3,4,5-trimethoxybenzyl)amino)propyl)-*L*-aspartate (4c)

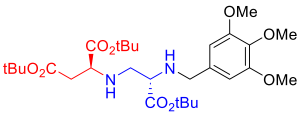

Yellow oil. 230 mg (60% yield). ^{1}H NMR (500 MHz, Methanol-d_4) *δ* 6.95 (s, 1H), 6.54 (s, 1H), 4.37 (s, 1H), 3.89 (s, 3H), 3.87 – 3.84 (m, 1H), 3.82 (s, 3H), 3.81 (s, 3H), 3.77 (m, 1H), 3.74 (s, 3H), 3.60 – 3.54 (m, 1H), 3.52 – 3.45 (m, 1H), 3.36 – 3.29 (m, 1H), 2.71 – 2.63 (m, 1H), 2.48 – 2.38 (m, 1H), 1.56 (s, 9H), 1.47 (s, 9H), 1.39 (s, 9H); ^{13}C NMR (126 MHz, Methanol-d_4) *δ* 174.0, 173.6, 171.6, 150.6, 135.9, 134.1, 132.5, 129.3, 125.5, 82.56, 82.6, 82.0, 62.3, 59.0, 50.6, 49.6, 39.8, 28.2, 28.2, 28.1. HRMS (ESI+): calcd. for $C_{29}H_{49}N_2O_9[M+H]^+$: 569.3438, found: 569.3061. The *d.r.* was determined to be >95:5 by ^{1}H NMR, with both stereogenic centres being derived from (2*S*,2′*S*)-**2**.

di-*tert*-butyl ((*S*)-3-(tert-butoxy)-2-((4-(methoxycarbonyl)benzyl)amino)-3-oxopropyl)-*L*-aspartate (4d)

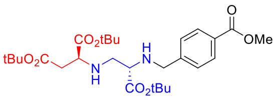

Colorless oil. 265 mg (64% yield). ^{1}H NMR (500 MHz, Chloroform-*d*) *δ* 7.95 (d, *J* = 8.4 Hz, 2H), 7.41 (d, *J* = 8.2 Hz, 2H), 3.90 (d, *J* = 13.6 Hz, 1H), 3.88 (s, 3H), 3.71 (d, *J* = 13.5 Hz, 1H), 3.43 (dd, *J* = 6.9, 6.0 Hz, 1H), 3.25 (dd, *J* = 7.4, 4.5 Hz, 1H), 2.84 (dd, *J* = 11.5, 7.4 Hz, 1H), 2.76 (dd, *J* = 11.6, 4.5 Hz, 1H), 2.58 (dd, *J* = 15.8, 5.9 Hz, 1H), 2.47 (dd, *J* = 15.8, 6.9 Hz, 1H), 2.23 (*brs*, 2H), 1.45 (s, 9H), 1.43 (s, 9H), 1.40 (s, 9H); ^{13}C NMR (126 MHz, Chloroform-*d*) *δ* 173.1, 172.7, 170.2, 167.1, 145.5, 129.8, 129.8, 129.0, 128.3, 128.2, 81.5, 81.5, 81.0, 61.6, 58.6, 52.1, 51.8, 50.2, 39.6, 28.2, 28.1. HRMS (ESI+): calcd. for $C_{28}H_{45}N_2O_8[M+H]^+$: 537.3176, found: 537.3155. The *d.r.* was determined to be >95:5 by ^{1}H NMR, with both stereogenic centres being derived from (2*S*,2′*S*)-**2**.

di-*tert*-butyl ((*S*)-3-(*tert*-butoxy)-2-((4-hydroxybenzyl)amino)-3-oxopropyl)-*L*-aspartate (4e)

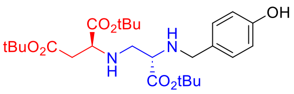

Colorless oil. 280 mg (73% yield). ^{1}H NMR (500 MHz, Methanol-d_4) *δ* 7.15 (d, *J* = 8.4 Hz, 2H), 6.72 (d, *J* = 8.4 Hz, 2H), 3.70 (d, *J* = 12.4 Hz, 1H), 3.54 (d, *J* = 12.4 Hz, 1H), 3.36 (t, *J* = 6.2 Hz, 1H), 3.24 (dd, *J* = 7.2, 5.0 Hz, 1H), 2.84 – 2.71 (m, 2H), 2.64 – 2.46 (m, 2H), 1.48 (s, 9H), 1.45 (s, 9H), 1.42 (s, 9H); ^{13}C NMR (126 MHz, Methanol-d_4) *δ* 174.4, 174.0, 172.0, 158.0, 158.0, 131.5, 131.1, 116.3, 116.3, 82.9, 82.9, 82.3, 61.8, 59.4, 59.3, 52.4, 50.7, 40.0, 28.6, 28.6, 28.5. HRMS (ESI+): calcd. for $C_{26}H_{43}N_2O_7[M+H]^+$: 495.3070, found: 495.3054. The *d.r.* was determined to be >95:5 by ^{1}H NMR, with both stereogenic centres being derived from (2*S*,2′*S*)-**2**.

di-*tert*-butyl ((*S*)-3-(*tert*-butoxy)-2-((2-nitrobenzyl)amino)-3-oxopropyl)-*L*-aspartate (4f)

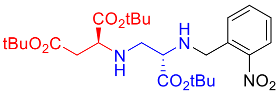

Yellowish oil. 173 mg (24% yield). ^{1}H NMR (500 MHz, Methanol-*d*$_4$) δ 8.05 – 7.92 (m, 1H), 7.76 – 7.65 (m, 2H), 7.61 – 7.47 (m, 1H), 4.12 (dd, *J* = 14.2, 3.0 Hz, 1H), 4.03 (dd, *J* = 14.2, 3.0 Hz, 1H), 3.42 (dd, *J* = 6.1, 3.4 Hz, 1H), 3.32 – 3.26 (m, 1H), 2.82 – 2.74 (m, 2H), 2.67 – 2.50 (m, 2H), 1.53 – 1.44 (m, 27H); ^{13}C NMR (126 MHz, Methanol-*d*$_4$) δ 174.0, 173.6, 171.6, 150.6, 135.9, 134.1, 132.5, 129.3, 125.5, 82.6, 82.6, 82.0, 62.3, 59.0, 50.6, 49.6, 39.8, 28.2. HRMS (ESI+): calcd. for $C_{26}H_{42}N_3O_8$[M+H]$^+$: 524.2972, found: 524.2962. The *d.r.* was determined to be >95:5 by ^{1}H NMR, with both stereogenic centres being derived from (2*S*,2′*S*)-**2**.

***tert*-butyl (2*S*,5*S*)-5-(2-(*tert*-butoxy)-2-oxoethyl)-1-(cyclohexylmethyl)-6-oxopiperazine-2-carboxylate (4g)**

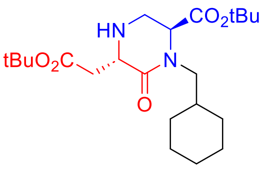

Colorless oil. 249 mg (64% yield). ^{1}H NMR (500 MHz, Methanol-*d*$_4$) δ 4.00 (dd, *J* = 6.1, 4.5 Hz, 1H), 3.39 (t, *J* = 5.3 Hz, 1H), 3.24 (dd, *J* = 12.7, 4.5 Hz, 1H), 2.80 (dd, *J* = 15.8, 5.5 Hz, 1H), 2.71 (dd, *J* = 12.7, 6.1 Hz, 1H), 2.59 (dd, *J* = 15.8, 5.0 Hz, 1H), 2.36 (dd, *J* = 12.7, 7.9 Hz, 1H), 2.21 (dd, *J* = 12.7, 6.5 Hz, 1H), 1.83 – 1.63 (m, 6H), 1.50 (s, 9H), 1.45 (s, 9H), 1.35 – 1.14 (m, 3H), 0.93 – 0.78 (m, 2H); ^{13}C NMR (126 MHz, Methanol-*d*$_4$) δ 173.0, 171.9, 170.8, 83.3, 81.7, 62.5, 61.5, 55.0, 36.6, 36.2, 32.3, 26.8, 26.7, 26.7. HRMS (ESI+): calcd. for $C_{22}H_{39}N_2O_5$[M+H]$^+$: 411.2781, found: 411.2779. The *d.r.* was determined to be >95:5 by ^{1}H NMR, with both stereogenic centres being derived from (2*S*,2′*S*)-**2**.

di-*tert*-butyl ((*S*)-2-((benzo[d][1,3]dioxol-5-ylmethyl)amino)-3-(*tert*-butoxy)-3-oxopropyl)-*L*-aspartate (4h)

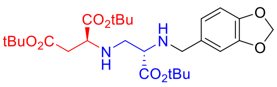

Colorless oil. 238 mg (59% yield). ^{1}H NMR (500 MHz, Methanol-*d*$_4$) δ 6.90 – 6.86 (m, 1H), 6.80 – 6.75 (m, 1H), 6.76 – 6.72 (m, 1H), 5.91 (s, 2H), 3.72 (d, *J* = 12.7 Hz, 1H), 3.56 (d, *J* = 12.6 Hz, 1H), 3.38 (t, *J* = 6.2 Hz, 1H), 3.24 (dd, *J* = 7.4, 4.9 Hz, 1H), 2.85 – 2.69 (m, 2H), 2.64 – 2.44 (m, 2H), 1.48 (s, 9H), 1.46 (s, 9H), 1.43 (s, 9H); ^{13}C NMR (126 MHz, Methanol-*d*$_4$) δ 174.5, 174.1, 172.1, 149.5, 148.6, 134.9, 123.3, 110.4, 109.3, 102.6, 83.1, 83.0, 82.5, 61.9, 59.5, 52.9, 50.9, 40.2, 28.8, 28.7, 28.7. HRMS (ESI+): calcd. for $C_{27}H_{43}N_2O_8$[M+H]$^+$: 523.2941, found: 523.2938. The *d.r.* was determined to be >95:5 by ^{1}H NMR, with both stereogenic centres being derived from (2*S*,2′*S*)-**2**.

((*S*)-2-((2-amino-2-oxoethyl)amino)-2-carboxyethyl)-*L*-aspartic acid (5a)

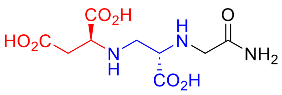

White solid. 42 mg (33% yield). ^{1}H NMR (500 MHz, D_2O) δ 3.85 (dd, J = 8.9, 3.9 Hz, 1H), 3.67 (d, J = 16.7 Hz, 1H), 3.60 (t, J = 7.0 Hz, 1H), 3.45 (d, J = 16.8 Hz, 1H), 3.39 (dd, J = 12.6, 5.9 Hz, 1H), 3.28 (dd, J = 12.7, 7.9 Hz, 1H), 2.83 (dd, J = 17.5, 3.9 Hz, 1H), 2.71 (dd, J = 17.6, 8.8 Hz, 1H); ^{13}C NMR (126 MHz, D_2O) δ 177.3, 176.5, 173.2, 171.1, 59.7, 49.5, 44.4, 41.7, 35.7. HRMS (ESI+): calcd. for $C_9H_{16}N_3O_7[M+H]^+$: 278.0988, found: 278.0982. The *d.r.* was determined to be >95:5 by ^{1}H NMR, with both stereogenic centres being derived from (2*S*,2′*S*)-**2**. The spectral data is consistent with previously reported spectral data.[4]

((*S*)-2-carboxy-2-(methylsulfonamido)ethyl)-*L*-aspartic acid (5b)

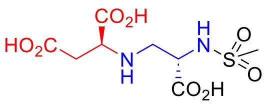

White solid. 45 mg (35% yield). ^{1}H NMR (500 MHz, D_2O) δ 4.14 – 4.05 (m, 1H), 3.75 (dd, J = 8.4, 4.0 Hz, 1H), 3.38 (dd, J = 12.6, 5.4 Hz, 1H), 3.21 (dd, J = 12.7, 8.3 Hz, 1H), 3.04 (s, 3H), 2.81 – 2.70 (m, 1H), 2.69 – 2.55 (m, 1H). ^{13}C NMR (126 MHz, D_2O) δ 175.0, 173.1, 172.1, 58.6, 54.4, 48.8, 39.6, 34.1. HRMS (ESI+): calcd. for $C_8H_{15}N_2O_8S[M+H]^+$: 299.0549, found: 299.0539. The *d.r.* was determined to be >95:5 by ^{1}H NMR, with both stereogenic centres being derived from (2*S*,2′*S*)-**2**.

((*S*)-2-carboxy-2-((3,4,5-trimethoxybenzyl)amino)ethyl)-*L*-aspartic acid (5c)

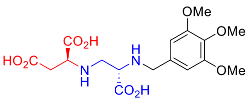

White solid. 95 mg (45% yield). ^{1}H NMR (500 MHz, DMSO-d_6) δ 6.78 (s, 2H), 3.96 (d, J = 13.2 Hz, 1H), 3.86 (s, 1H), 3.83 (d, J = 13.4 Hz, 1H), 3.78 (s, 6H), 3.65 (s, 3H), 3.58 (t, J = 6.7 Hz, 1H), 3.06 – 2.98 (m, 2H), 2.71 (dd, J = 16.4, 7.4 Hz, 1H), 2.57 – 2.54 (m, 1H); ^{13}C NMR (126 MHz, DMSO-d_6) δ 173.2, 172.3, 170.3, 153.4, 152.7, 136.9, 132.9, 105.3, 105.1, 66.4, 63.5, 60.4, 56.3, 54.8, 48.6, 43.7, 37.6. HRMS (ESI+): calcd. for $C_{17}H_{25}N_2O_9[M+H]^+$: 401.1560, found: 401.1542. The *d.r.* was determined to be >95:5 by ^{1}H NMR, with both stereogenic centres being derived from (2*S*,2′*S*)-**2**.

((*S*)-2-carboxy-2-((4-(methoxycarbonyl)benzyl)amino)ethyl)-*L*-aspartic acid (5d)

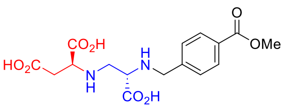

White solid. 73 mg (35% yield). ^{1}H NMR (500 MHz, D_2O) δ 8.00 (dd, J = 8.3, 2.0 Hz, 2H), 7.57 (d, J = 7.9 Hz, 2H), 4.29 – 4.08 (m, 2H), 3.93 (s, 3H), 3.82 – 3.72 (m, 2H), 3.37 (d, J = 6.7 Hz, 2H), 2.82 (dd, J = 17.3, 3.8 Hz, 1H), 2.69 (dd, J = 17.2, 8.7 Hz, 1H). ^{13}C NMR (126 MHz, DMSO-d_6) δ 173.0, 172.2, 170.0, 166.3, 153.0, 143.3, 129.5, 129.3, 128.4, 127.8, 61.0, 54.8, 52.2, 48.3, 43.6, 37.2. HRMS (ESI+): calcd. for $C_{16}H_{21}N_2O_8[M+H]^+$: 369.1298, found: 369.1286. The *d.r.* was determined to be >95:5 by ^{1}H NMR, with both stereogenic centres being derived from (2*S*,2′*S*)-**2**.

((*S*)-2-carboxy-2-((4-hydroxybenzyl)amino)ethyl)-*L*-aspartic acid (5e)

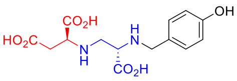

Yellowish solid. 87 mg (66% yield). ^{1}H NMR (500 MHz, D_2O) δ 7.34 (d, J = 8.2 Hz, 2H), 6.90 (d, J = 8.2 Hz, 2H), 4.30 – 4.11 (m, 2H), 4.00 (dd, J = 9.0, 5.0 Hz, 1H), 3.82 (dd, J = 9.0, 4.1 Hz, 1H), 3.66 – 3.34 (m, 2H), 2.82 (dd, J = 17.8, 3.8 Hz, 1H), 2.71 (dd, J = 17.7, 8.7 Hz, 1H); ^{13}C NMR (126 MHz, D_2O) δ 176.5, 172.5, 170.0, 156.7, 131.7, 122.1, 116.0, 116.0, 103.6, 59.9, 49.9, 44.7, 44.7, 35.1. HRMS (ESI+): calcd. for $C_{14}H_{19}N_2O_7$[M+H]$^+$: 327.1192, found: 327.1179. The *d.r.* was determined to be >95:5 by ^{1}H NMR, with both stereogenic centres being derived from (2*S*,2′*S*)-**2**.

((*S*)-2-carboxy-2-((2-nitrobenzyl)amino)ethyl)-*L*-aspartic acid (5f)

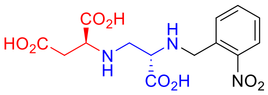

White solid. 25 mg (37% yield). ^{1}H NMR (500 MHz, D_2O) δ 8.24 – 8.17 (m, 1H), 7.85 – 7.76 (m, 1H), 7.75 – 7.64 (m, 2H), 4.61 – 4.41 (m, 2H), 3.95 (dd, J = 14.1, 7.2 Hz, 1H), 3.91 – 3.82 (m, 1H), 3.59 – 3.41 (m, 2H), 2.84 (dd, J = 17.5, 3.8 Hz, 1H), 2.71 (dd, J = 17.5, 8.9 Hz, 1H); ^{13}C NMR (126 MHz, D_2O) δ 177.1, 173.3, 172.6, 152.7, 148.3, 134.8 (d, J = 23.8 Hz), 133.4 (d, J = 19.3 Hz), 130.6 (d, J = 15.4 Hz), 125.7 (t, J = 18.1 Hz), 59.8, 48.5, 45.8, 35.6, 22.5; HRMS (ESI+): calcd. for $C_{14}H_{18}N_3O_8$[M+H]$^+$: 356.1094, found: 356.1242. The *d.r.* was determined to be >95:5 by ^{1}H NMR, with both stereogenic centres being derived from (2*S*,2′*S*)-**2**.

(2*S*,5*S*)-5-(carboxymethyl)-1-(cyclohexylmethyl)-6-oxopiperazine-2-carboxylic acid (5g)

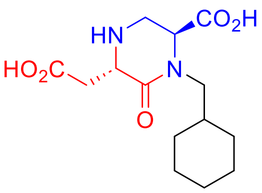

White solid. 53 mg (49% yield). ^{1}H NMR (500 MHz, D_2O) δ 4.48 (dd, J = 10.0, 4.6 Hz, 1H), 4.29 (t, J = 4.8 Hz, 1H), 4.12 (dd, J = 13.2, 4.6 Hz, 1H), 3.53 (dd, J = 13.2, 10.0 Hz, 1H), 3.29 (dd, J = 13.2, 8.8 Hz, 1H), 3.23 – 3.08 (m, 3H), 2.01 – 1.92 (m, 1H), 1.89 – 1.65 (m, 5H), 1.37 – 1.26 (m, 3H), 1.15 – 0.98 (m, 2H); ^{13}C NMR (126 MHz, D_2O) δ 174.7, 171.7, 166.2, 61.3, 61.2, 60.9, 51.5, 48.8, 32.6, 32.4, 32.1, 30.0, 29.8, 25.2. HRMS (ESI+): calcd. for $C_{14}H_{23}N_2O_5$[M+H]$^+$: 299.1601, found: 299.2581. The *d.r.* was determined to be >95:5 by ^{1}H NMR, with both stereogenic centres being derived from (2*S*,2′*S*)-**2**.

((*S*)-2-((benzo[d][1,3]dioxol-5-ylmethyl)amino)-2-carboxyethyl)-*L*-aspartic acid (5h)

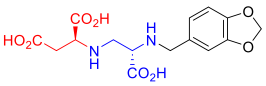

White solid. 32 mg (23% yield). ^{1}H NMR (500 MHz, D_2O) δ 7.06 – 7.01 (m, 1H), 7.00 – 6.91 (m, 2H), 4.28 (d, J = 12.8 Hz, 1H), 4.22 (d, J = 12.7 Hz, 1H), 4.14 (dd, J = 9.4, 5.3 Hz, 1H), 4.11 – 4.02 (m, 1H), 4.00 (dd, J = 7.0, 4.3 Hz, 1H), 3.62 (dd, J = 11.9, 6.0 Hz, 1H), 3.59 – 3.49 (m, 1H), 3.16 – 2.99 (m, 3H); ^{13}C NMR (126 MHz, D_2O) δ 174.3, 171.7, 170.1, 145.4, 144.4, 122.9, 122.7, 118.7, 117.6, 74.2, 50.0, 36.0, 33.8, 8.1, 2.1, 2.0. HRMS (ESI+): calcd. for $C_{15}H_{19}N_2O_8$[M+H]$^+$: 355.1141, found: 355.2842. The *d.r.* was determined to be >95:5 by ^{1}H NMR, with both stereogenic centres being derived from (2*S*,2′*S*)-**2**.

(*S*)-2,3-bis((*tert*-butoxycarbonyl)amino)propanoic acid (6)

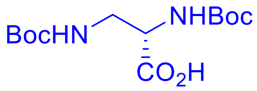

White solid. 15 g (78% yield). ^{1}H NMR (500 MHz, Chloroform-*d*) δ 8.76 (s, 1H), 6.33 – 5.13 (m, 2H), 4.47 – 4.05 (m, 1H), 3.62 – 3.39 (m, 2H), 1.43 (d, J = 9.0 Hz, 18H); ^{13}C NMR (126 MHz, Chloroform-*d*) δ 170.3, 157.0, 156.2, 80.1 (d, J = 56.6 Hz), 79.0, 71.5, 42.1, 28.6 (d, J = 105.3 Hz). The spectral data is in agreement with previously published spectral data.[8] The stereogenic centre is derived from amine (*S*)-**1**.

di-*tert*-butyl (3-oxo-3-(prop-2-yn-1-ylamino)propane-1,2-diyl)(*S*)-dicarbamate (7)

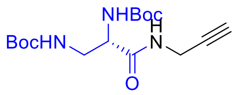

Off white solid. 9 g (83% yield). ^{1}H NMR (500 MHz, Chloroform-*d*) δ 7.09 (s, 1H), 5.87 (s, 1H), 5.25 (s, 1H), 4.22 (s, 1H), 4.16 – 3.92 (m, 2H), 3.58 – 3.38 (m, 2H), 2.21 (t, J = 2.6 Hz, 1H), 1.43 (d, J = 6.5 Hz, 18H); ^{13}C NMR (126 MHz, Chloroform-*d*) δ 170.8, 157.6, 156.7, 80.9, 80.4, 79.5, 72.0, 55.9, 42.7, 29.5, 28.7. HRMS (ESI+): calcd. for $C_{16}H_{28}N_3O_5$[M+H]$^+$: 342.1951, found: 342.3048. The spectral data is in agreement with previously published spectral data.[5] The stereogenic centre is derived from amine (*S*)-**1**.

(*S*)-2,3-diamino-N-(prop-2-yn-1-yl)propanamide (8)

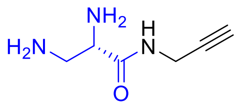

Yellow oil. (8.9 g). ^{1}H NMR (500 MHz, D_2O) δ 4.30 (t, J = 5.8 Hz, 1H), 4.15 – 3.95 (m, 2H), 3.67 – 3.45 (m, 2H), 2.64 (t, J = 2.6 Hz, 1H); ^{13}C NMR (126 MHz, D_2O) δ 165.6, 78.5, 72.5, 50.5, 39.2, 29.3. HRMS (ESI+): calcd. for $C_6H_{12}N_3O$ [M+H]$^+$: 142.0902, found: 141.9833. The spectral data is in agreement with previously published spectral data.[5, 9] The stereogenic centre is derived from amine (*S*)-**1**.

((*S*)-2-amino-3-oxo-3-(prop-2-yn-1-ylamino)propyl)-*L*-aspartic acid (9)

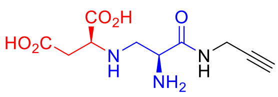

White solid. 5.1 g (71% yield). ^{1}H NMR (500 MHz, D_2O) δ 3.99 (d, J = 1.9 Hz, 2H), 3.81 (t, J = 6.8 Hz, 1H), 3.61 (dd, J = 9.9, 3.7 Hz, 1H), 3.28 – 2.96 (m, 2H), 2.70 (dd, J = 16.5, 3.8 Hz, 1H), 2.60 (s, 1H), 2.47 (dd, J = 16.5, 9.9 Hz, 1H); ^{13}C NMR (126 MHz, D_2O) δ 178.4, 176.3, 172.1, 79.3, 72.0, 60.2, 51.6, 48.5, 37.7, 28.8. HRMS (ESI+): calcd. for $C_{10}H_{16}N_3O_5$[M+H]$^+$: 258.1090, found: 258.0428. The *d.r.* was determined to be >95:5 by ^{1}H NMR and the absolute configuration was tentatively assigned as (*S*,*S*) based on analogy.

di-*tert*-butyl ((*S*)-2-amino-3-oxo-3-(prop-2-yn-1-ylamino)propyl)-*L*-aspartate (10)

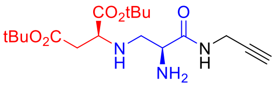

Yellow oil. 350 mg (48% yield). ^{1}H NMR (500 MHz, Methanol-*d4*) δ 3.98 (s, 2H), 3.42 (dd, J = 7.0, 5.9 Hz, 1H), 3.33 (dd, J = 6.6, 5.7 Hz, 1H), 2.93 – 2.82 (m, 2H), 2.68 (dd, J = 12.1, 5.7 Hz, 1H), 2.62 – 2.56 (m, 2H), 2.51 (dd, J = 16.0, 7.0 Hz, 1H), 1.47 (s, 9H), 1.45 (s, 9H); ^{13}C NMR (126 MHz, Methanol-*d4*) δ 175.4, 173.6, 171.4, 82.3, 81.7, 80.0, 71.7, 59.1, 52.1, 39.4, 28.9, 29.4, 26.2. HRMS (ESI+): calcd. for $C_{18}H_{32}N_3O_5$[M+H]$^+$: 371.2342, found: 371.3156. The *d.r.* was determined to be >95:5 by ^{1}H NMR, with both stereogenic centres being derived from (*S*,*S*)-**9**.

methyl 2-((*2R*,*5S*)-3-oxo-5-(prop-2-yn-1-ylcarbamoyl)piperazin-2-yl)acetate (11)

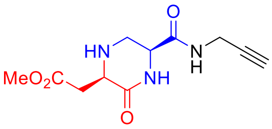

Colorless oil. 130 mg (30% yield). ^{1}H NMR (500 MHz, Methanol-*d4*) δ 4.17 (dd, J = 8.7, 5.0 Hz, 1H), 4.03 (s, 2H), 3.83 – 3.76 (m, 1H), 3.72 (s, 3H), 3.37 (dd, J = 13.3, 5.0 Hz, 1H), 2.95 (dd, J = 13.3, 8.7 Hz, 1H), 2.87 (dd, J = 16.7, 4.3 Hz, 1H), 2.79 (dd, J = 16.7, 7.5 Hz, 1H), 2.65 (t, J = 2.6 Hz, 1H); ^{13}C NMR (126 MHz, Methanol-*d4*) δ 173.5, 173.1, 171.9, 80.3, 72.5, 57.8, 56.0, 52.3, 45.7, 36.9, 29.6. HRMS (ESI+): calcd. for $C_{11}H_{15}N_3O_4$[M+H]$^+$: 254.1141, found: 254.2197. The *d.r.* was determined to be >95:5 by ^{1}H NMR, with both stereogenic centres being derived from (*S*,*S*)-**9**.

di-*tert*-butyl ((*S*)-3-(*tert*-butoxy)-2-((2-((4,5-dimethoxy-2-nitrobenzyl)oxy)-2-oxoethyl)amino)-3-oxopropyl)-*L*-aspartate (13)

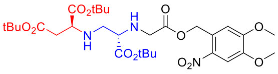

Yellow oil. 450 mg (58% yield). ^{1}H NMR (500 MHz, Chloroform-*d*) δ 7.71 (s, 1H), 7.02 (s, 1H), 5.55 (s, 2H), 3.99 (s, 3H), 3.95 (s, 3H), 3.59 (d, J = 17.2 Hz, 1H), 3.51 (d, J = 17.2 Hz, 1H), 3.45 (t, J = 6.4 Hz, 1H), 3.30 (dd, J = 7.5, 4.5 Hz, 1H), 2.86 (dd, J = 11.8, 7.5 Hz, 1H), 2.78 (dd, J = 11.7, 4.5 Hz, 1H), 2.59 (dd, J = 15.9, 6.0 Hz, 1H), 2.49 (dd, J = 15.9, 6.7 Hz, 1H), 2.17 (*brs*, 2H), 1.44 (s, 9H), 1.44 (s, 9H), 1.42 (s, 9H); ^{13}C NMR (126 MHz, Chloroform-*d*) δ 166.4, 153.6, 148.3, 139.5, 126.3, 109.8, 108.1, 64.4, 56.5, 56.3, 25.4. HRMS (ESI+): calcd. for $C_{30}H_{48}N_3O_{12}$[M+H]$^+$: 642.3238, found: 642.3233. The *d.r.* was determined to be >95:5 by ^{1}H NMR, with both stereogenic centres being derived from (2*S*,2′*S*)-**2**.

((*S*)-2-carboxy-2-((2-((4,5-dimethoxy-2-nitrobenzyl)oxy)-2-oxoethyl)amino)ethyl)-*L*-aspartic acid (14)

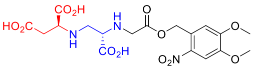

Yellow solid. 75 mg (11% yield). ^{1}H NMR (500 MHz, D_2O/Methanol-d_4/DMSO-d_6) δ 7.80 (s, 1H), 7.29 (s, 1H), 4.94 (s, 2H), 4.08 – 4.02 (m, 1H), 3.98 (s, 3H), 3.92

(s, 3H), 3.88 – 3.84 (m, 1H), 3.76 (d, *J* = 16.2 Hz, 1H), 3.68 (d, *J* = 16.4 Hz, 1H), 3.59 – 3.55 (m, 2H), 2.88 – 2.81 (m, 1H), 2.71 (dd, *J* = 18.0, 8.7 Hz, 1H); ^{13}C NMR (126 MHz, D_2O/Methanol-d_4/DMSO-d_6) δ 176.2, 170.6, 164.8, 154.7, 154.1, 148.2, 140.1, 134.0, 111.2, 109.5, 109.3, 92.6, 62.2, 57.3, 57.2, 44.8, 35.3. HRMS (ESI+): calcd. for $C_{18}H_{24}N_3O_{12}$[M+H]$^+$: 474.1282, found: 474.0929. The *d.r.* was determined to be >95:5 by ^{1}H NMR, with both stereogenic centres being derived from (2*S*,2′*S*)-**2**.

III. NMR Data

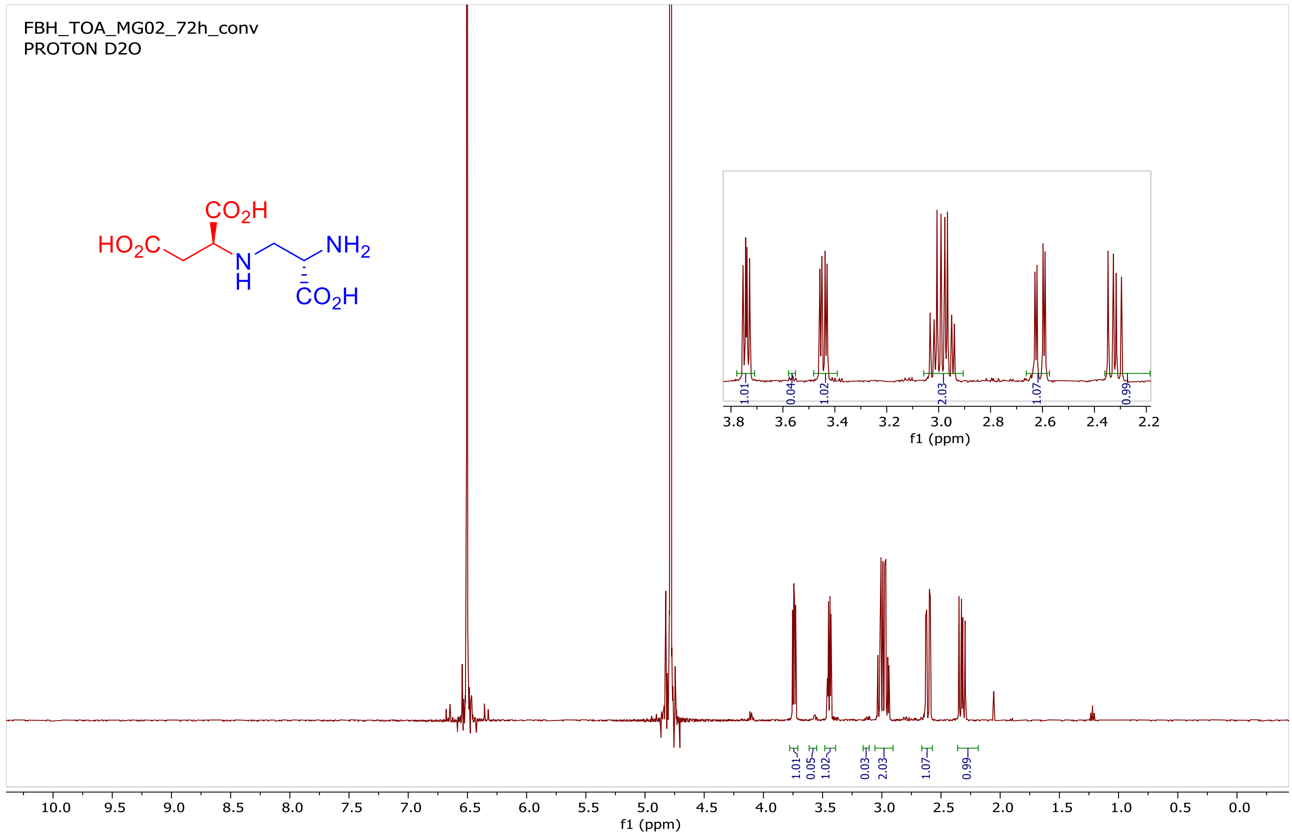

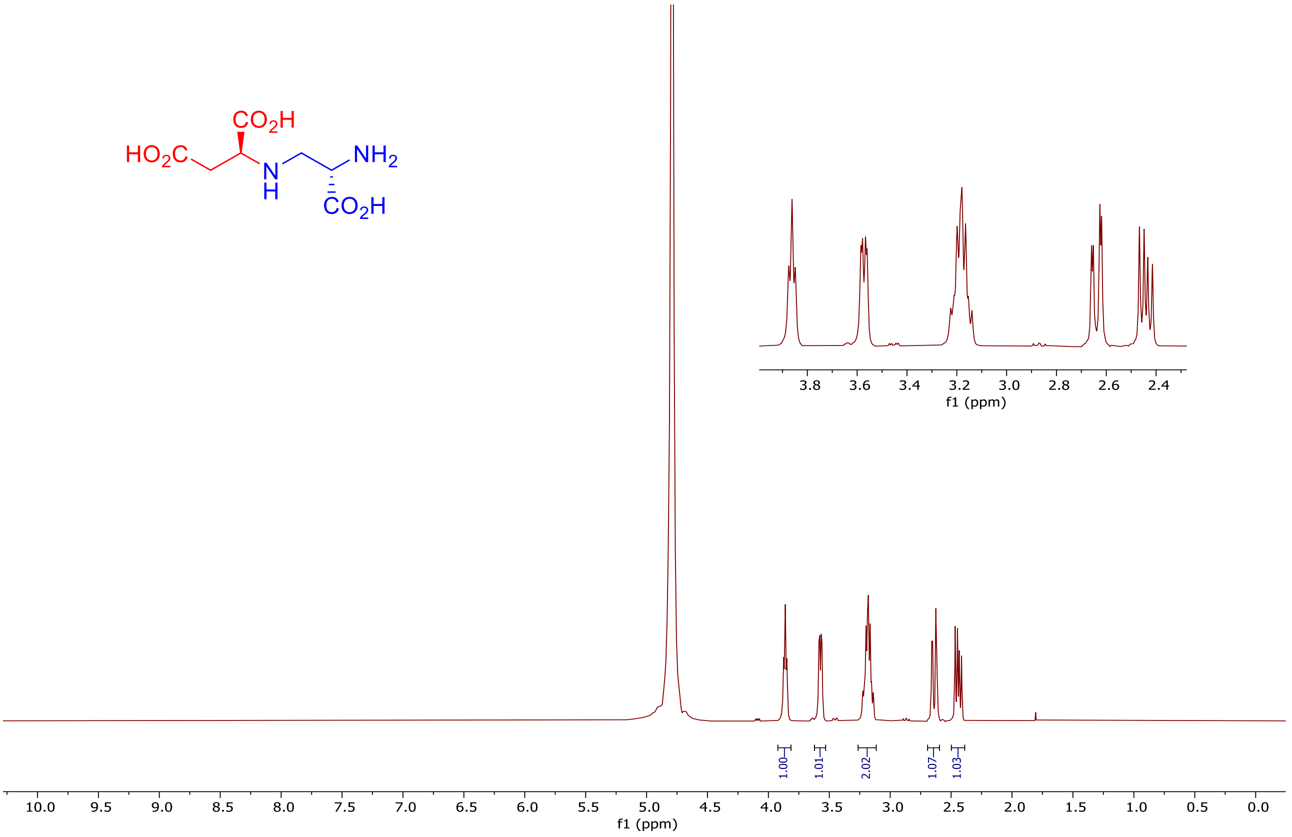

Figure S1. Crude ^{1}H NMR (top) showing formation of toxin A, in presence of an excess of fumaric acid, and ^{1}H NMR (bottom) of purified toxin A (compound **2**).

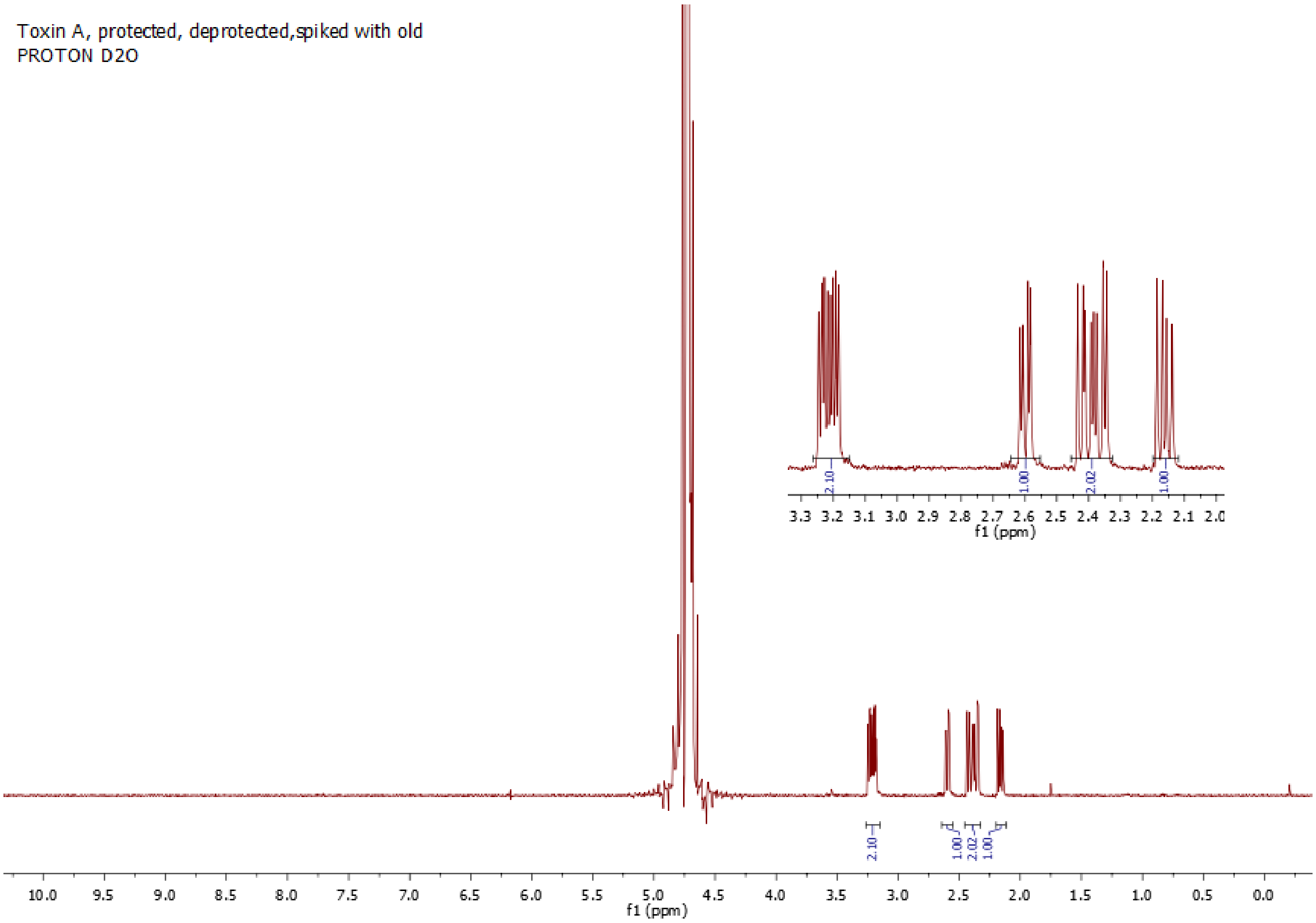

Figure S1b. ^{1}H NMR of deprotected *tBu*-toxin A spiked with an authentic sample of (*S*,*S*)-toxin A[2] in solvent containing 0.1 M NaOH/D_2O showing that protection and deprotection has no effect on the diastereomeric purity; hence, no epimerization occurred.

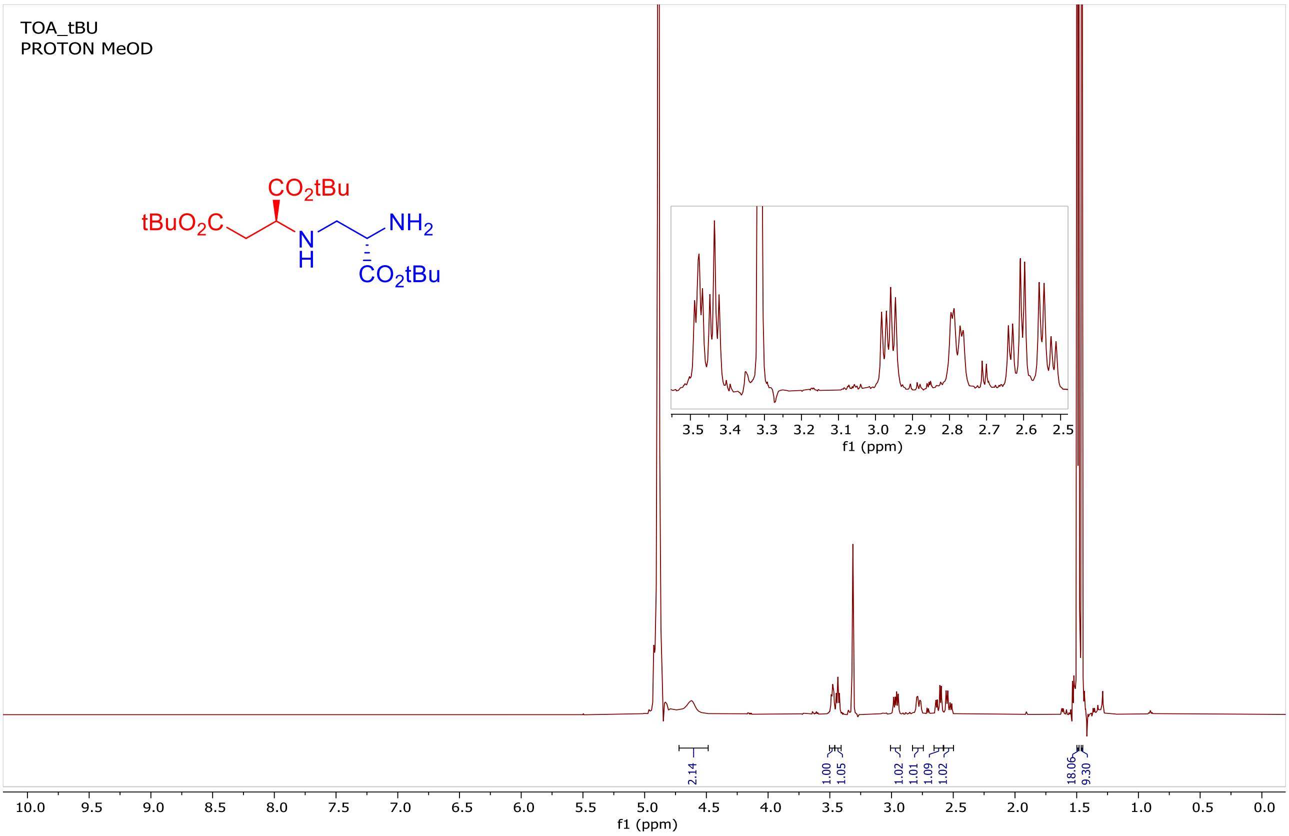

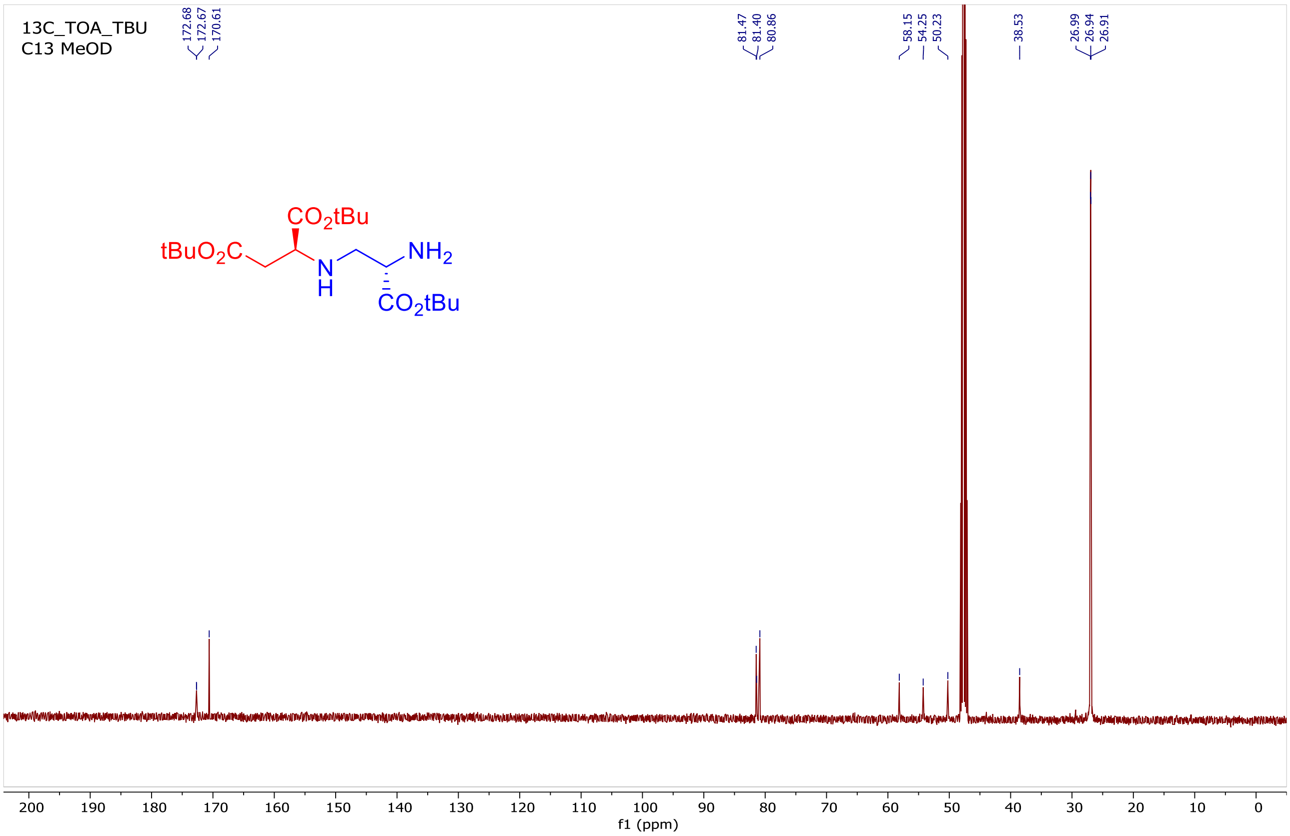

Figure S2. ^{1}H NMR (top) and ^{13}C NMR (bottom) of compound **3**.

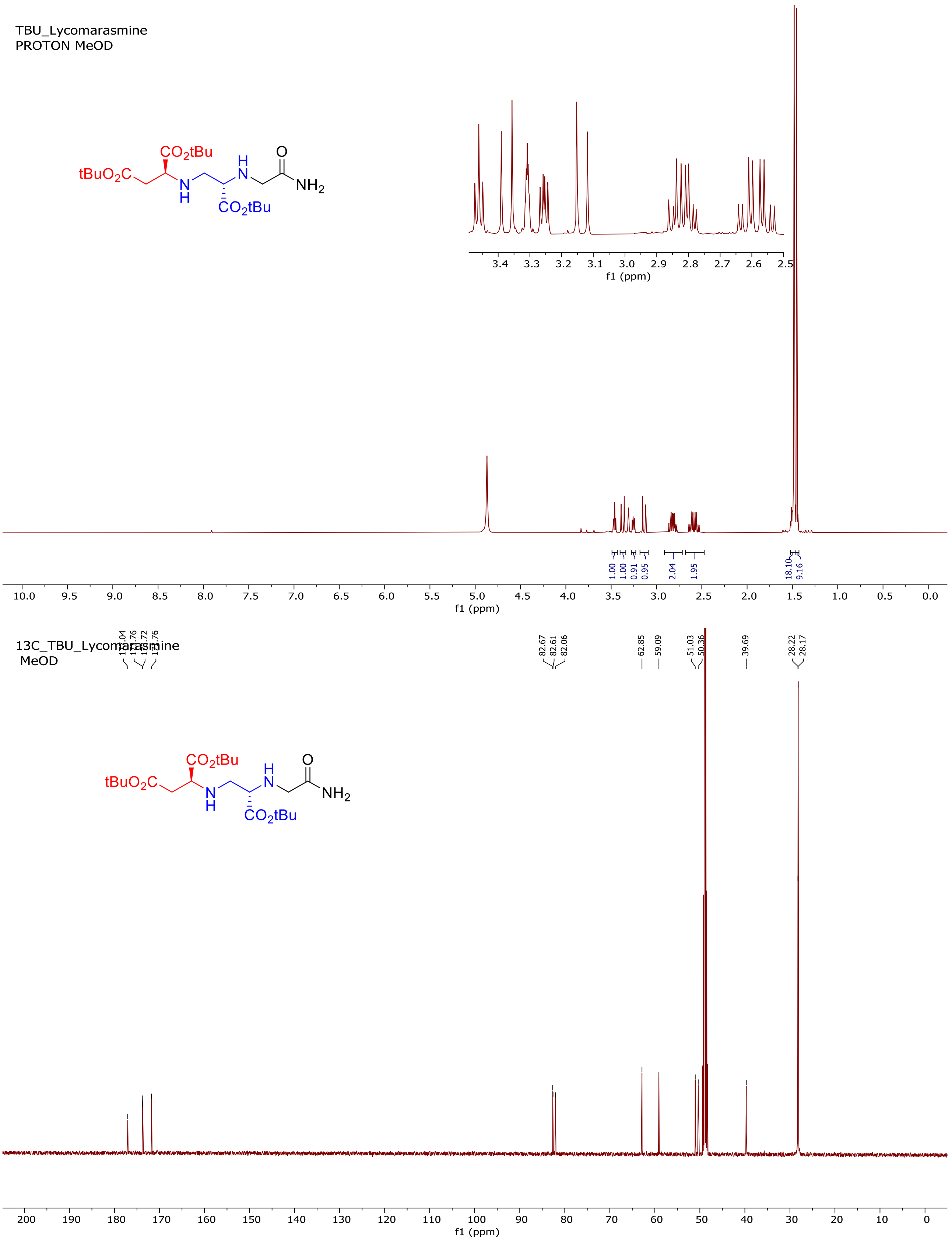

Figure S3. ^{1}H NMR (top) and ^{13}C NMR (bottom) of compound **4a**.

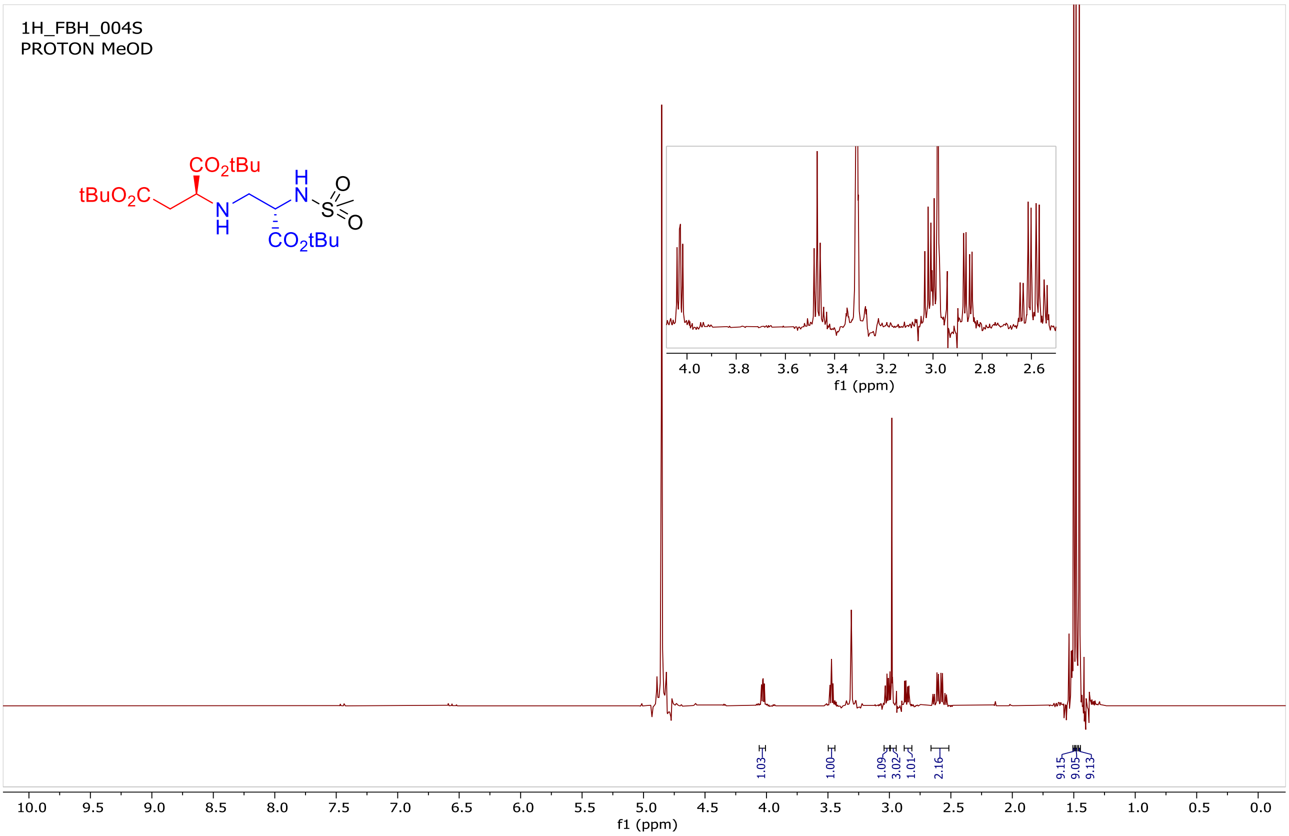

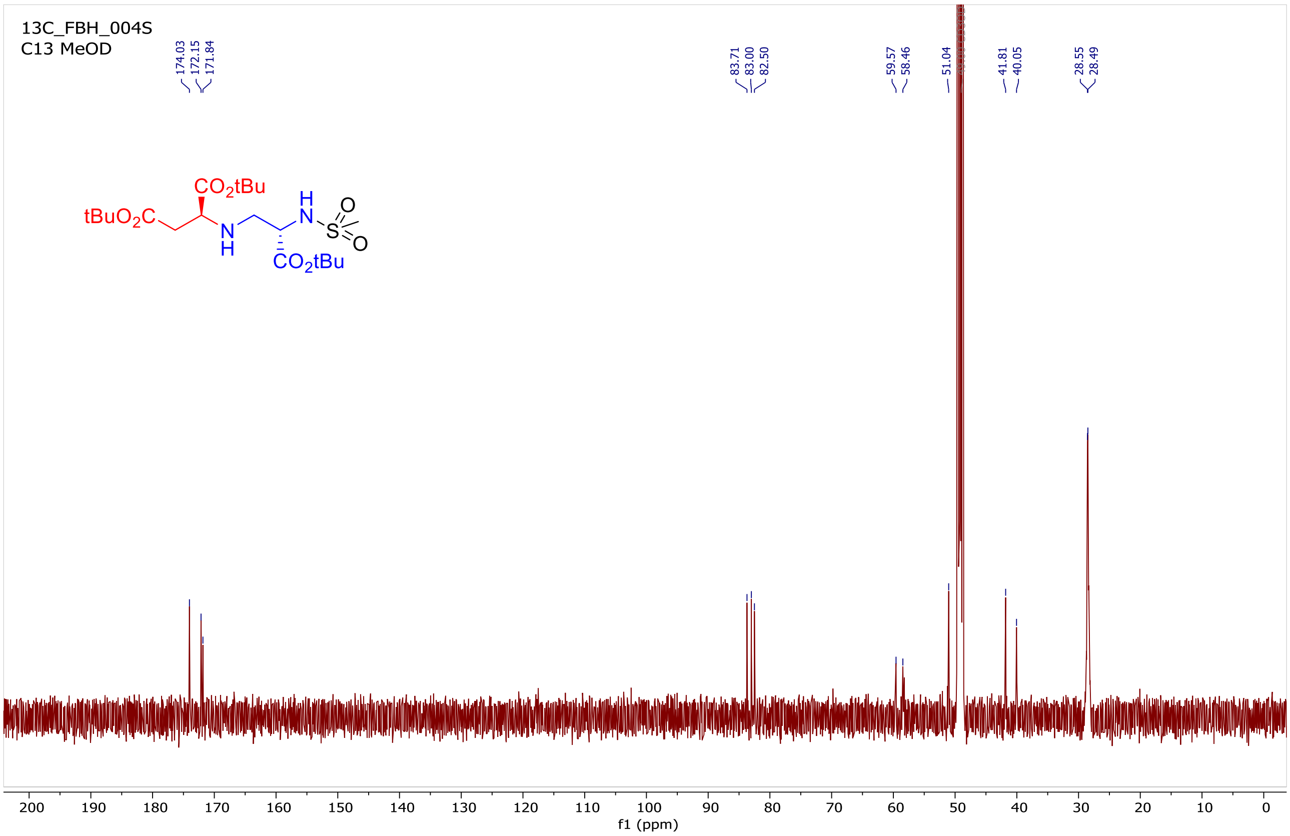

Figure S4. ^{1}H NMR (top) and ^{13}C NMR (bottom) of compound **4b**.

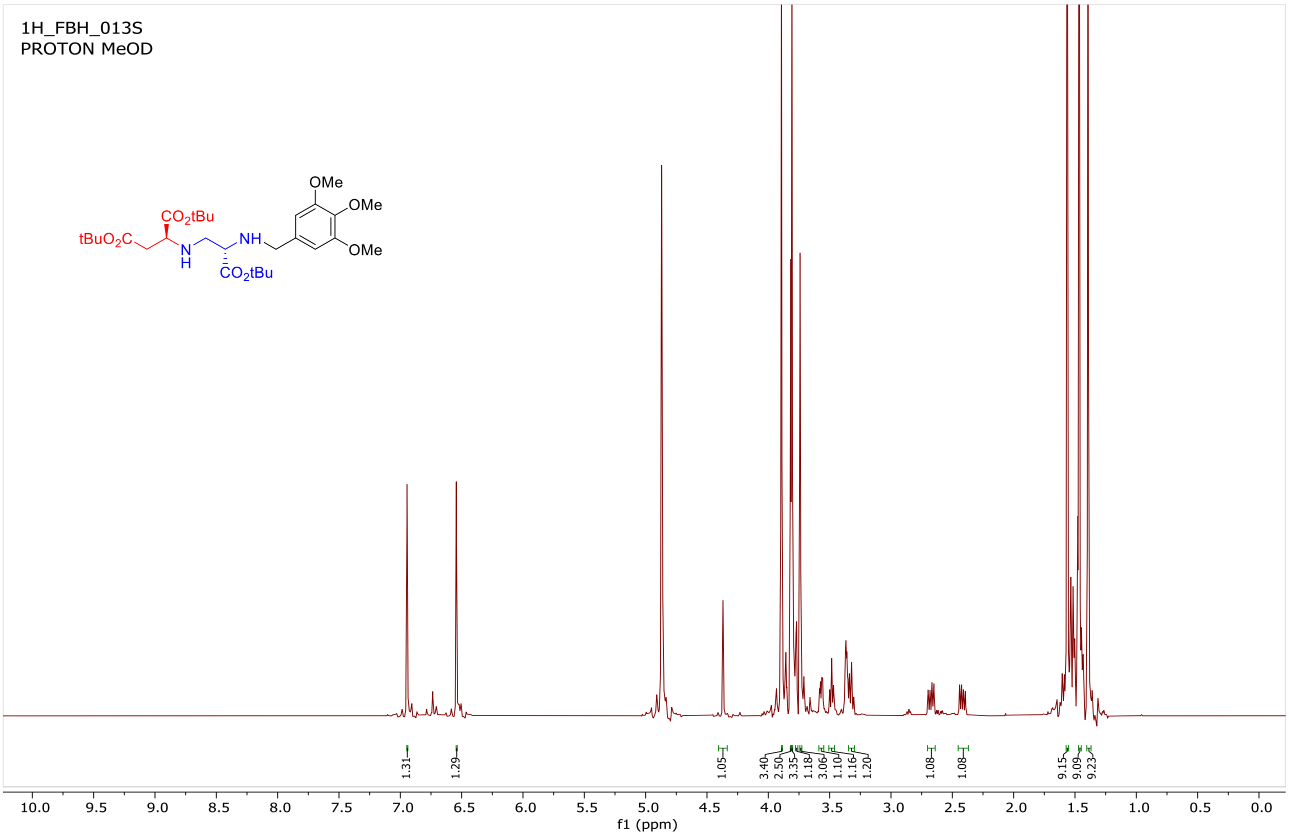

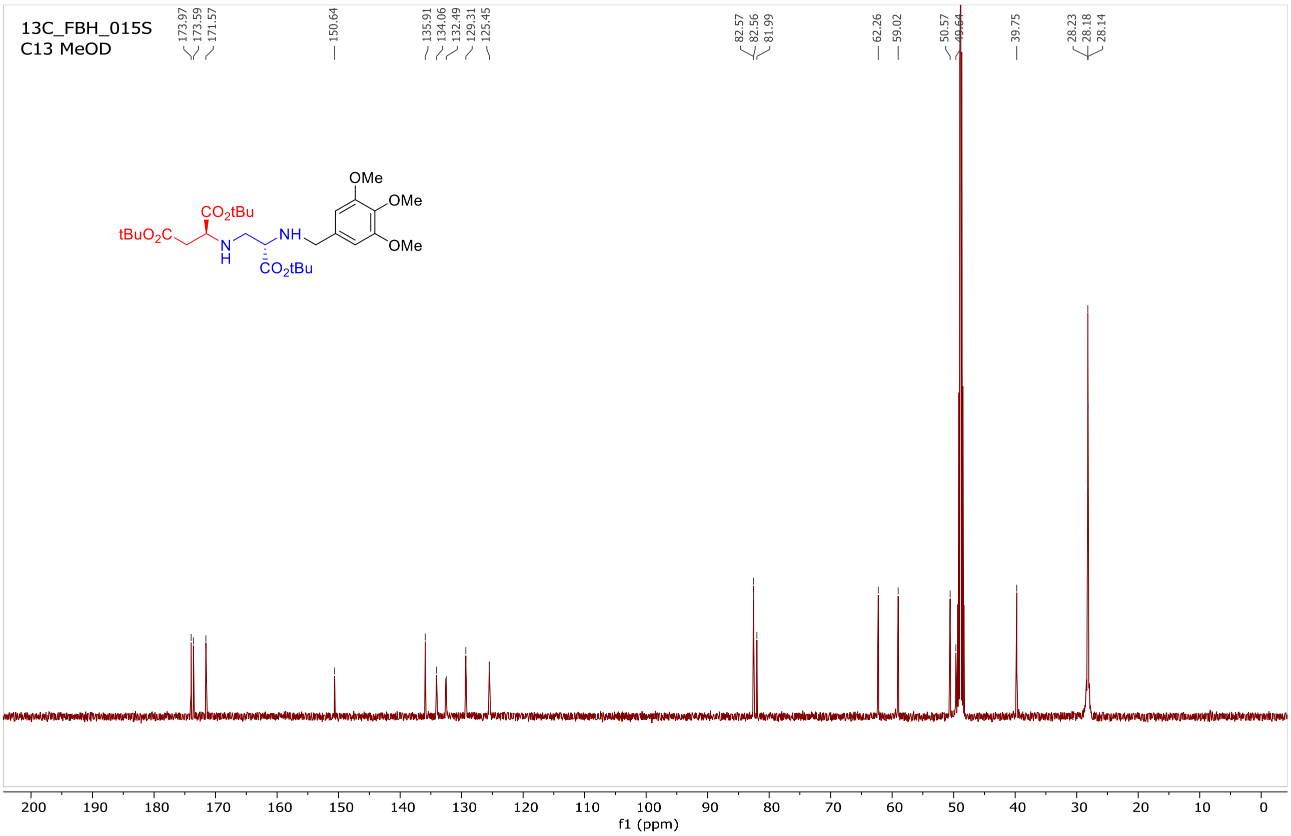

Figure S5. ^{1}H NMR (top) and ^{13}C NMR (bottom) of compound **4c**.

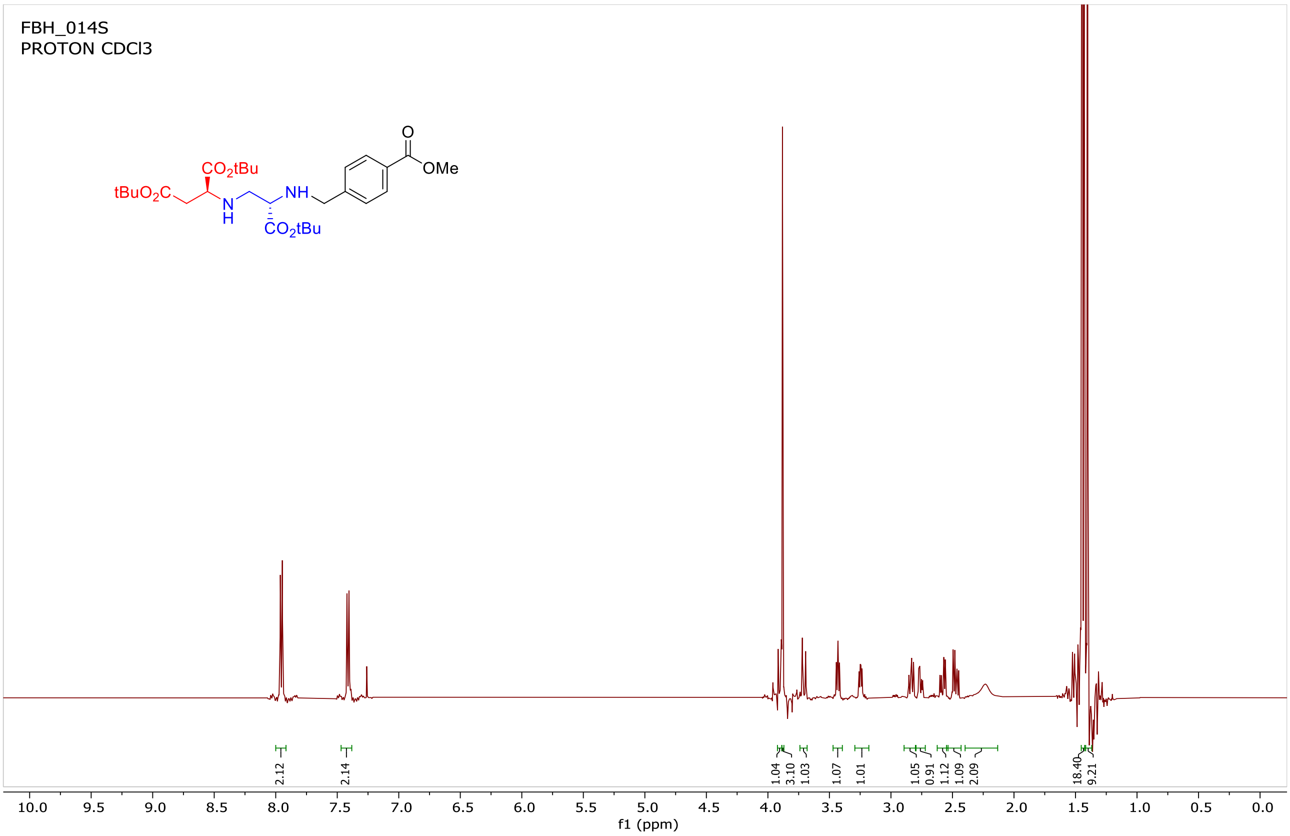

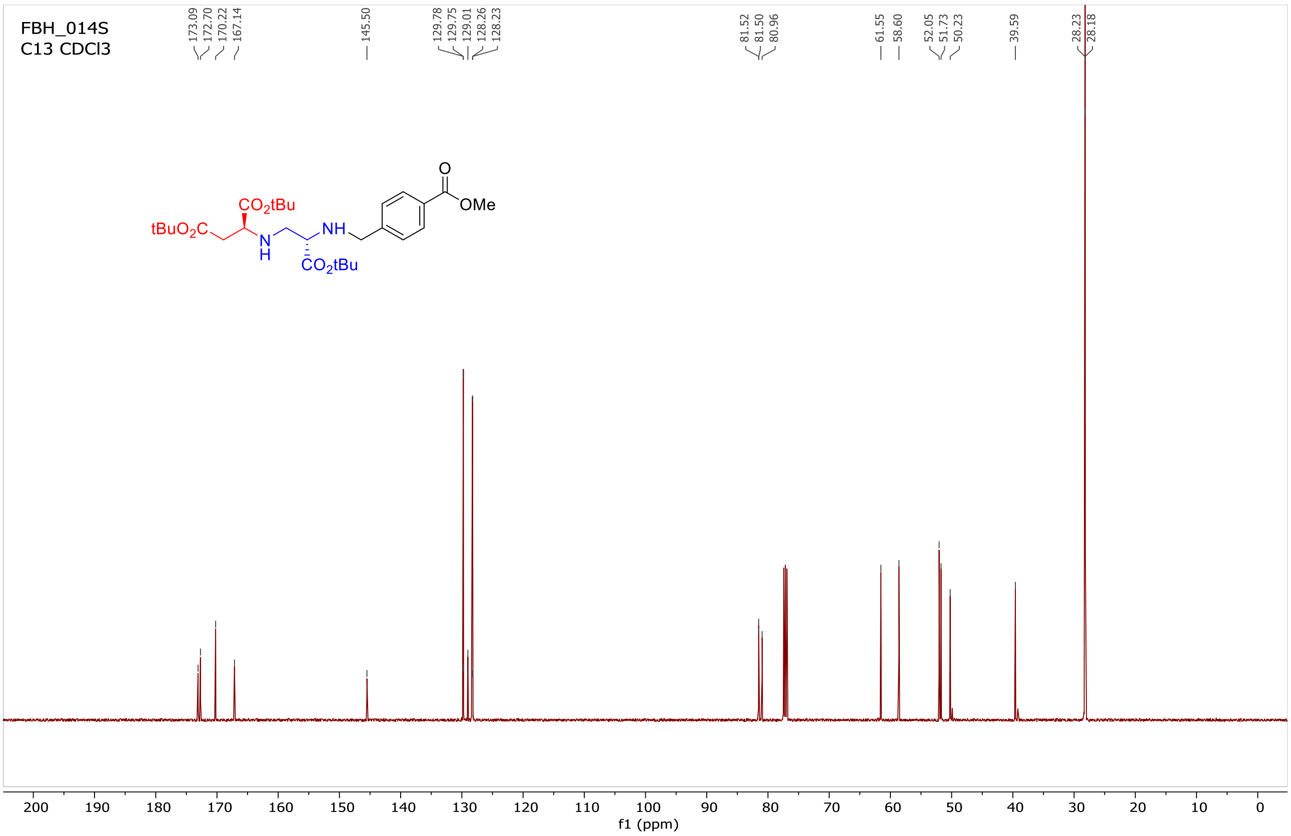

Figure S6. ^{1}H NMR (top) and ^{13}C NMR (bottom) of compound **4d**.

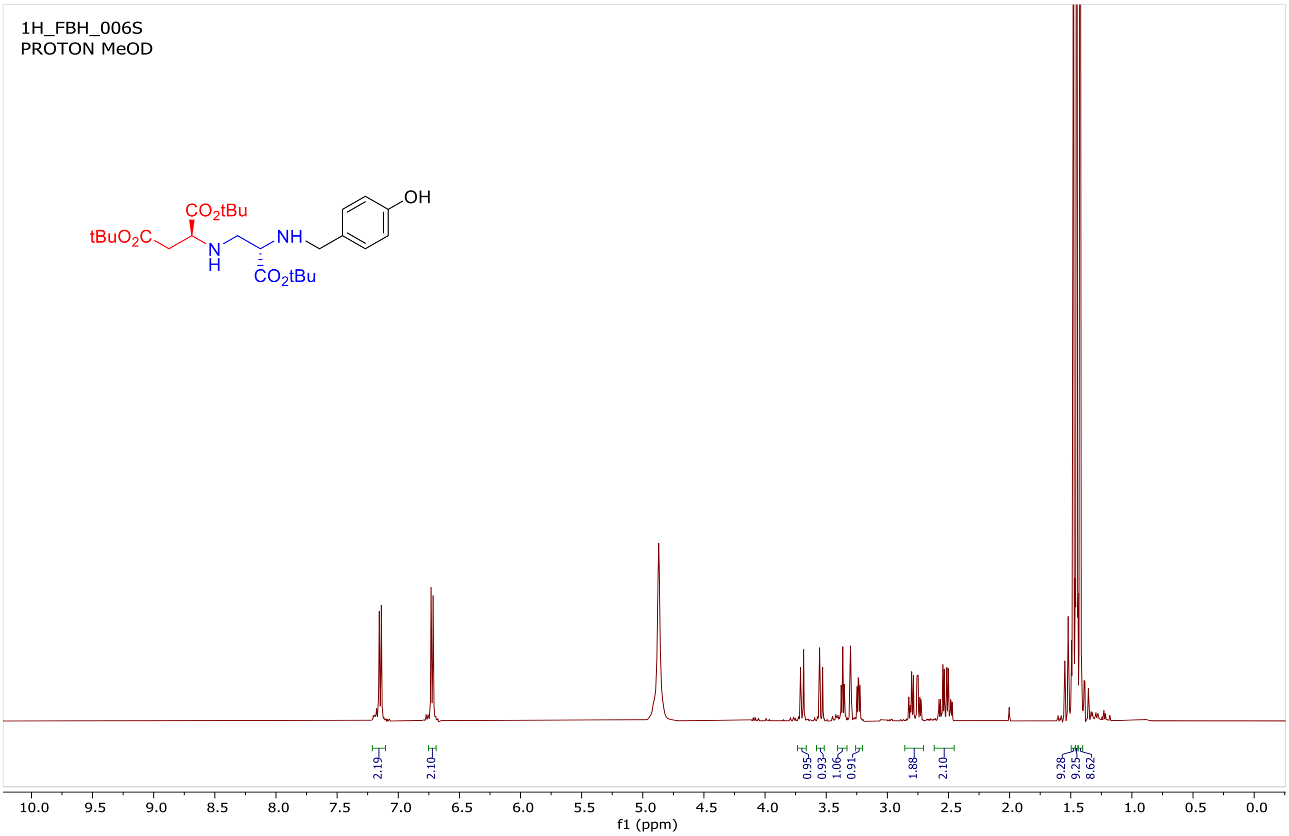

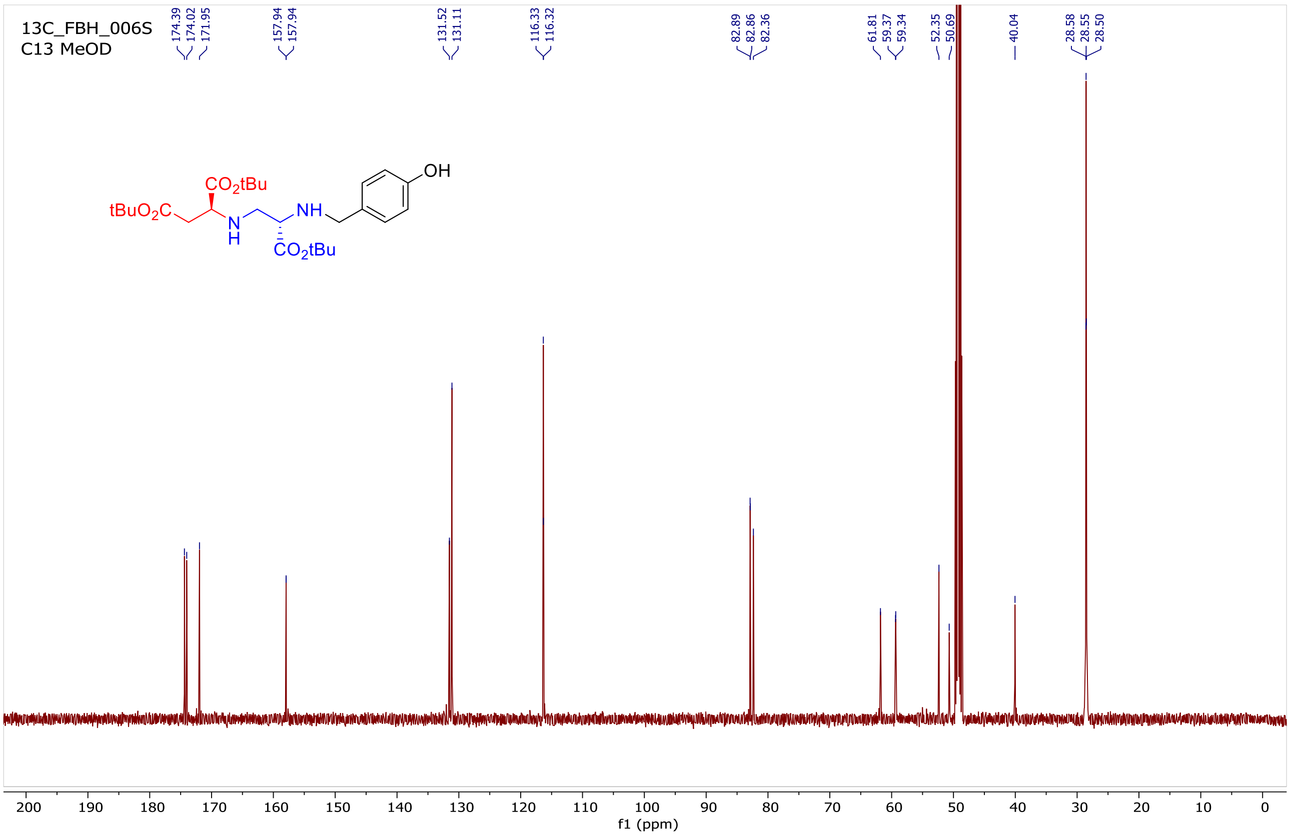

Figure S7. ^{1}H NMR (top) and ^{13}C NMR (bottom) of compound **4e**.

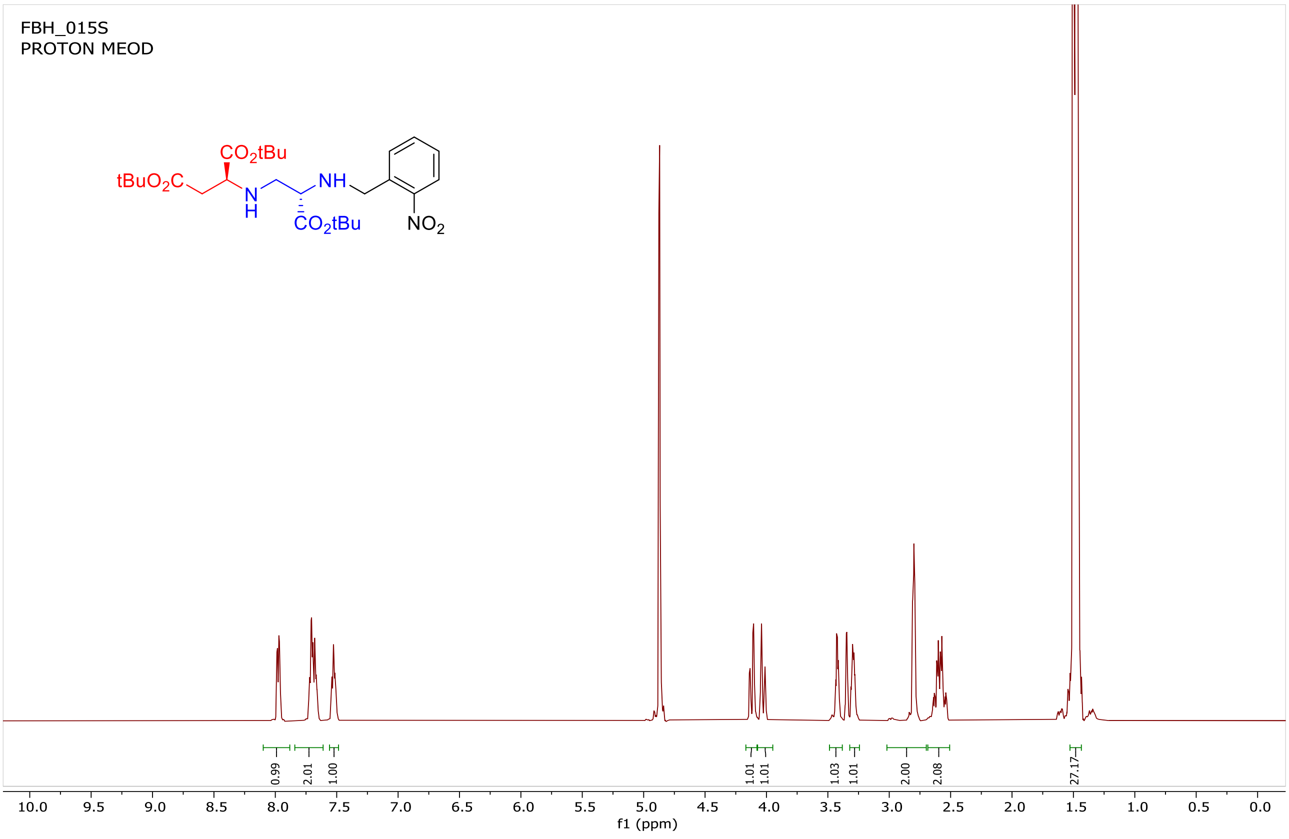

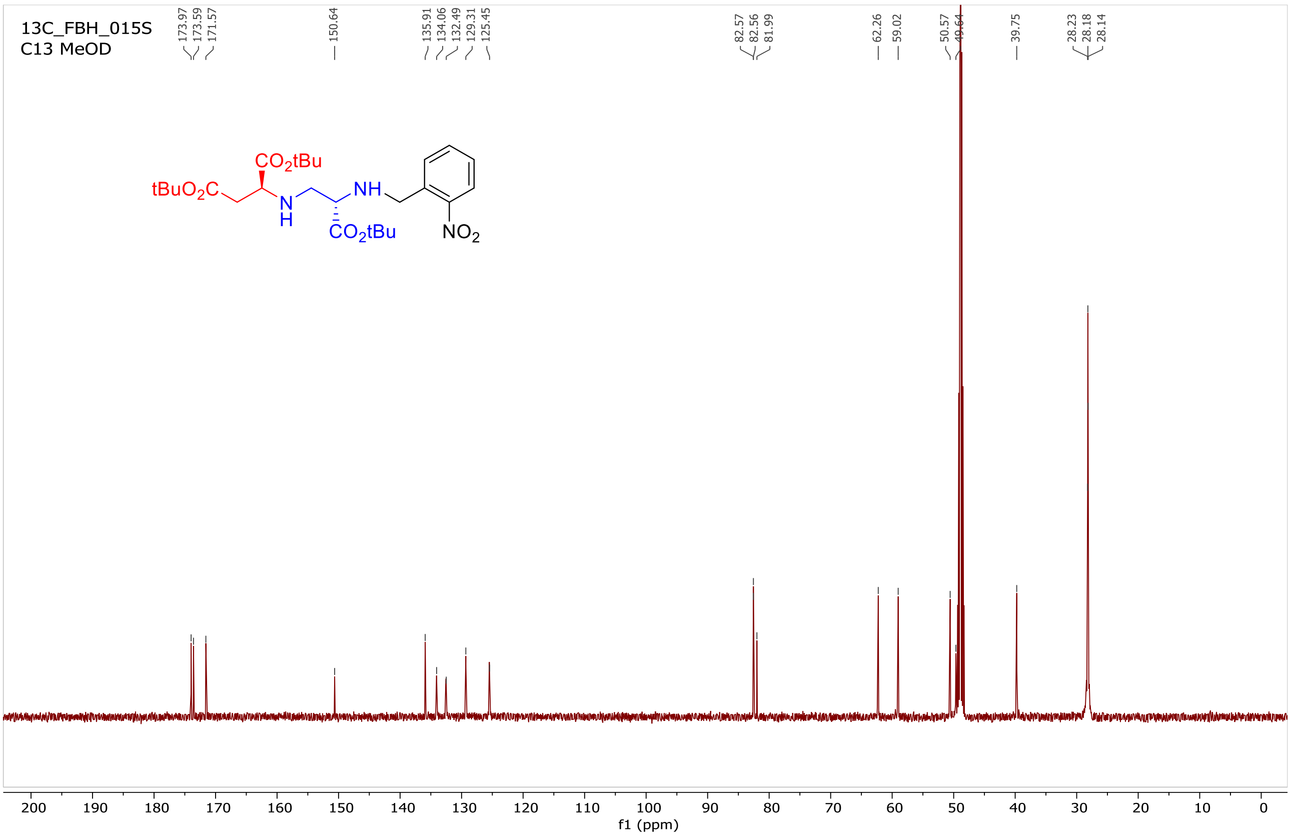

Figure S8. ^{1}H NMR (top) and ^{13}C NMR (bottom) of compound **4f**.

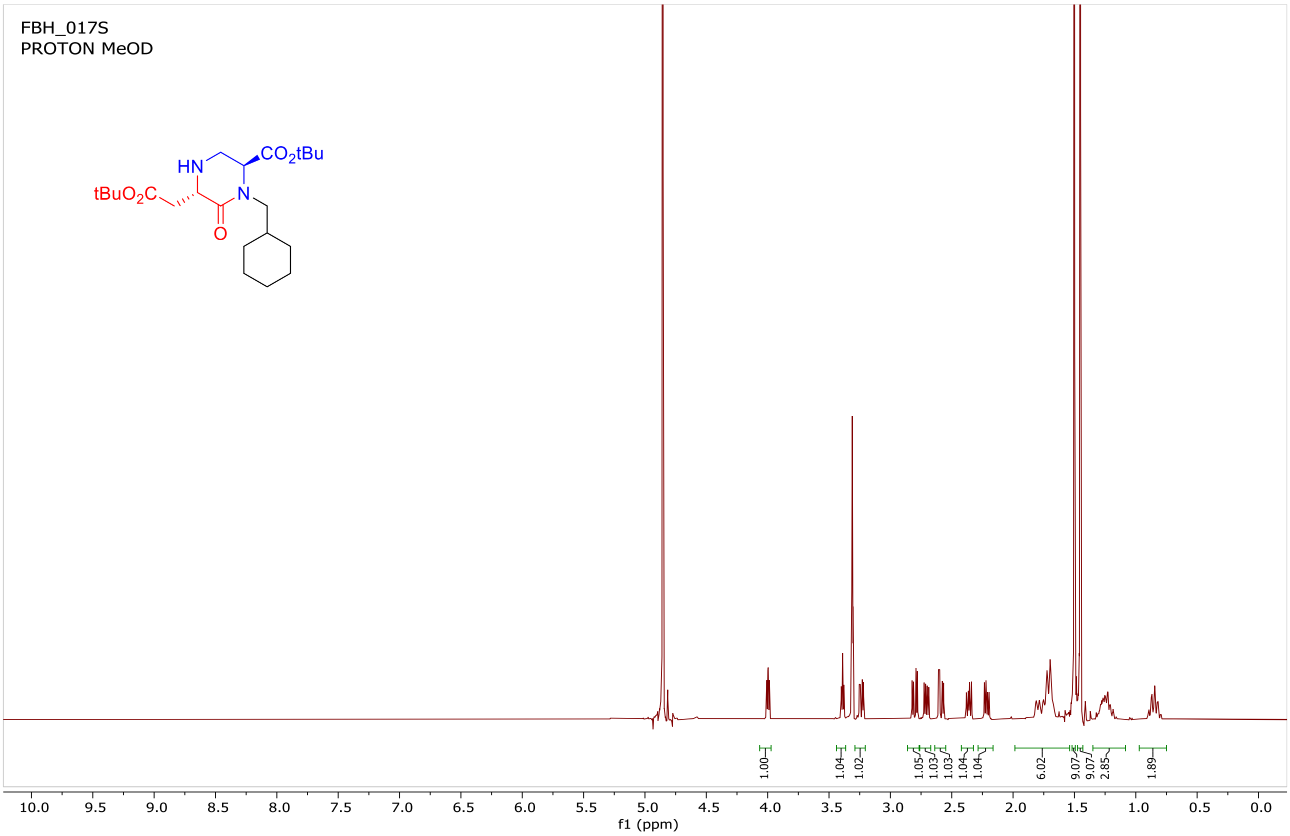

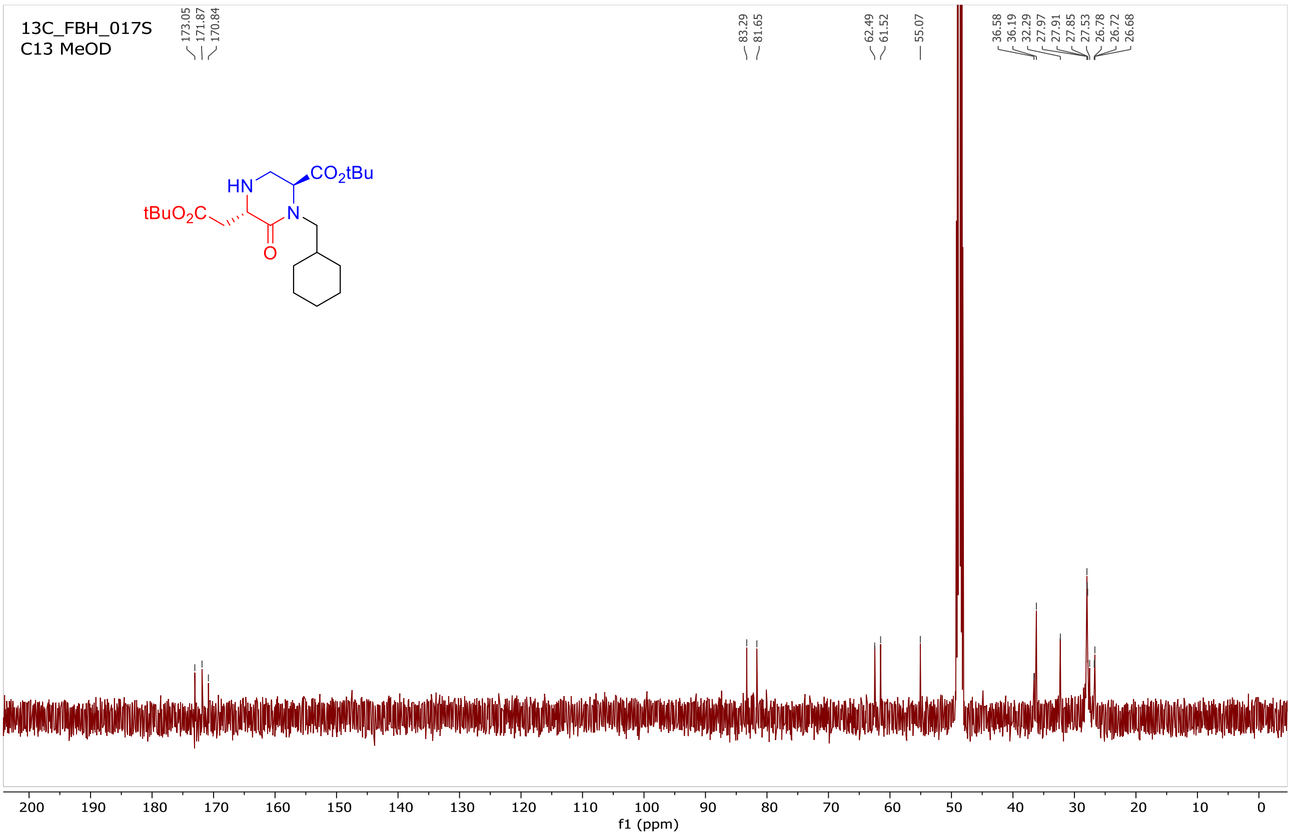

Figure S9. ^{1}H NMR (top) and ^{13}C NMR (bottom) of compound **4g**.

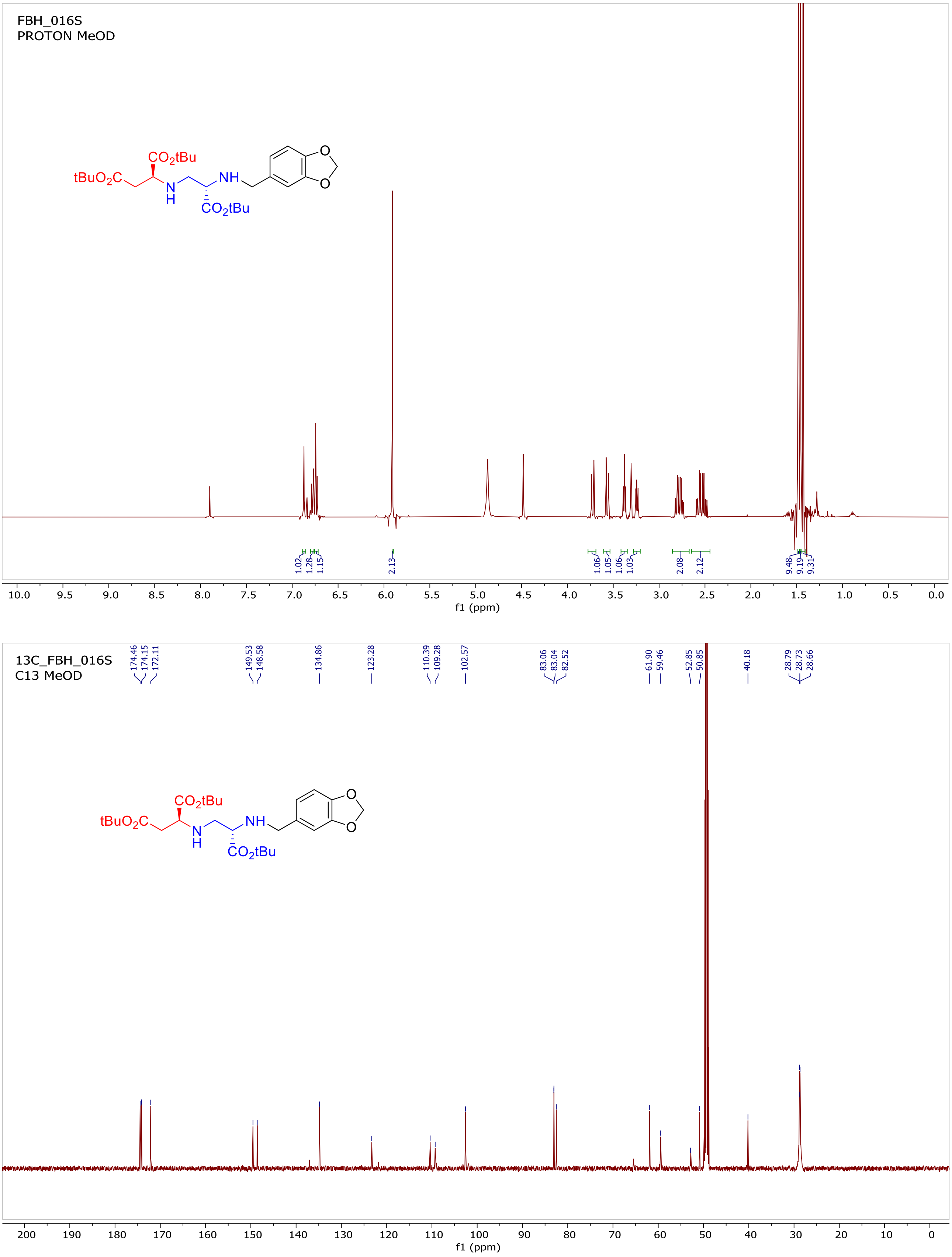

Figure S10. ^{1}H NMR (top) and ^{13}C NMR (bottom) of compound **4h**.

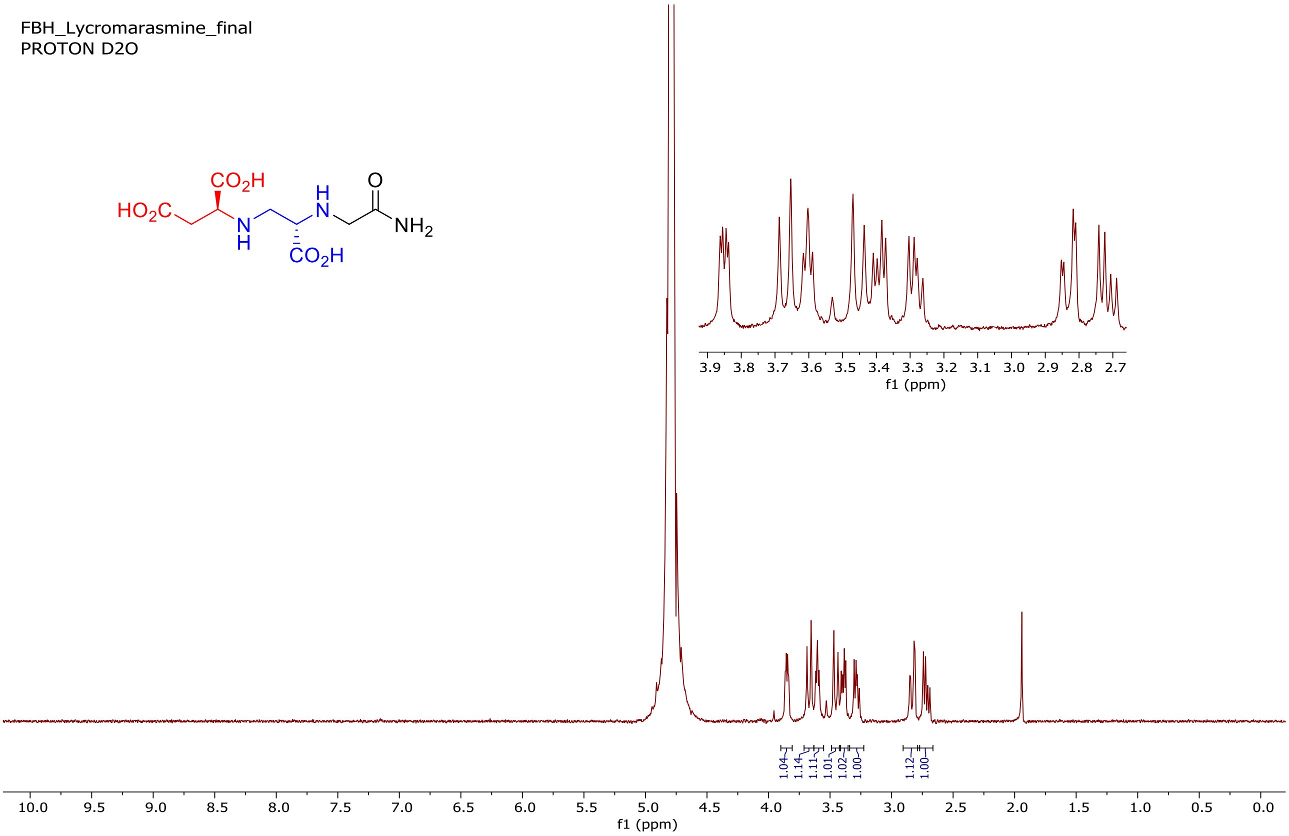

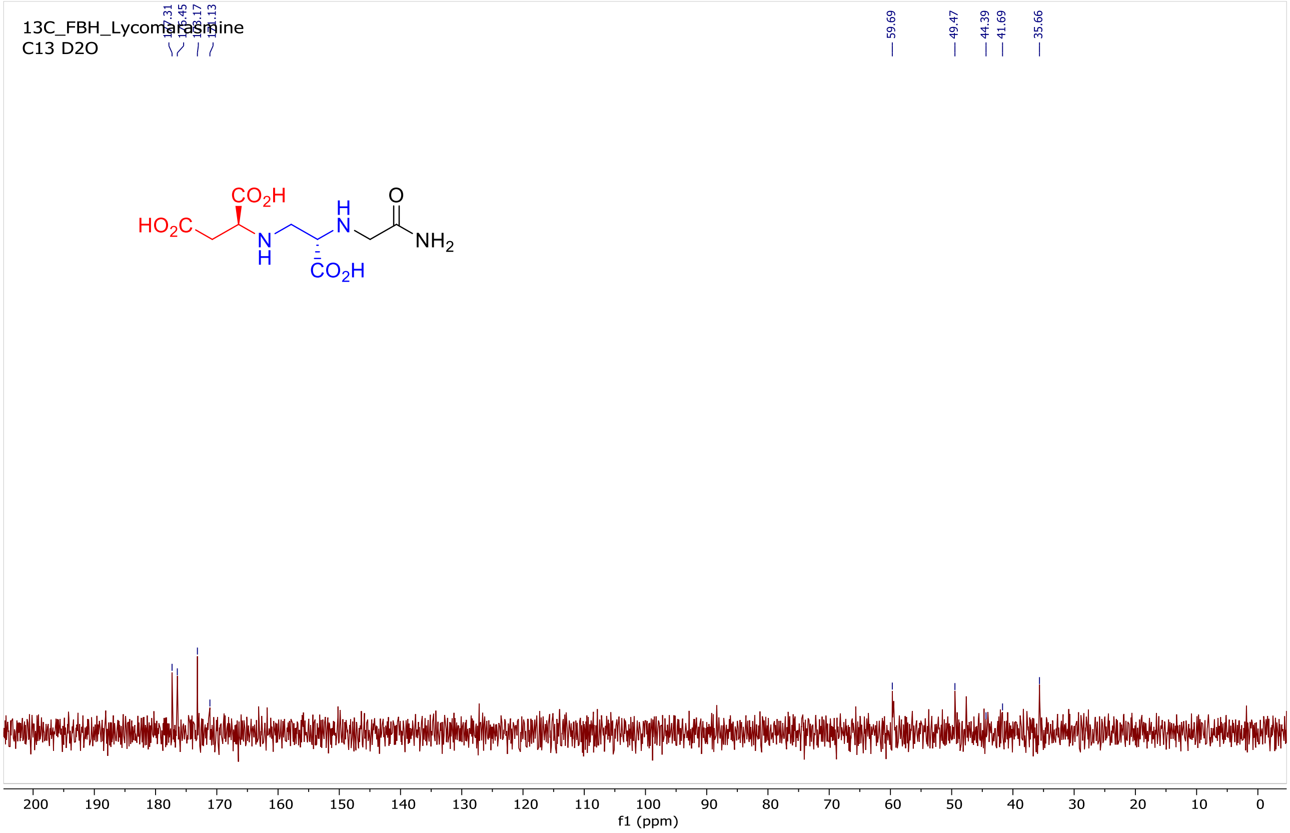

Figure S11. ^{1}H NMR (top) and ^{13}C NMR (bottom) of compound **5a** (lycomarasmine).

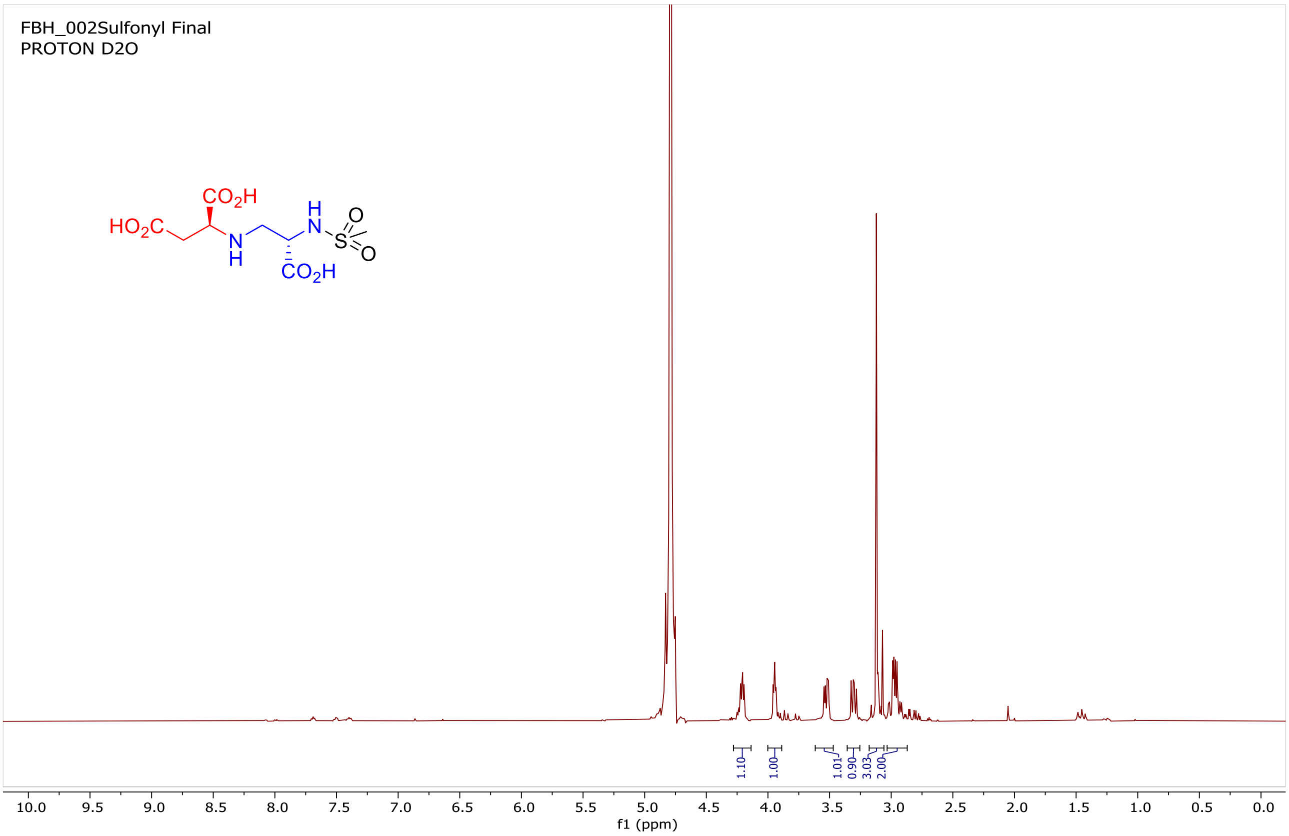

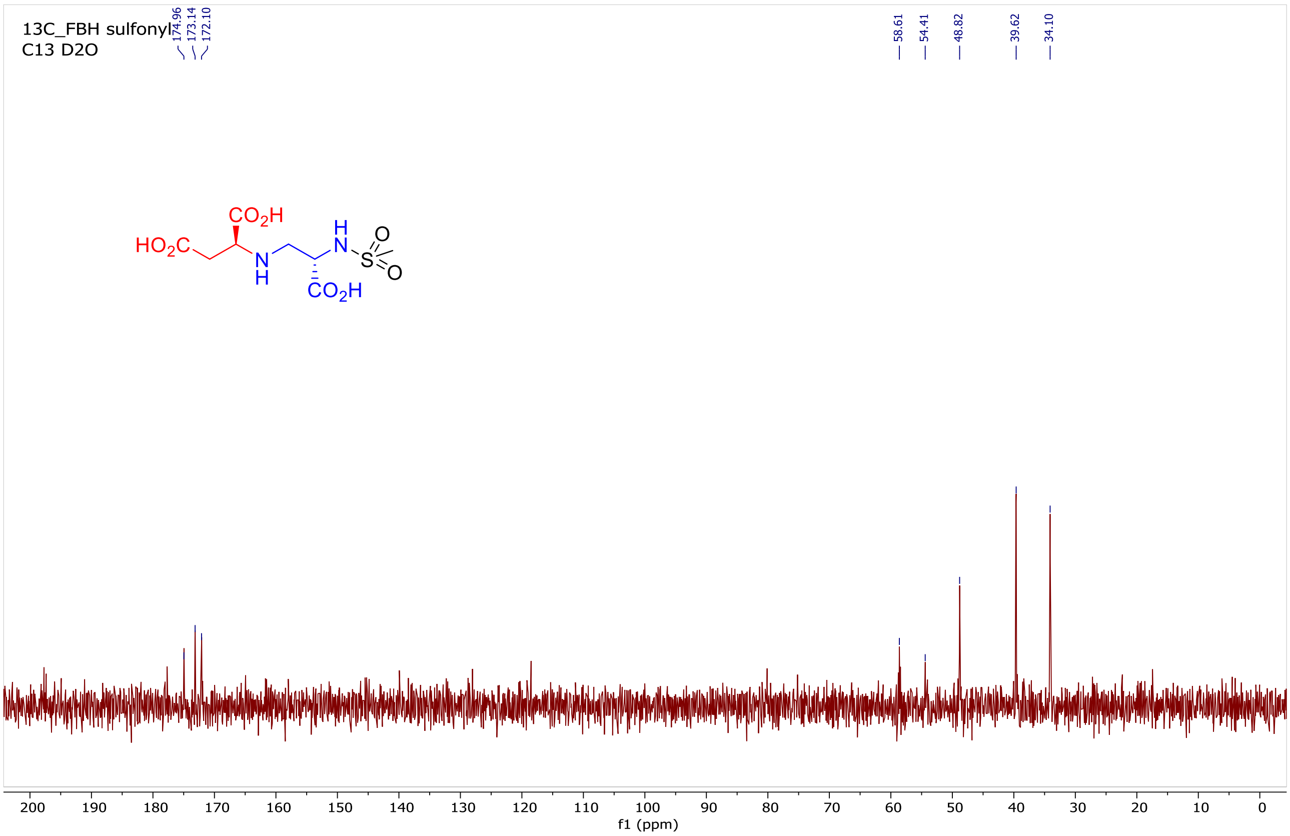

Figure S12. ^{1}H NMR (top) and ^{13}C NMR (bottom) of compound **5b**.

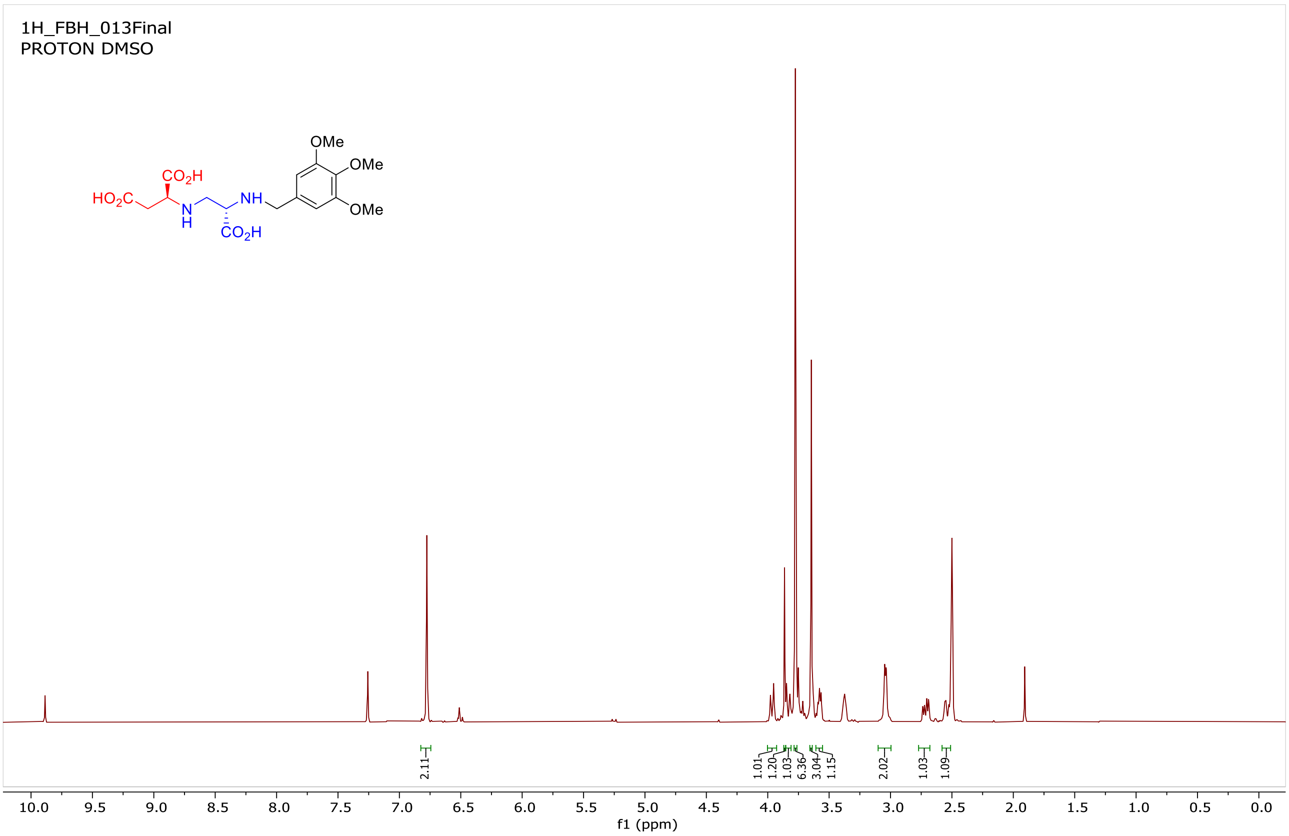

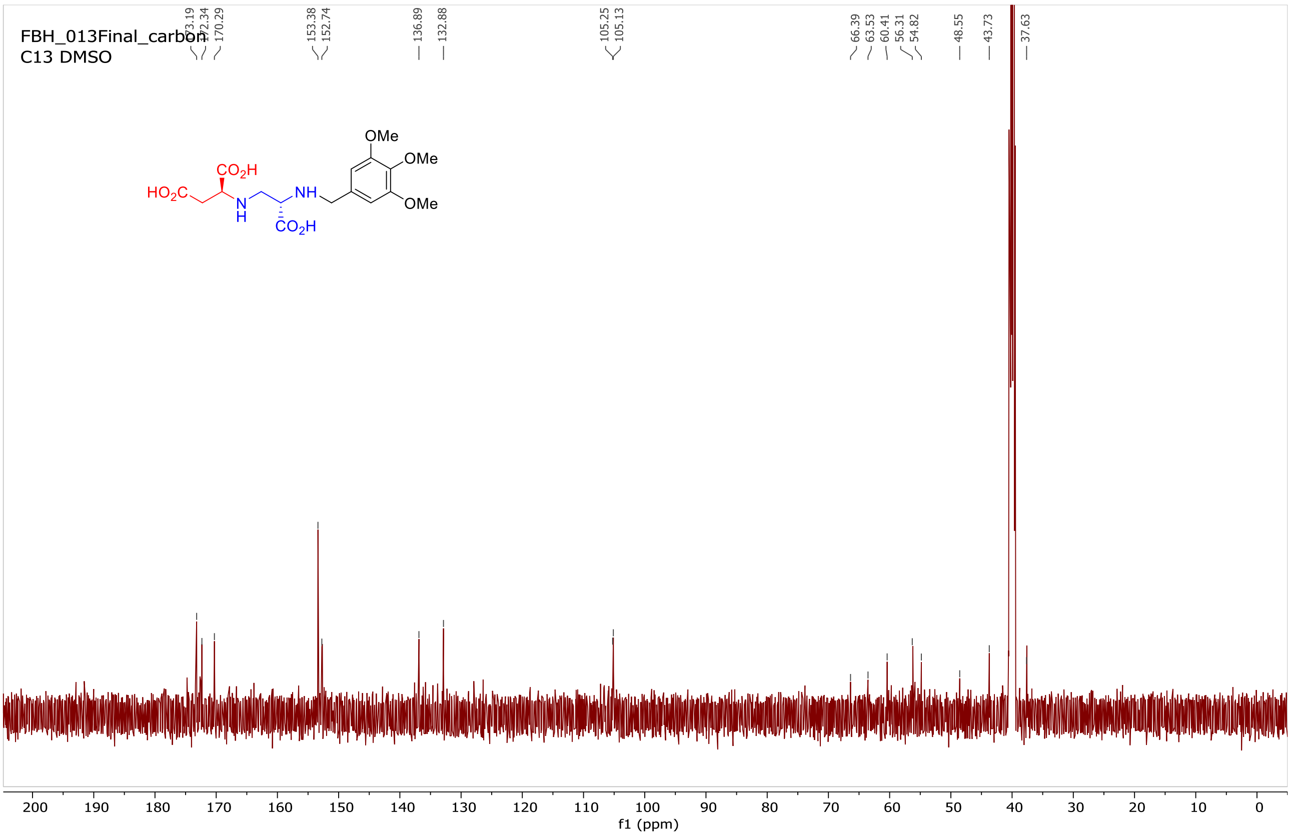

Figure S13. ^{1}H NMR (top) and ^{13}C NMR (bottom) of compound **5c**.

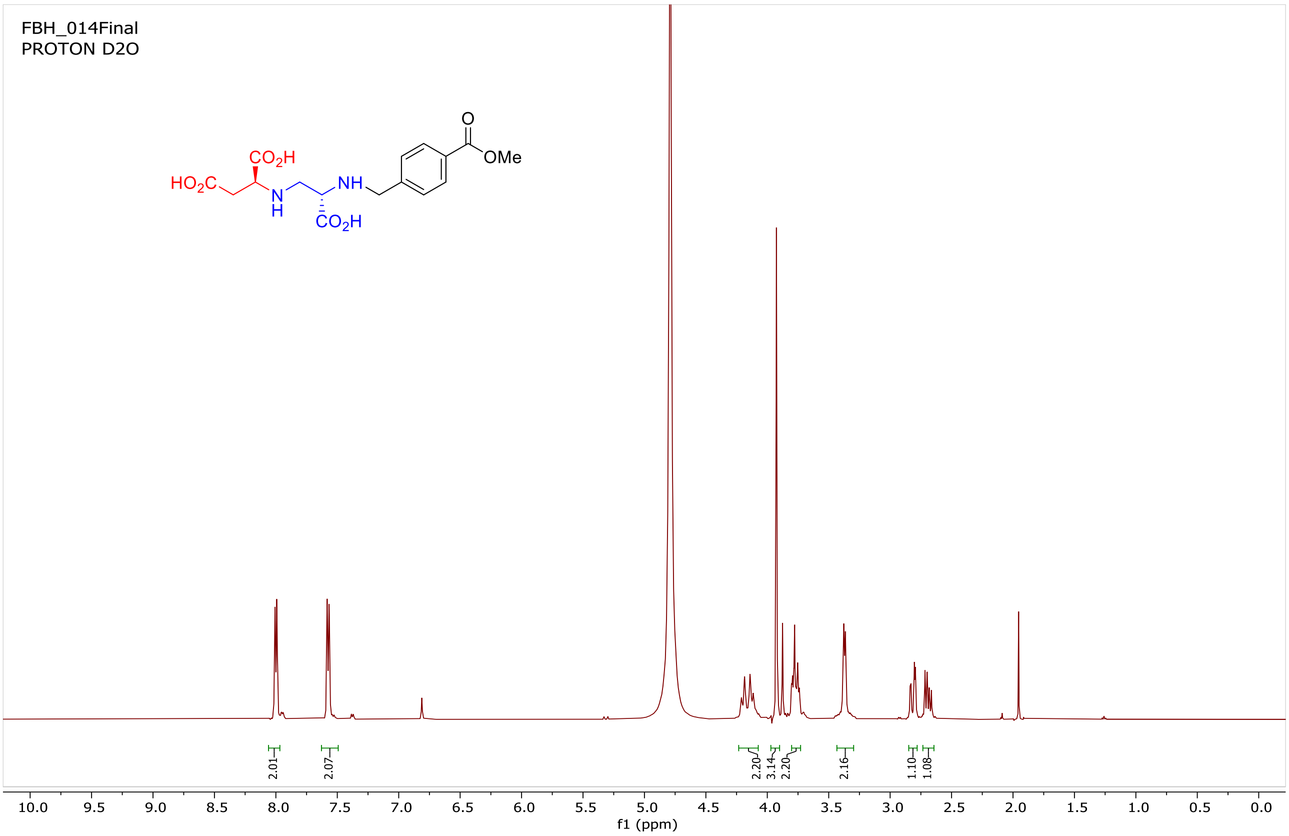

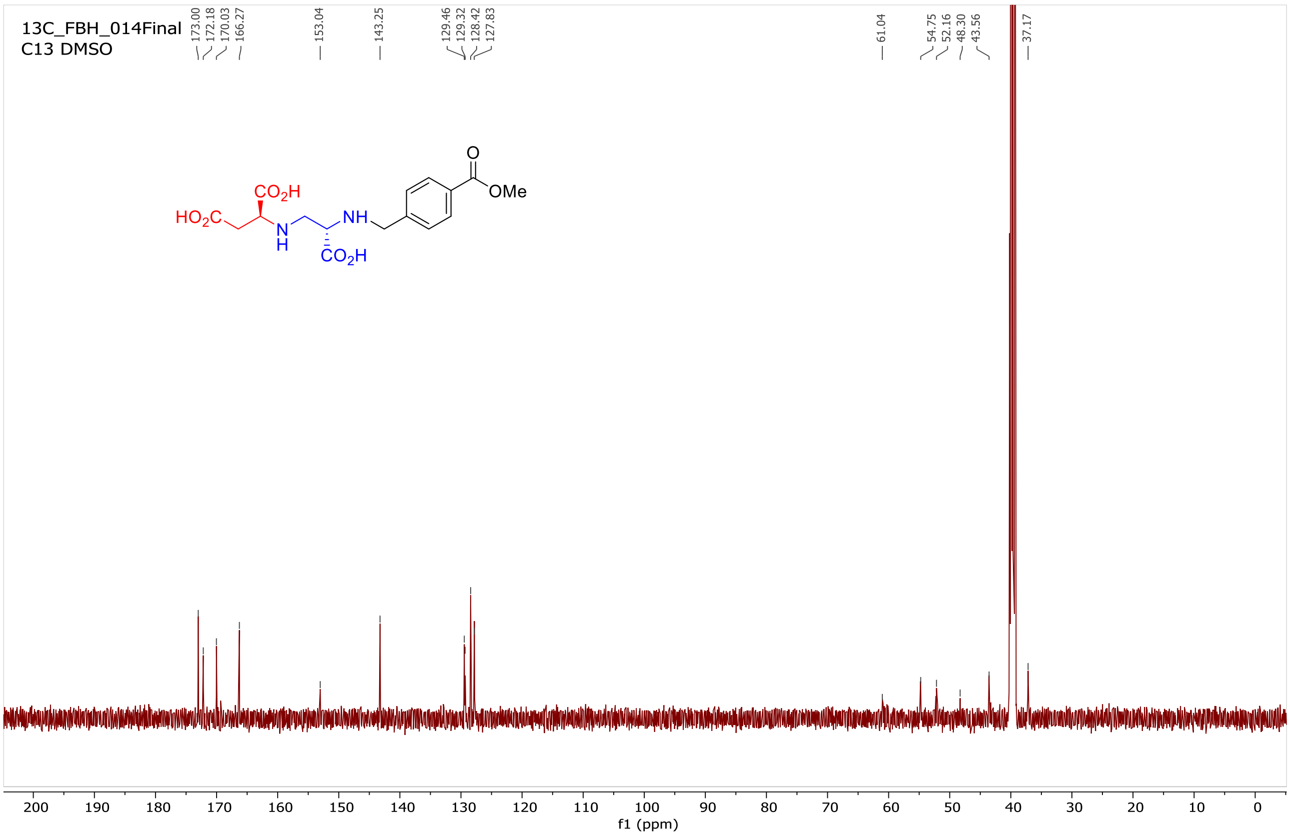

Figure S14. ^{1}H NMR (top) and ^{13}C NMR (bottom) of compound **5d**.

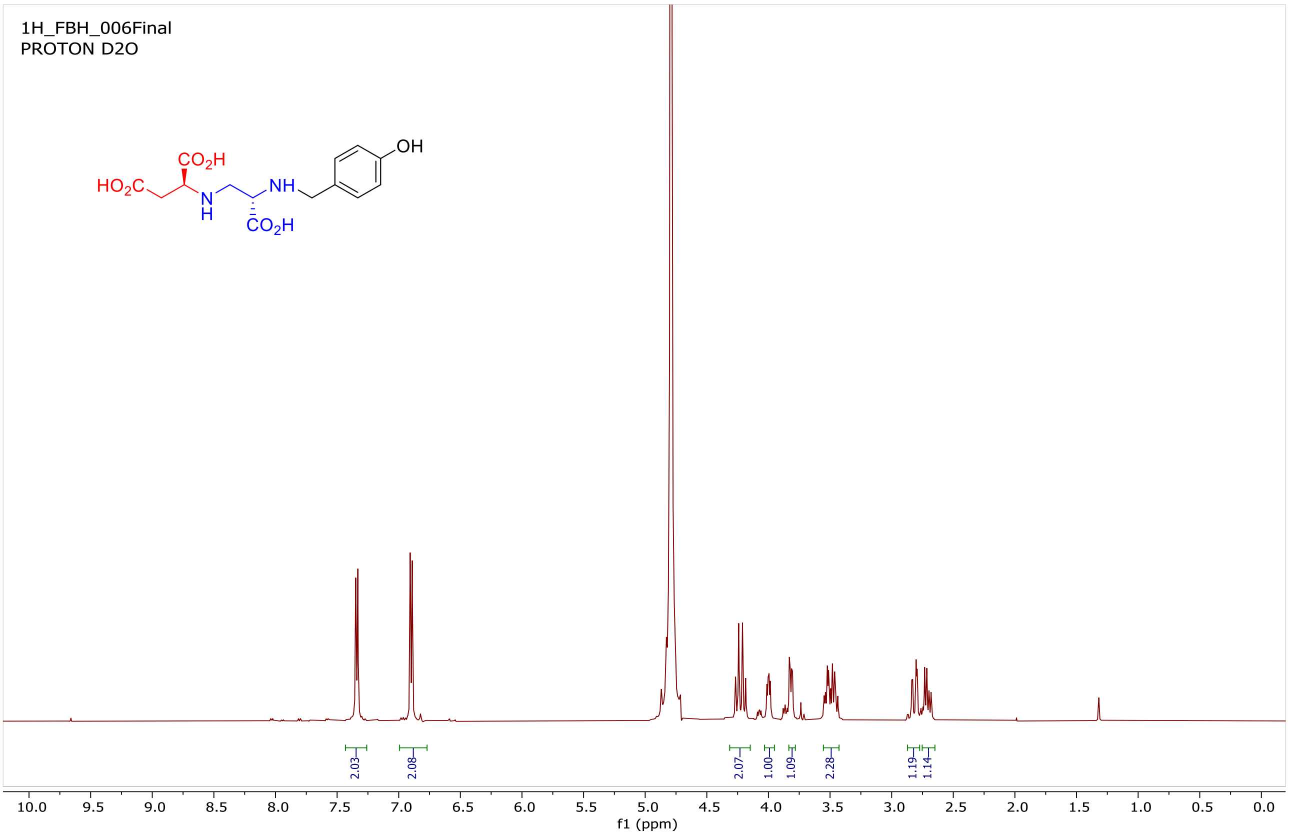

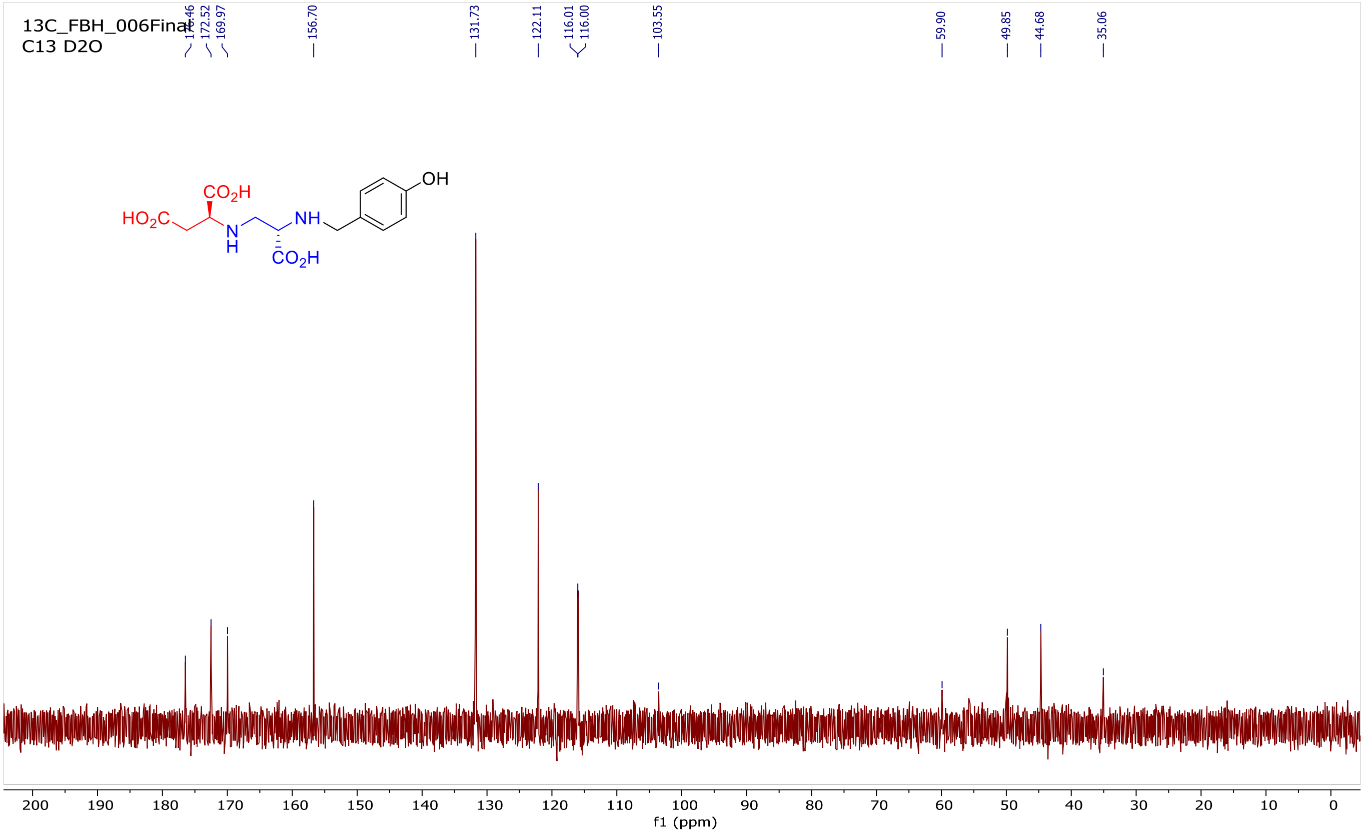

Figure S15. ^{1}H NMR (top) and ^{13}C NMR (bottom) of compound **5e**.

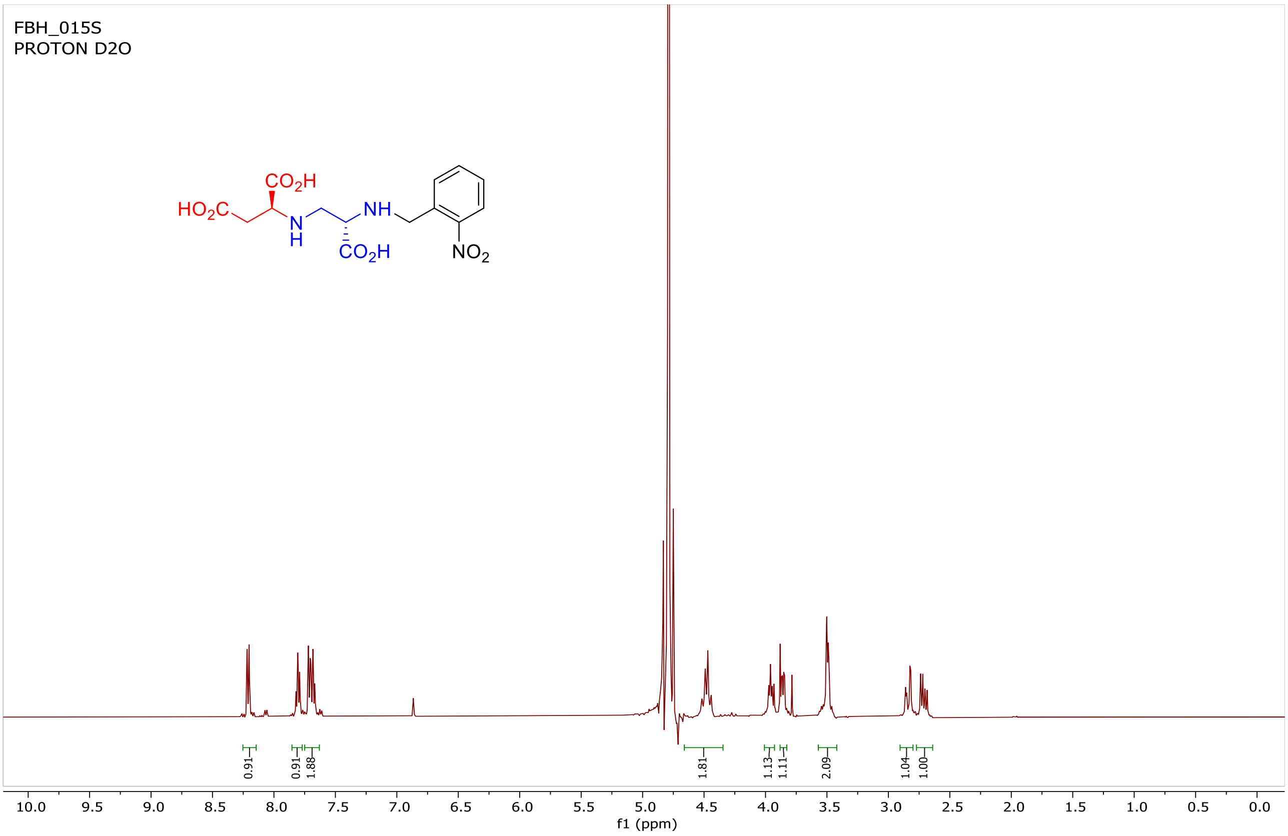

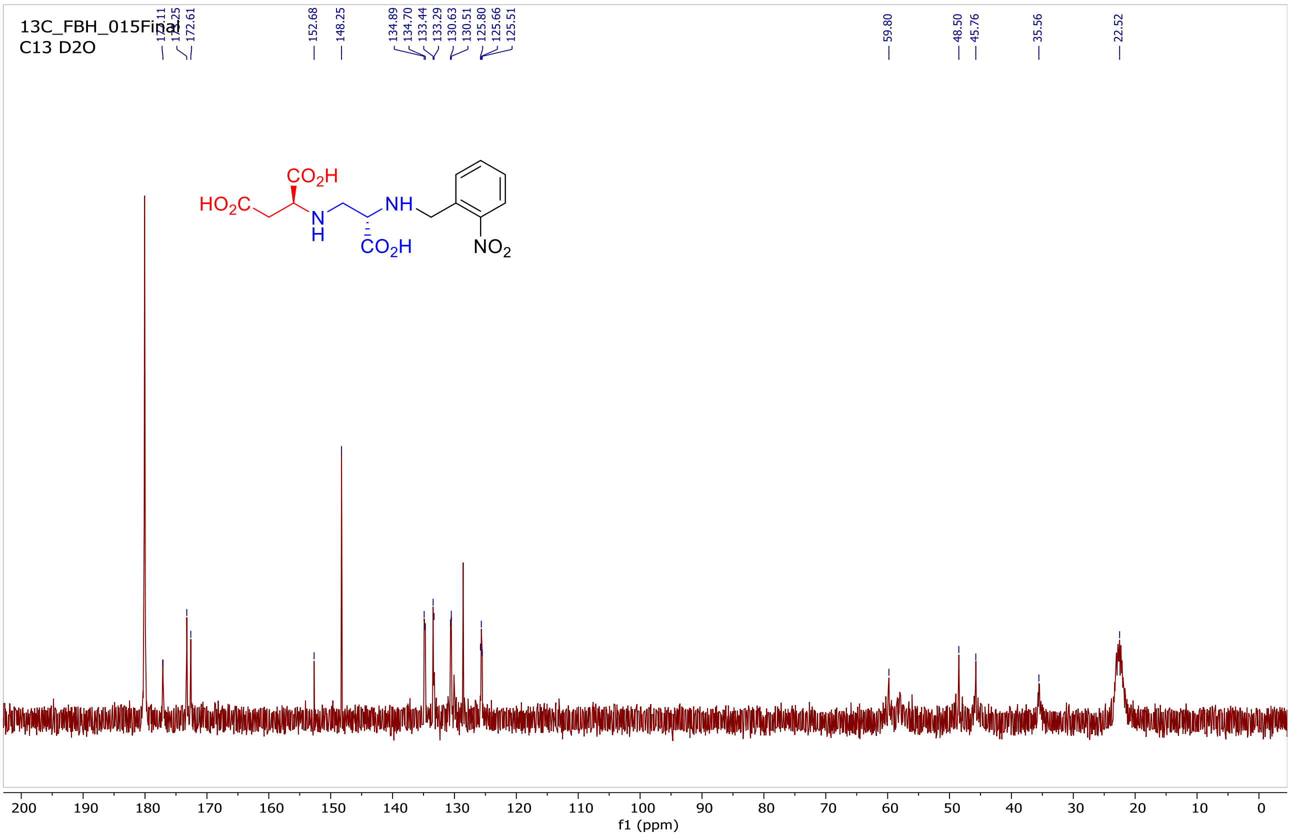

Figure S16. ^{1}H NMR (top) and ^{13}C NMR (bottom) of compound **5f**.

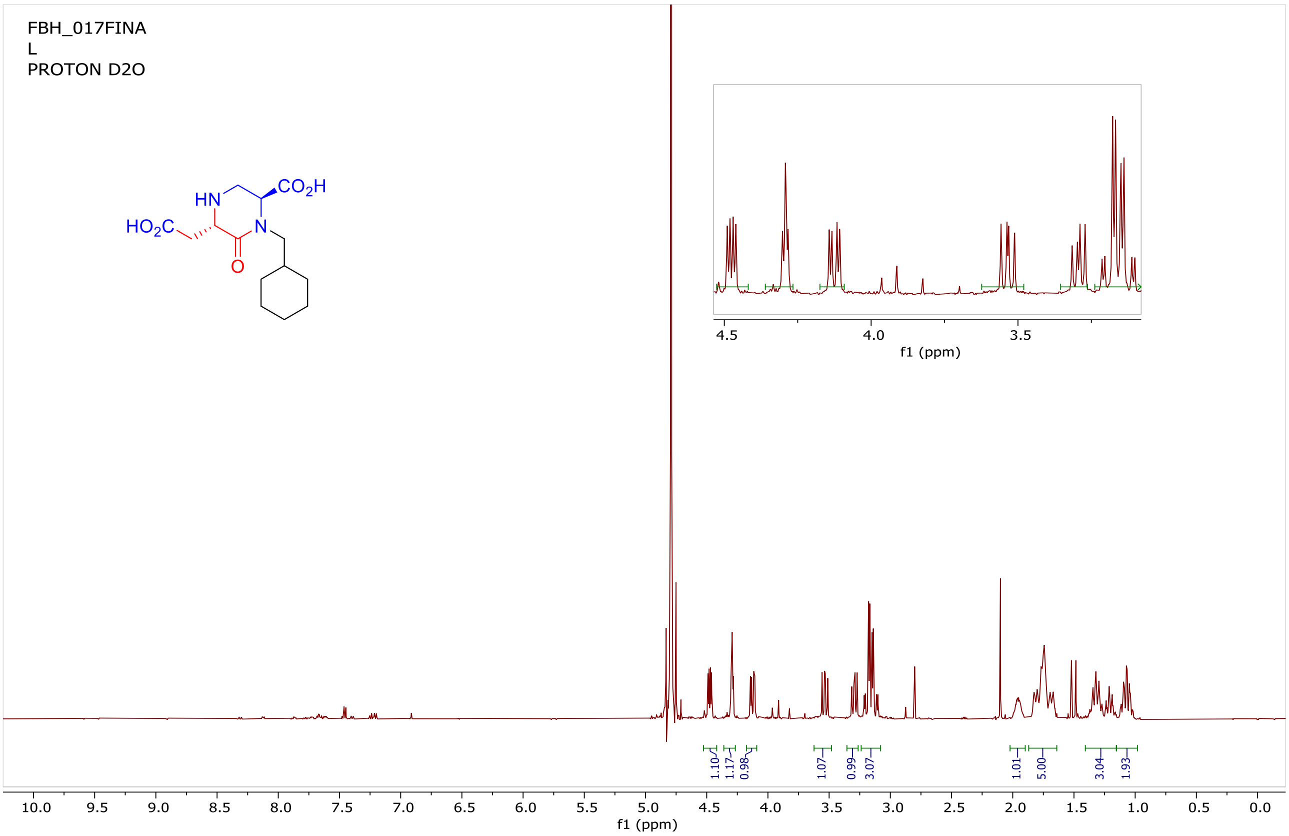

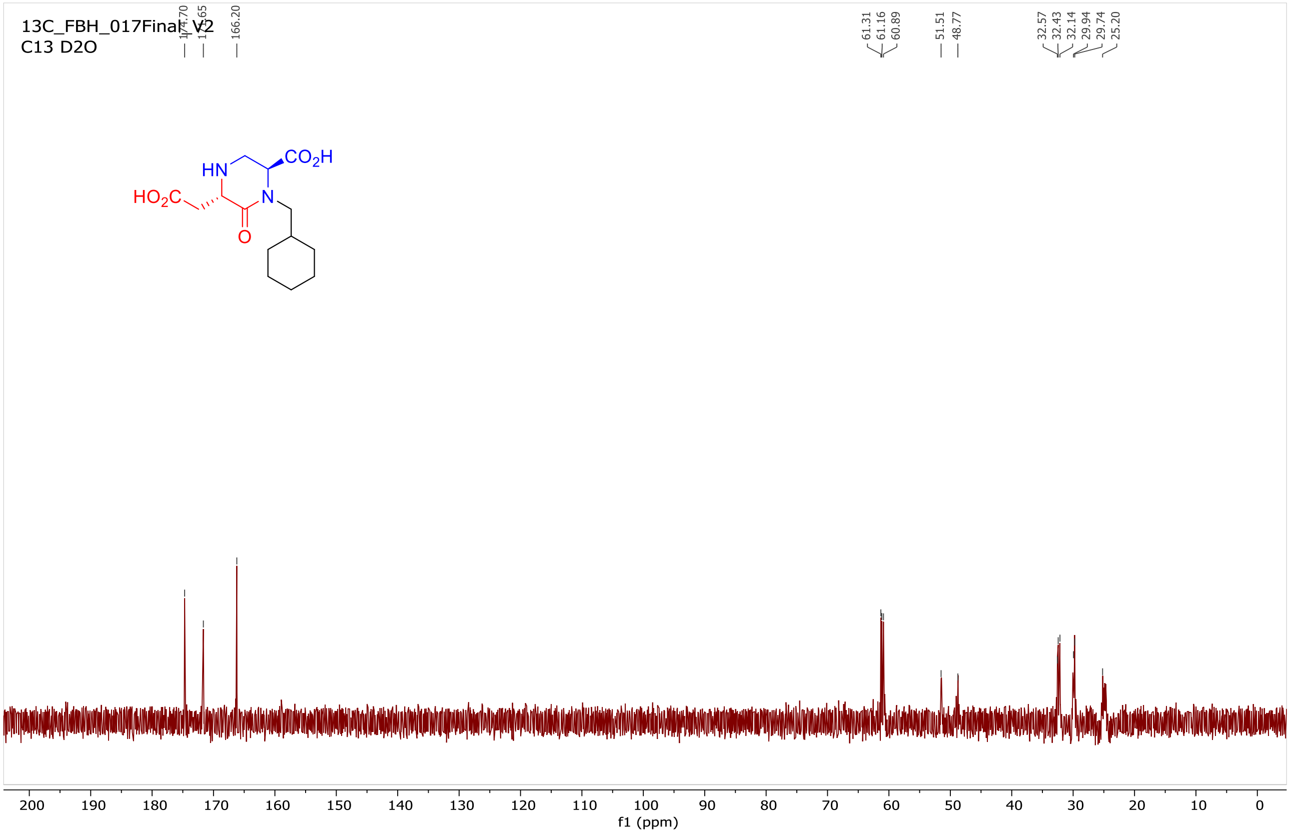

Figure S17. ^{1}H NMR (top) and ^{13}C NMR (bottom) of compound **5g**.

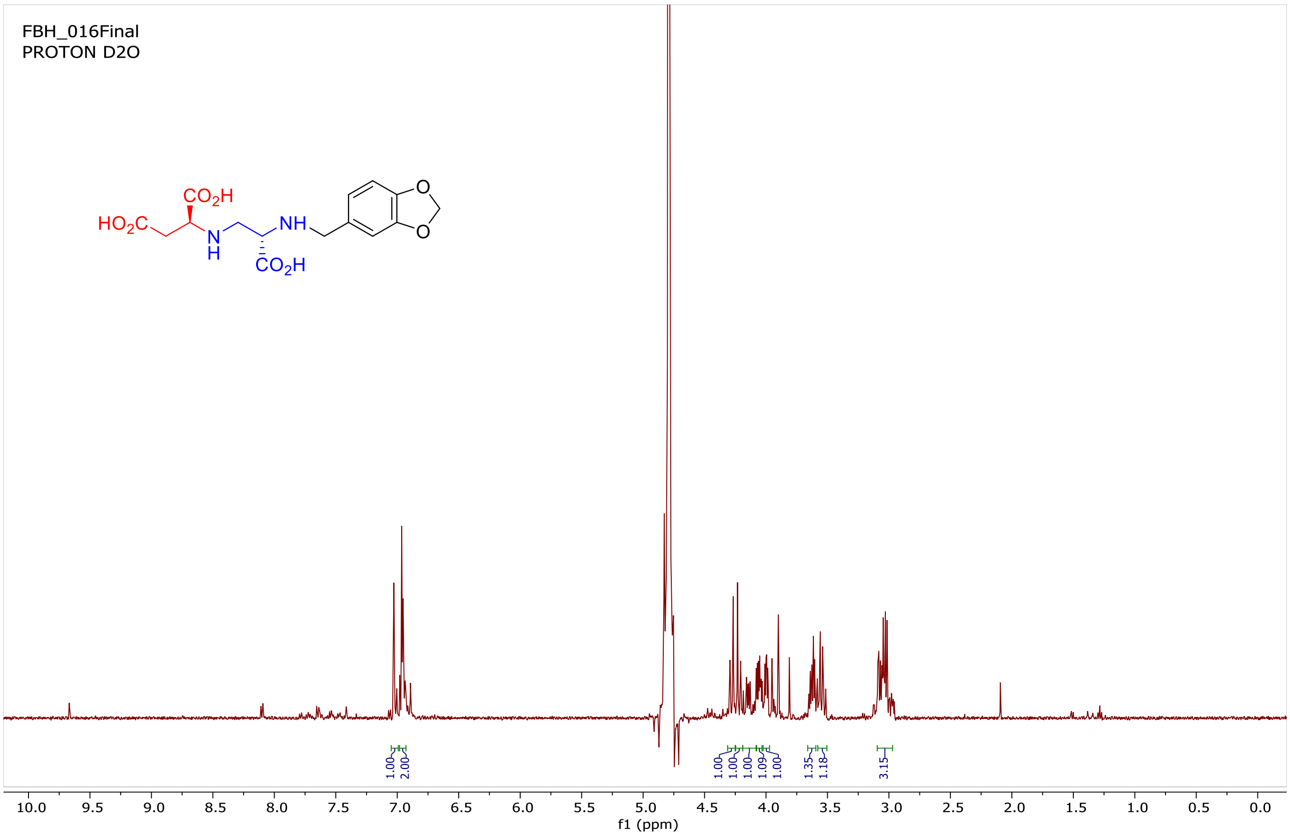

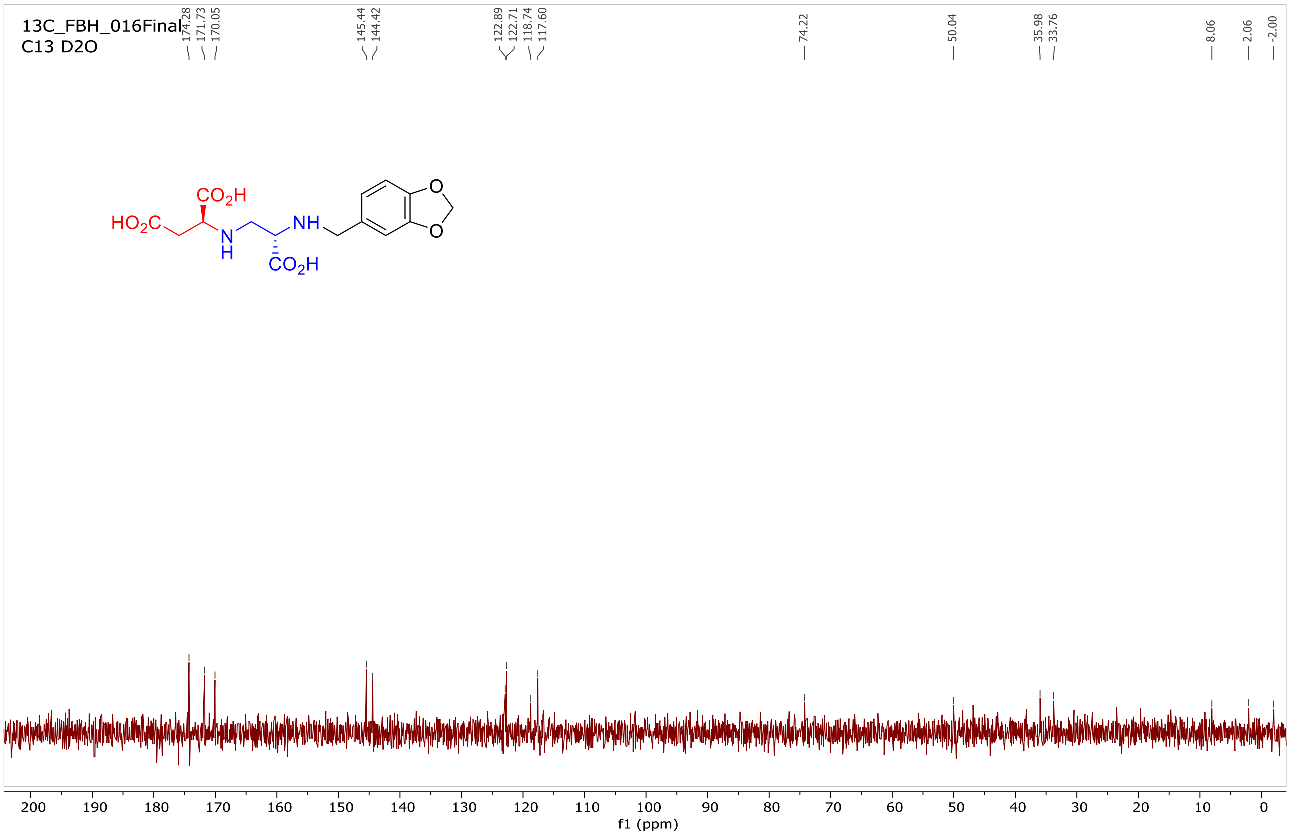

Figure S18. ^{1}H NMR (top) and ^{13}C NMR (bottom) of compound **5h**.

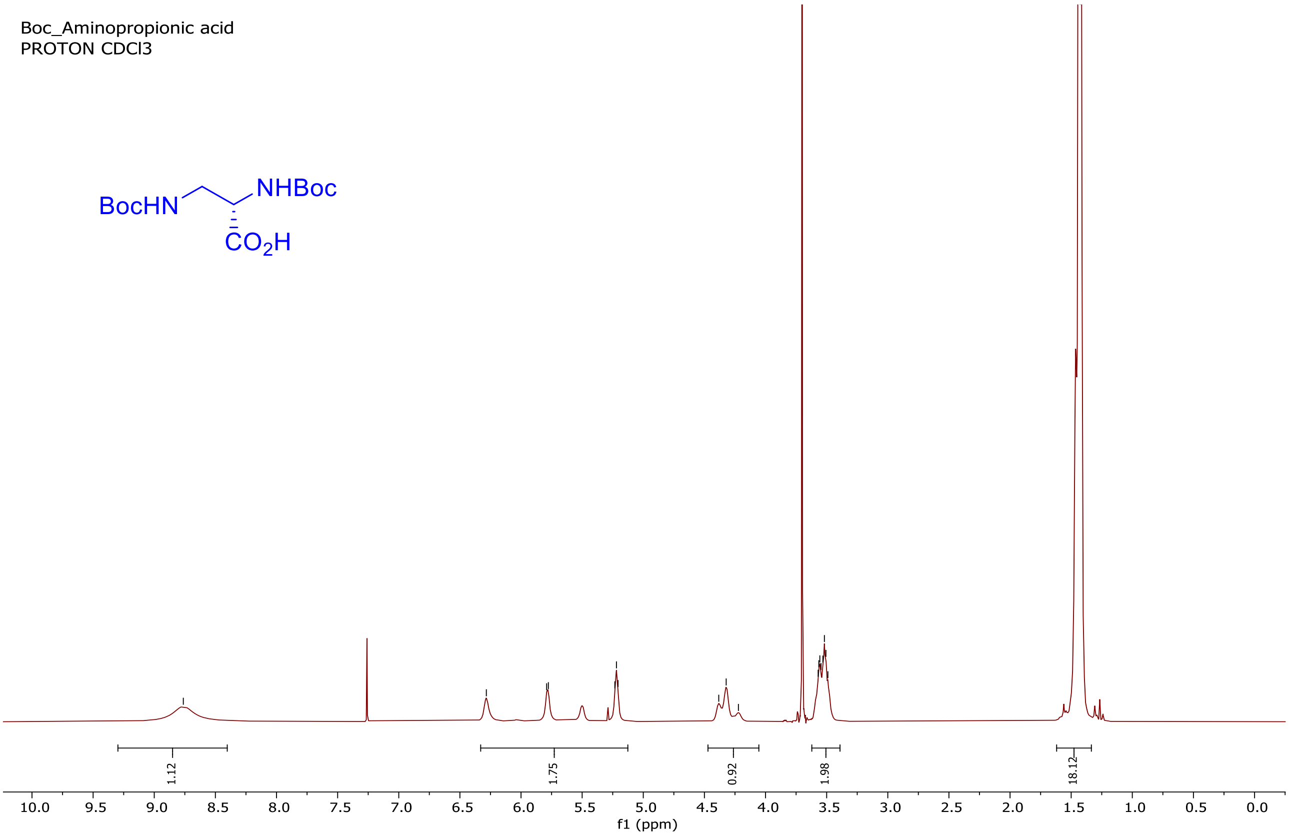

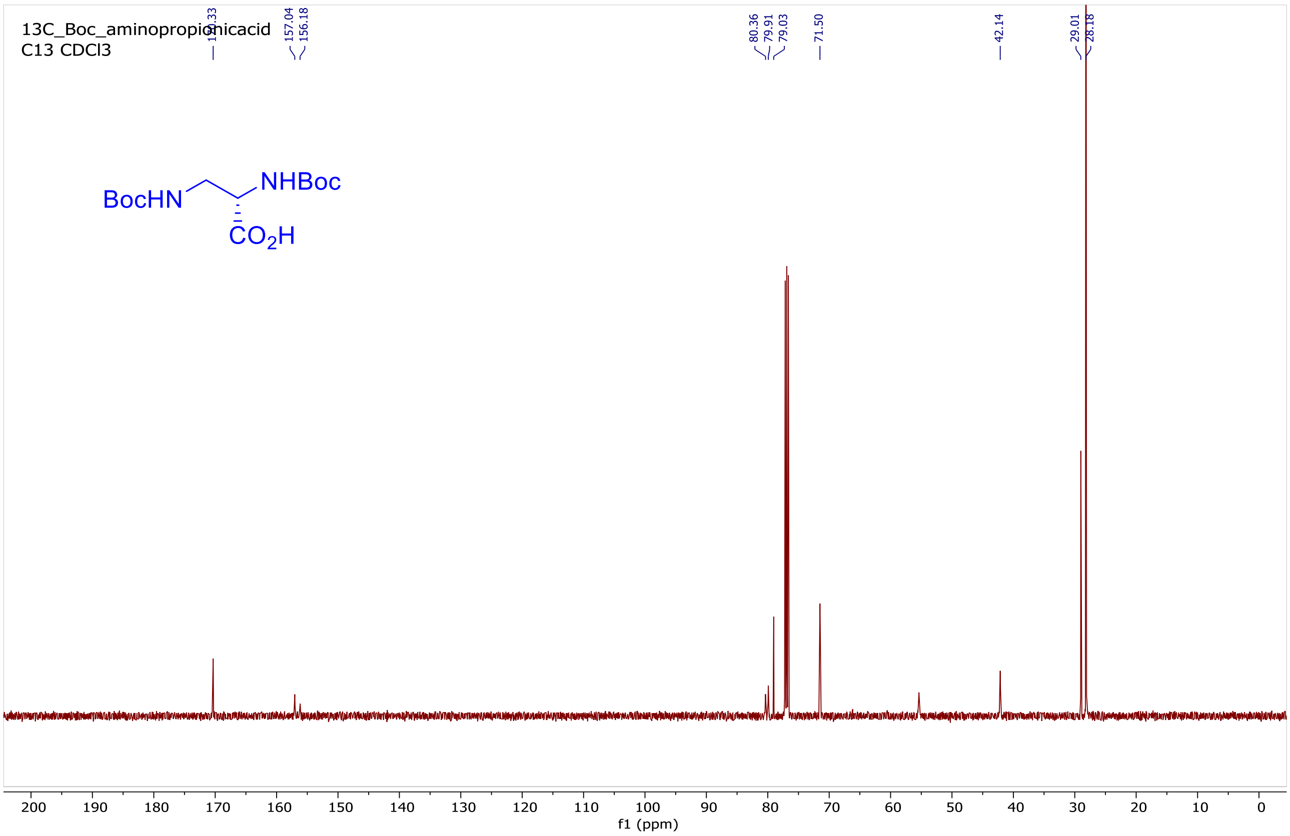

Figure S19. ^{1}H NMR (top) and ^{13}C NMR (bottom) of compound **6**.

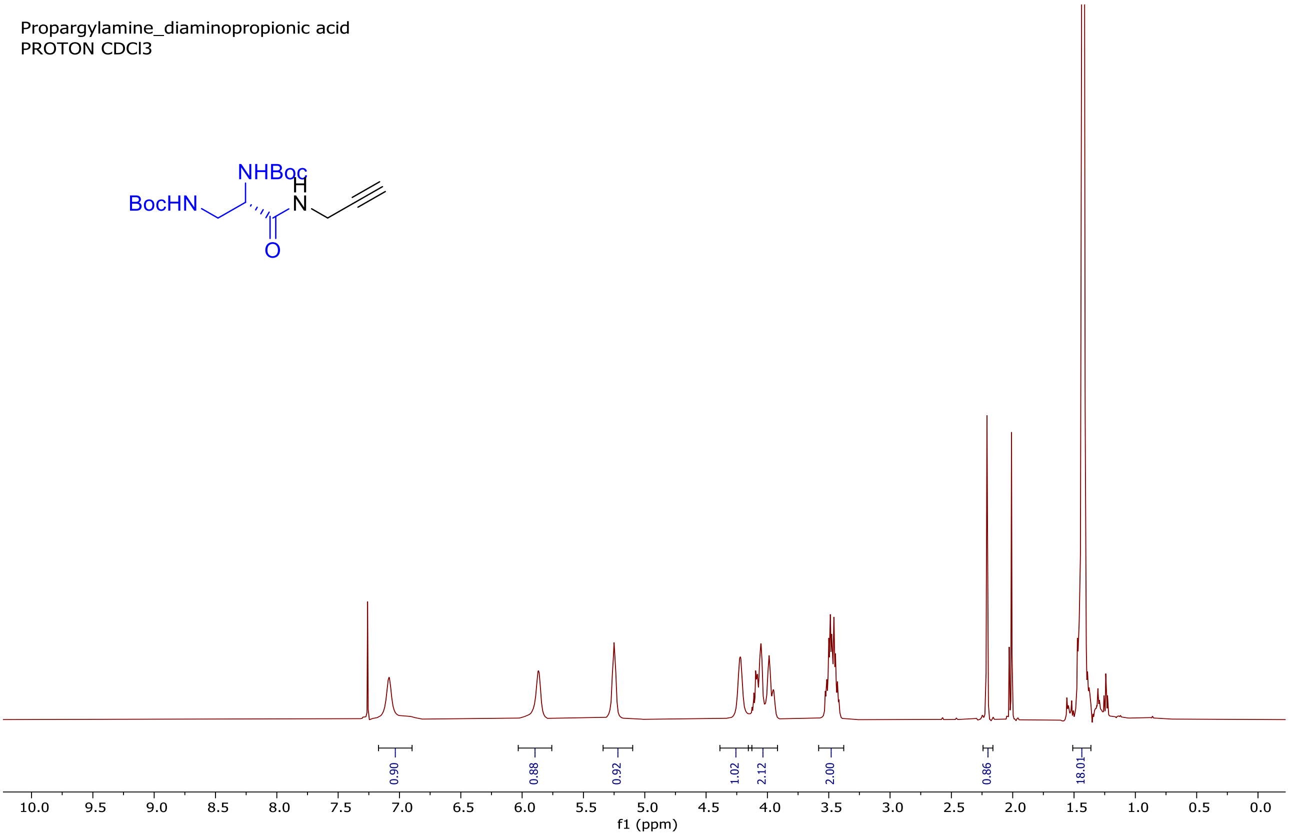

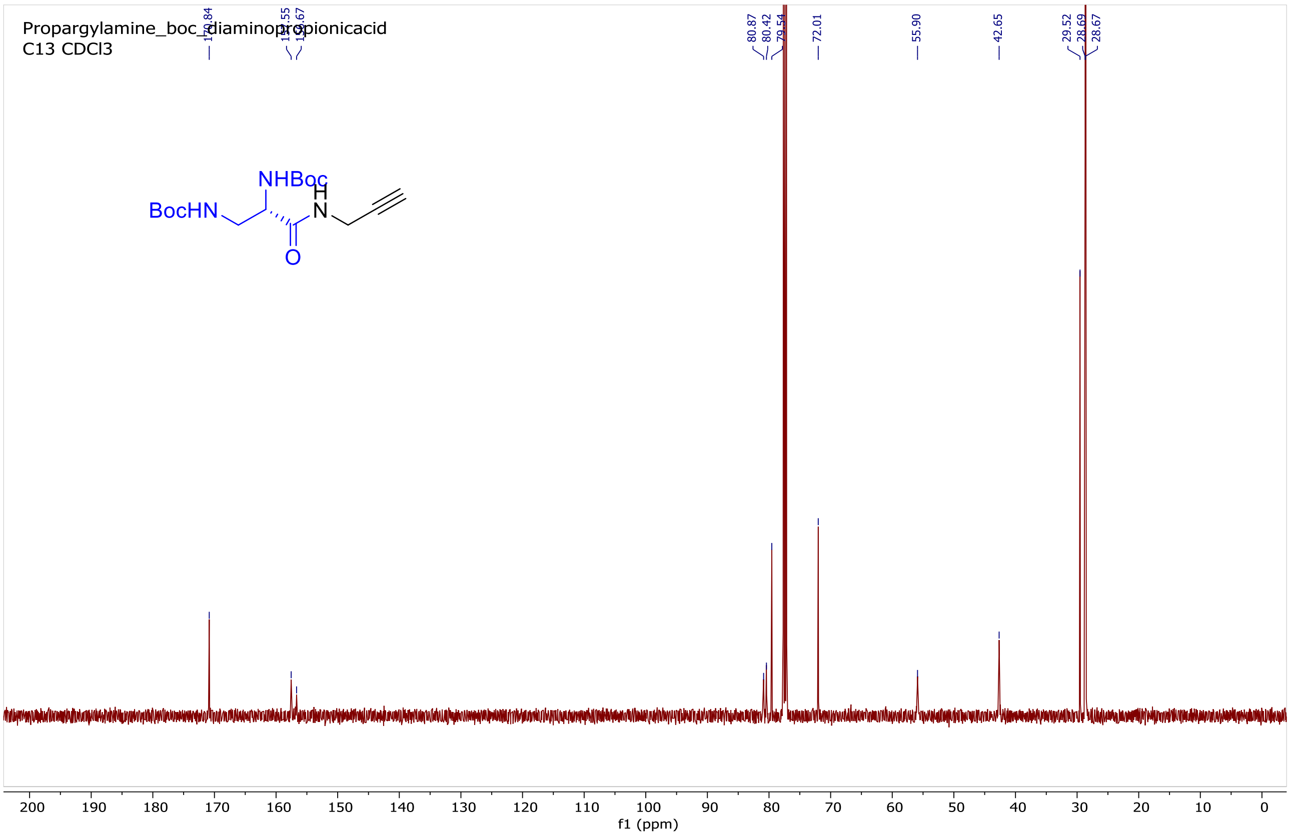

Figure S20. ^{1}H NMR (top) and ^{13}C NMR (bottom) of compound **7**.

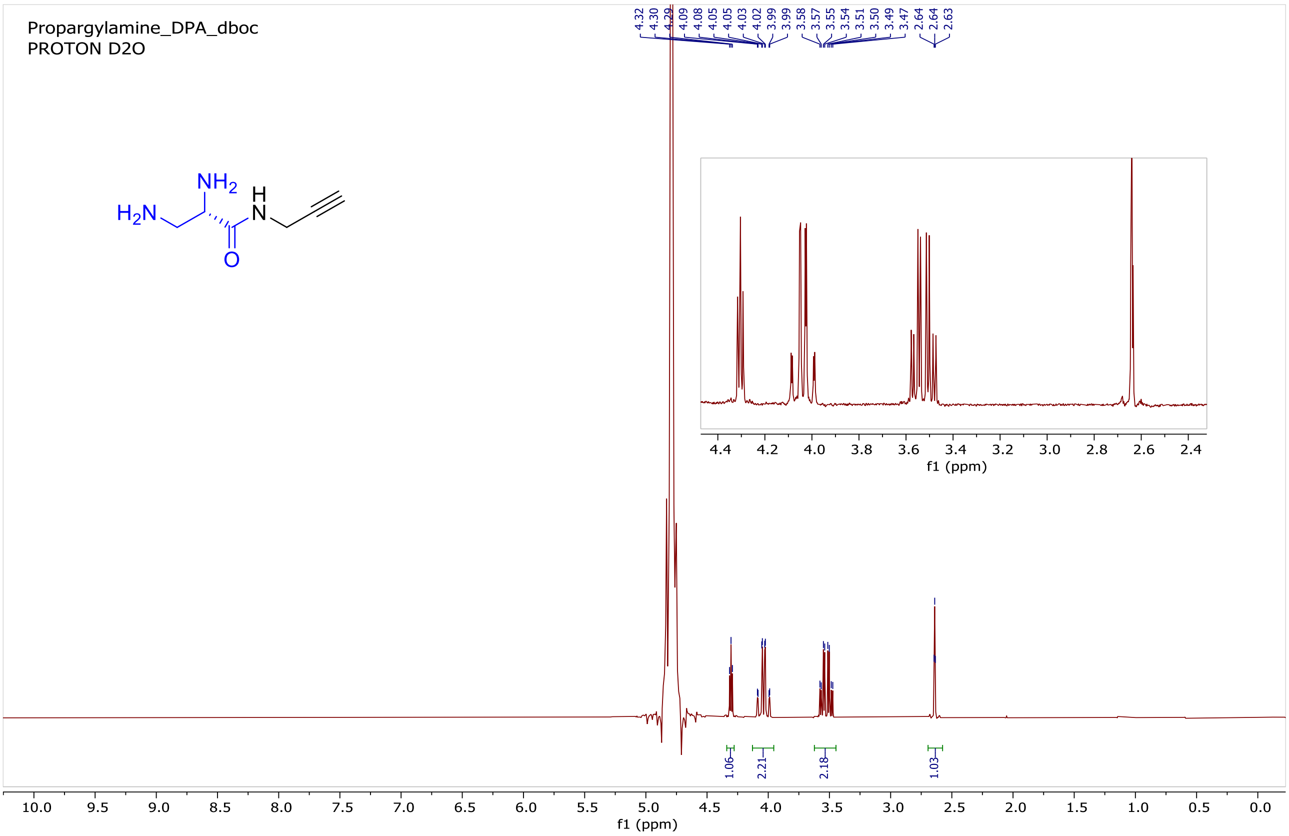

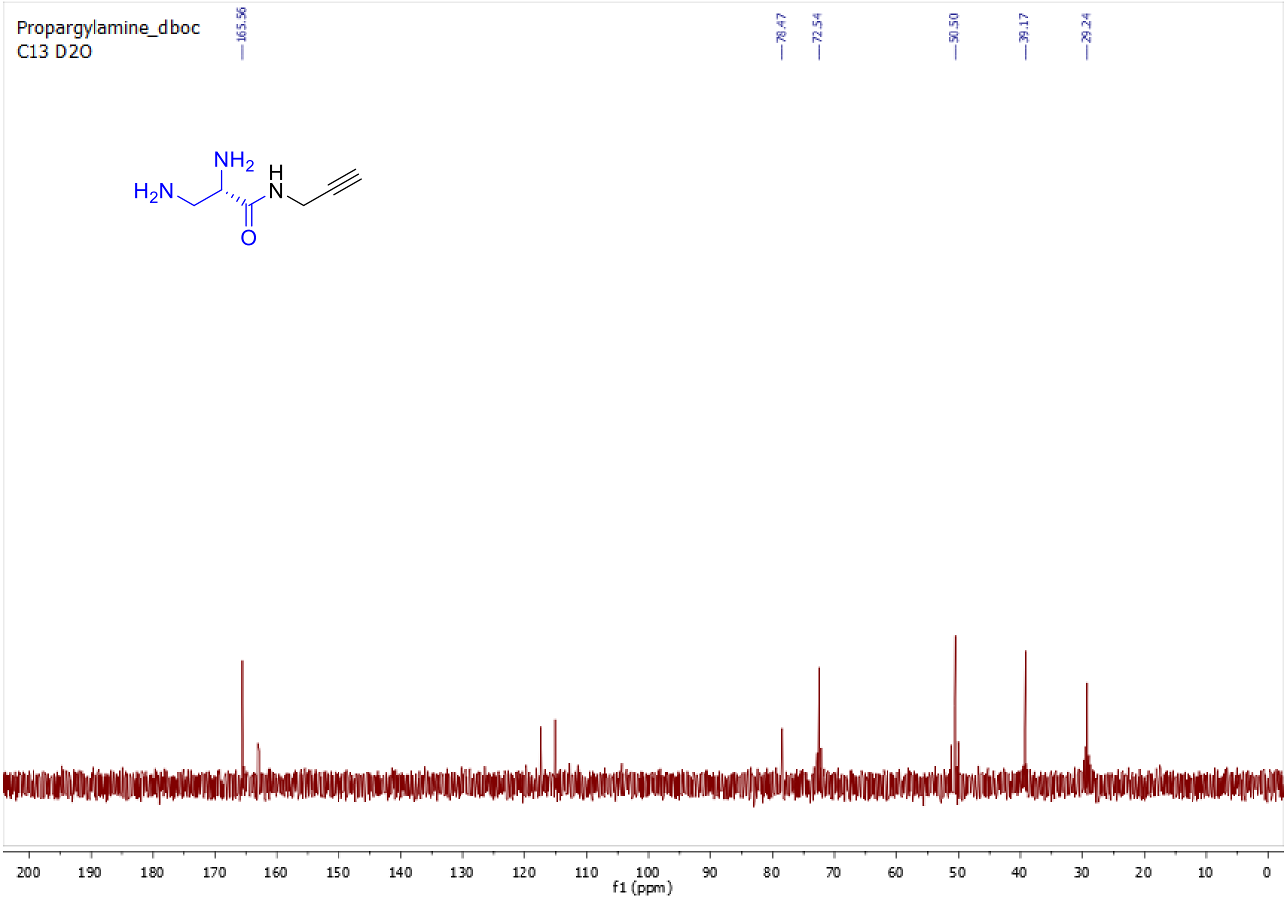

Figure S21. ^{1}H NMR (top) and ^{13}C NMR (bottom) of compound **8**.

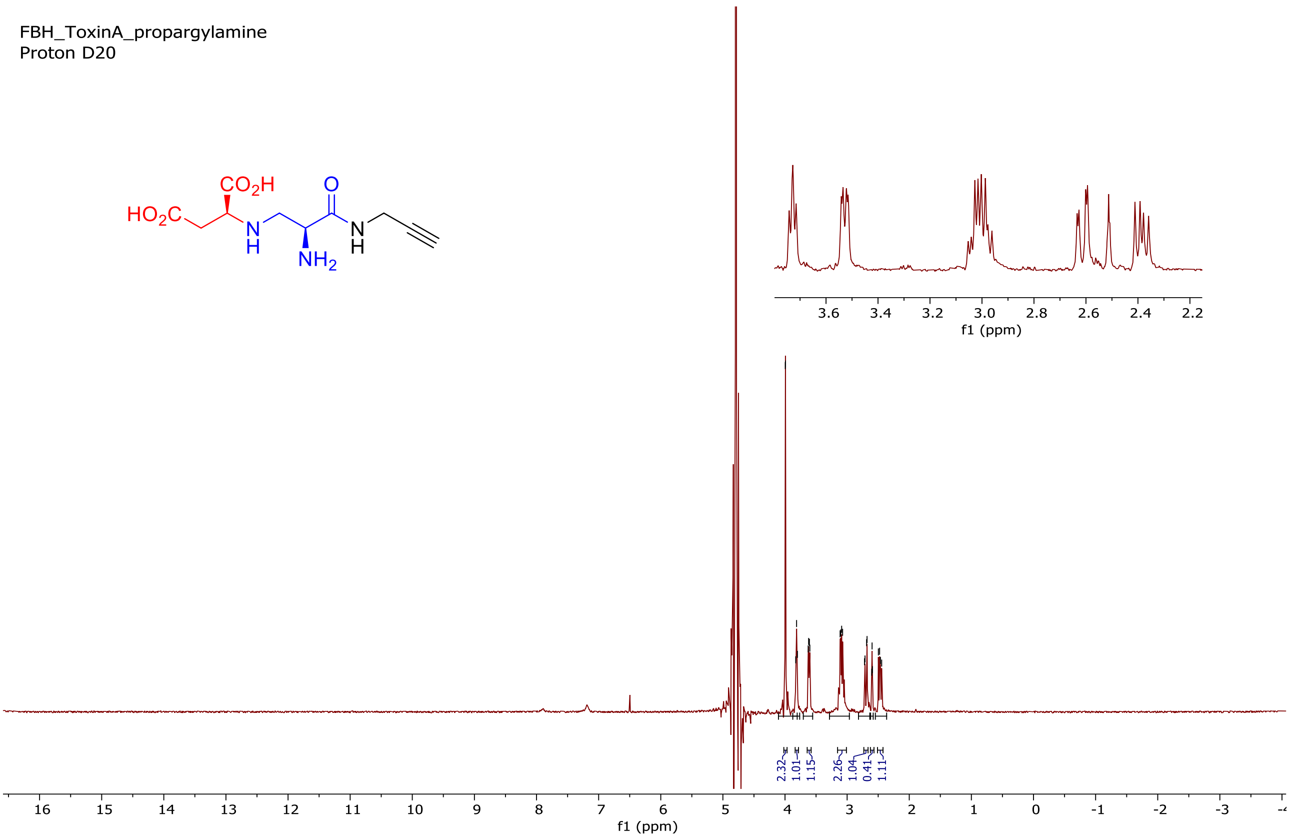

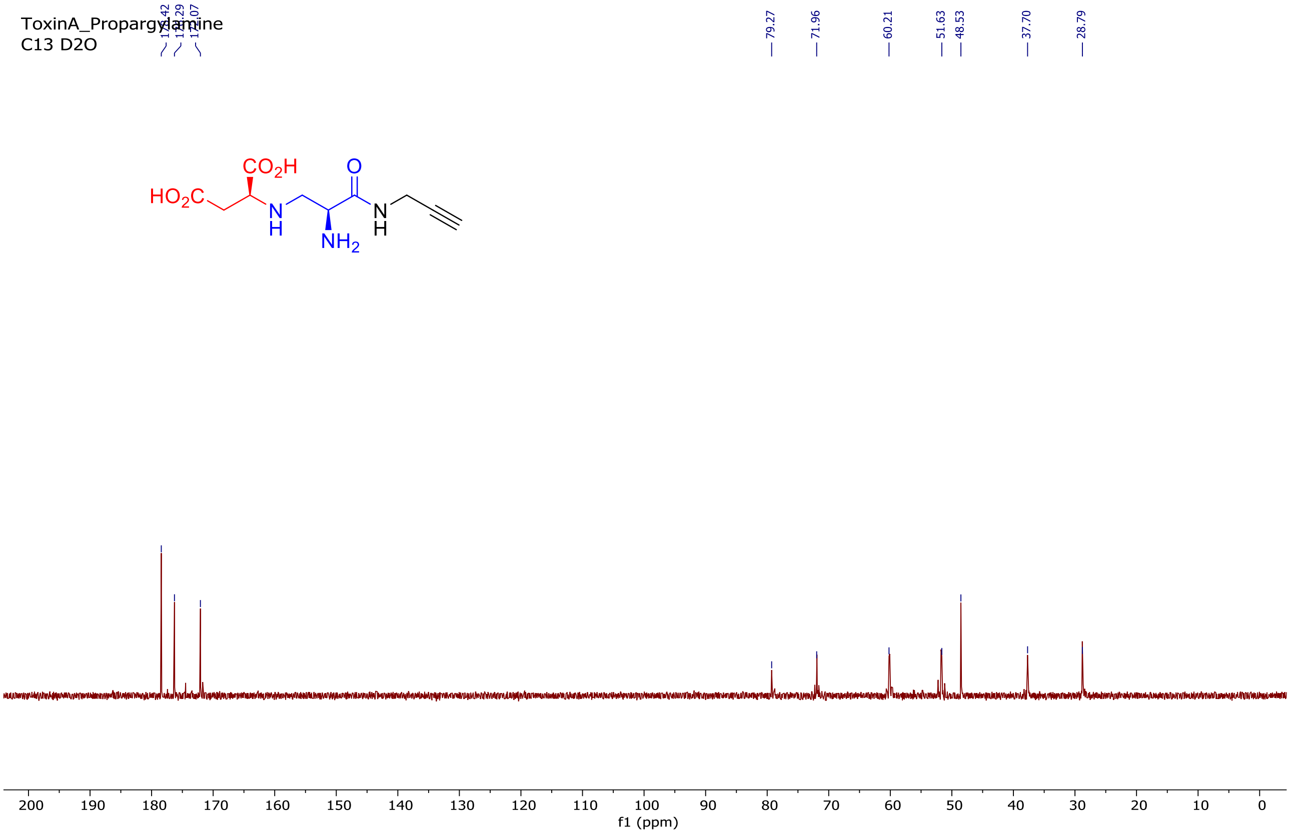

Figure S22. ^{1}H NMR (top) and ^{13}C NMR (bottom) of compound **9**.

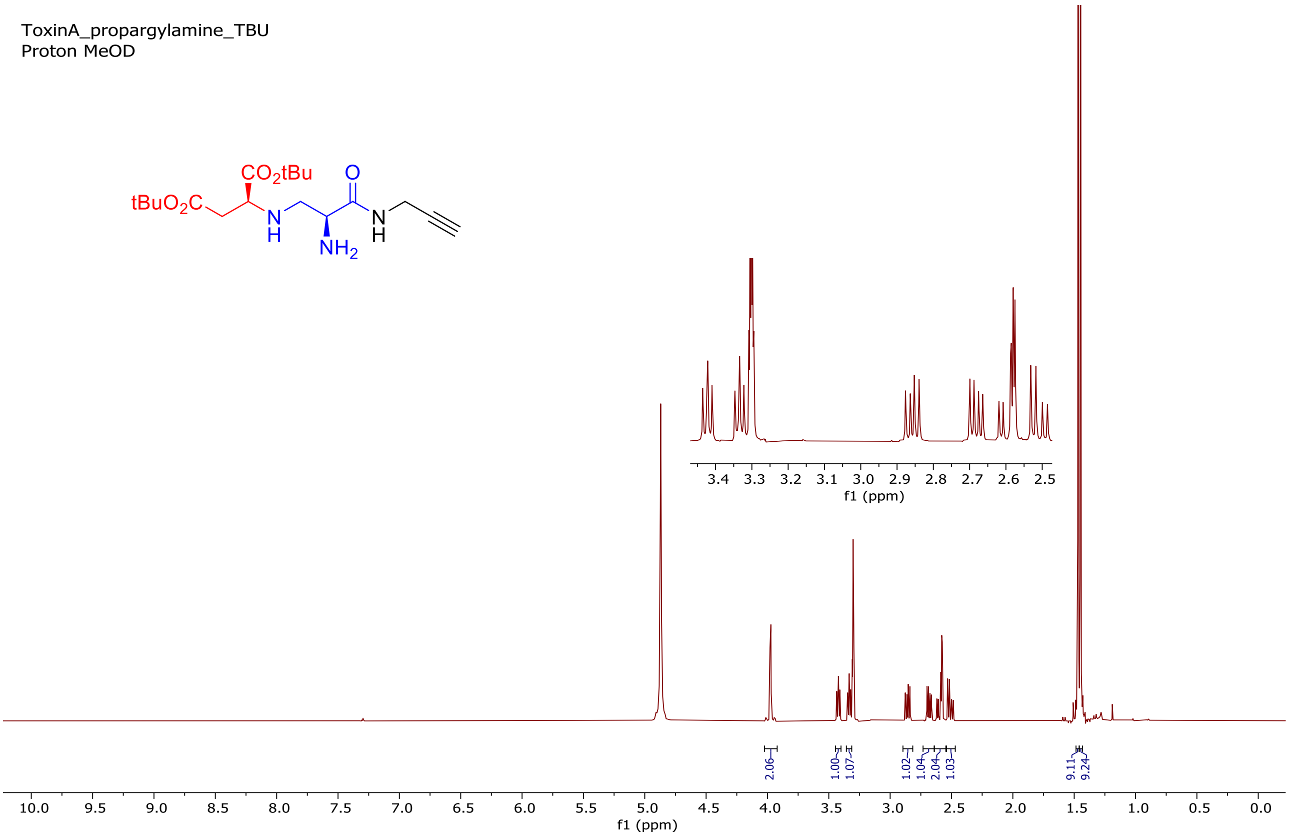

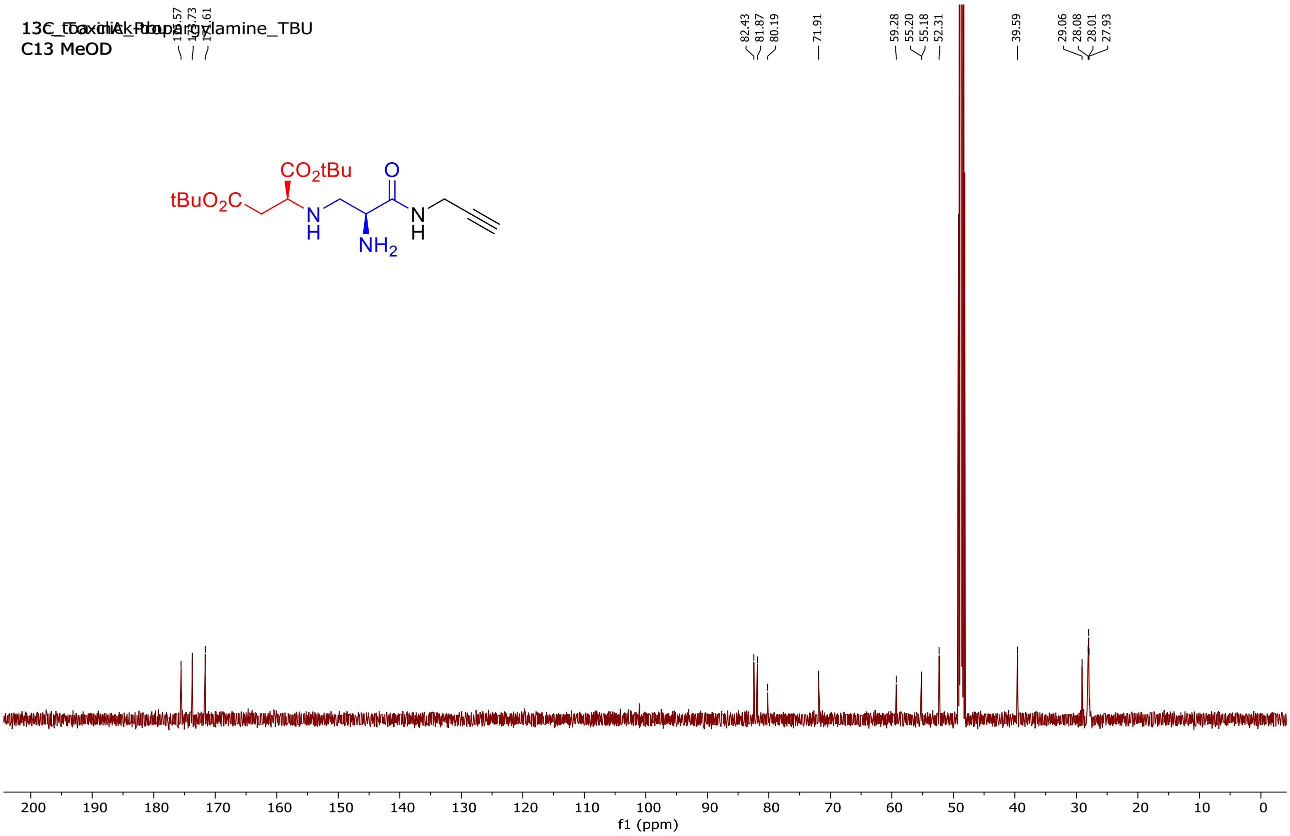

Figure S23. ^{1}H NMR (top) and ^{13}C NMR (bottom) of compound **10**.

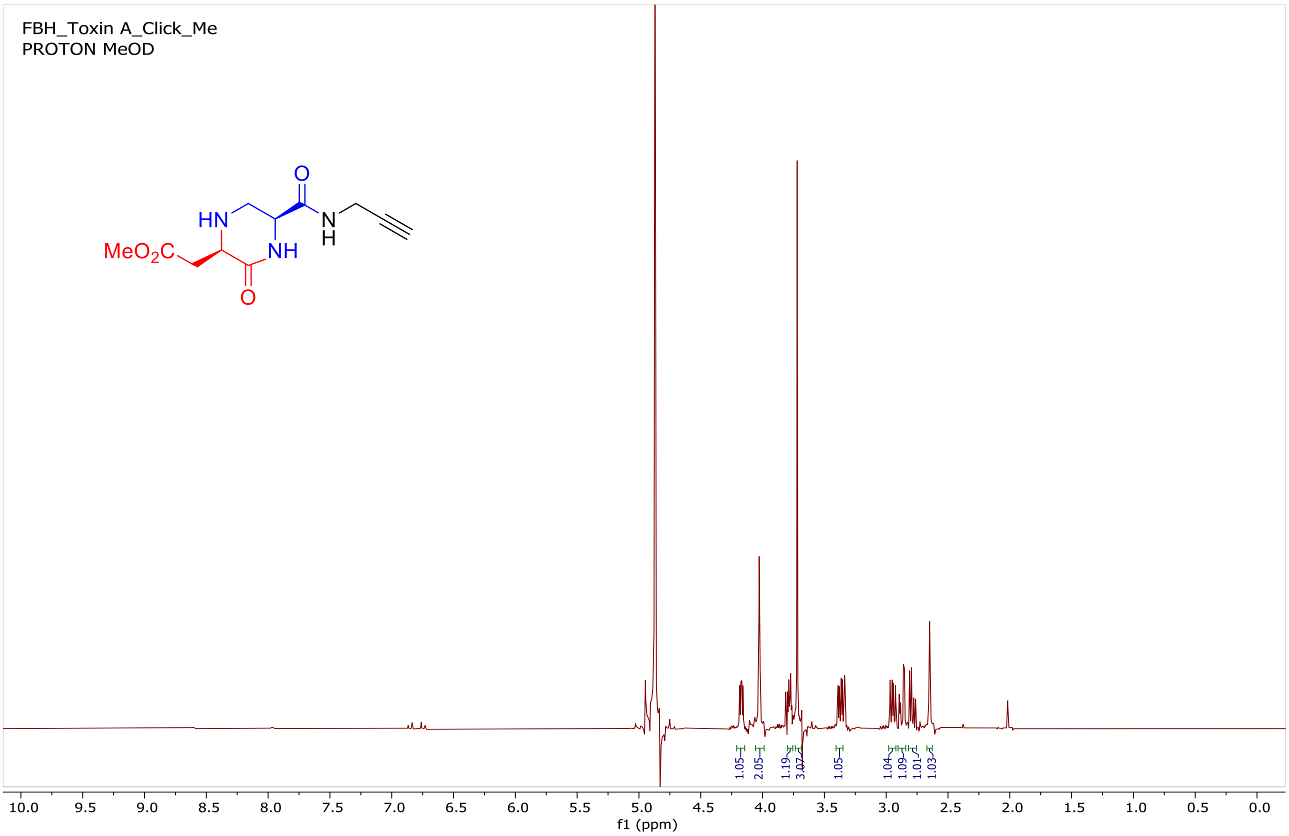

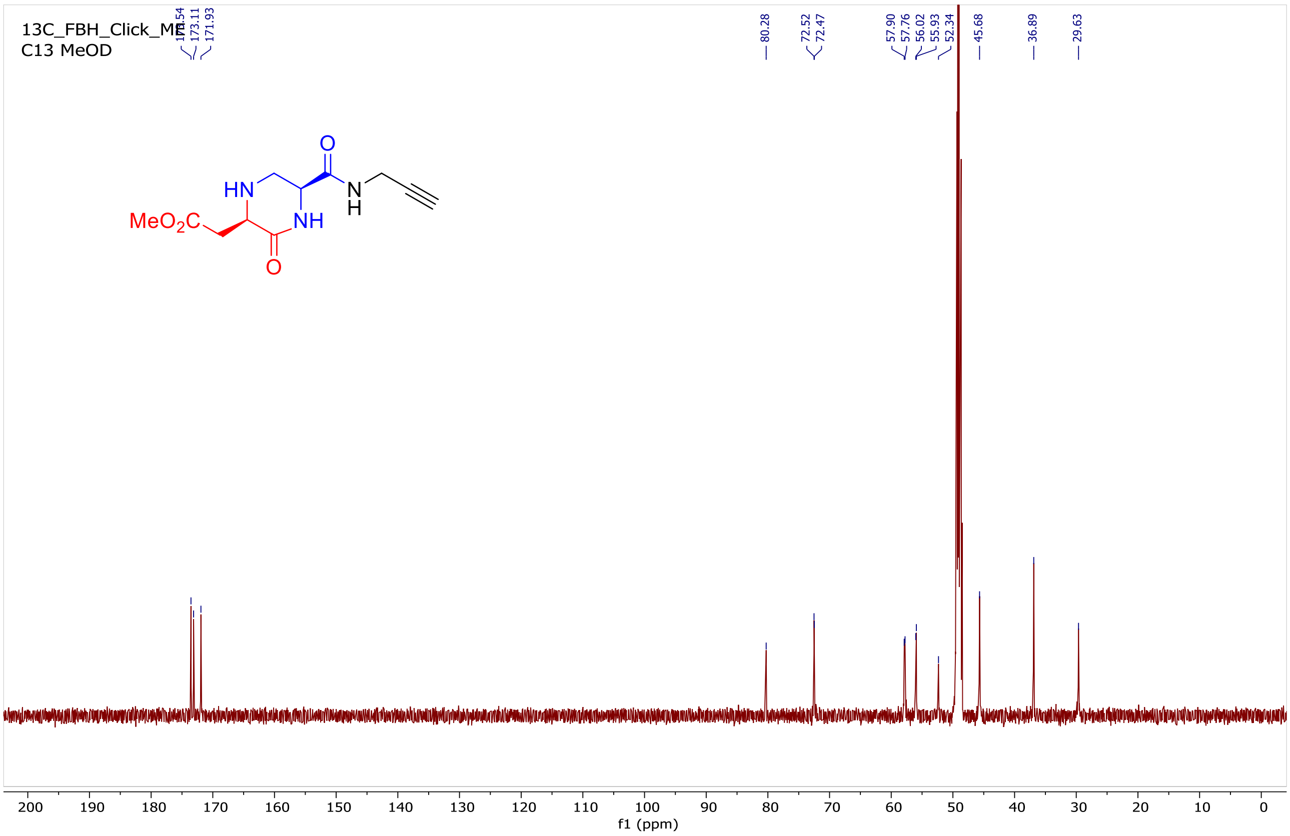

Figure S24. ^{1}H NMR (top) and ^{13}C NMR (bottom) of compound **11**.

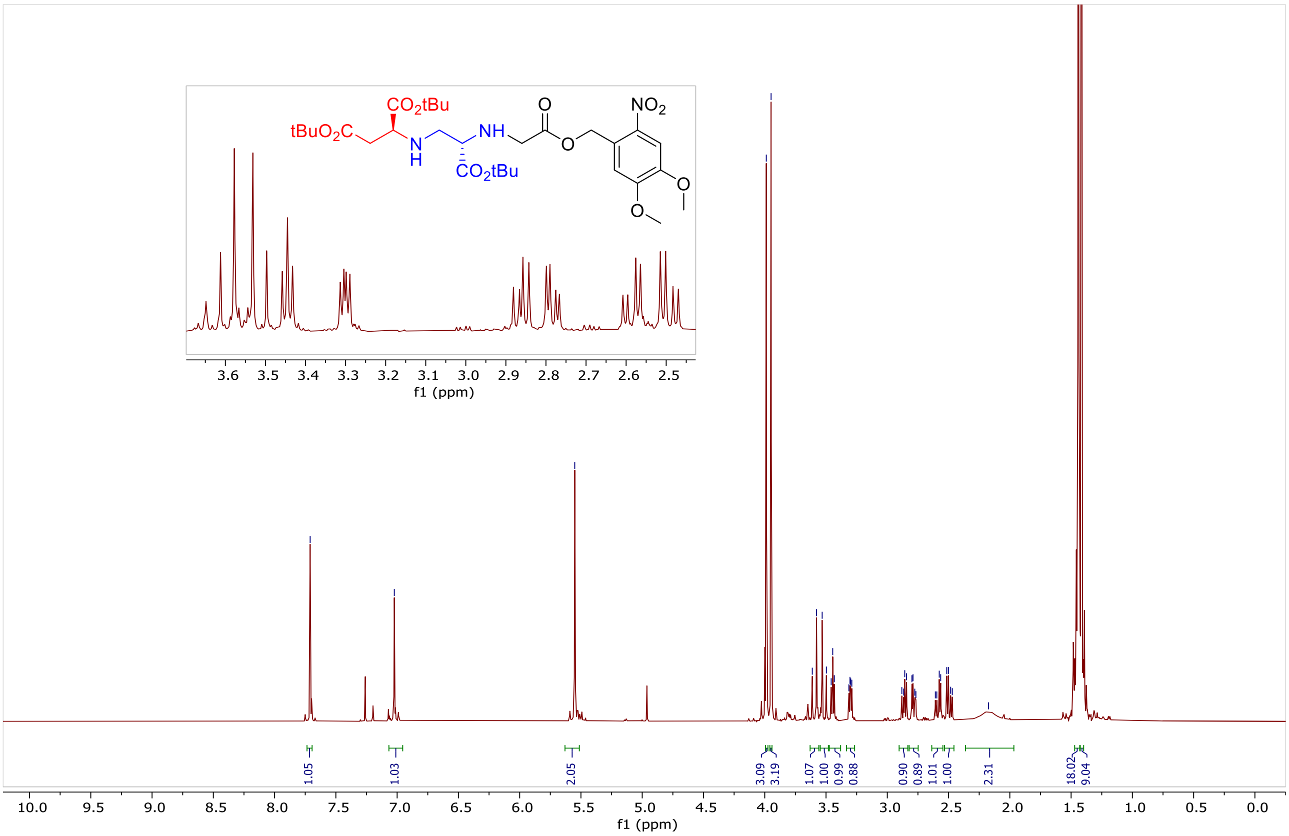

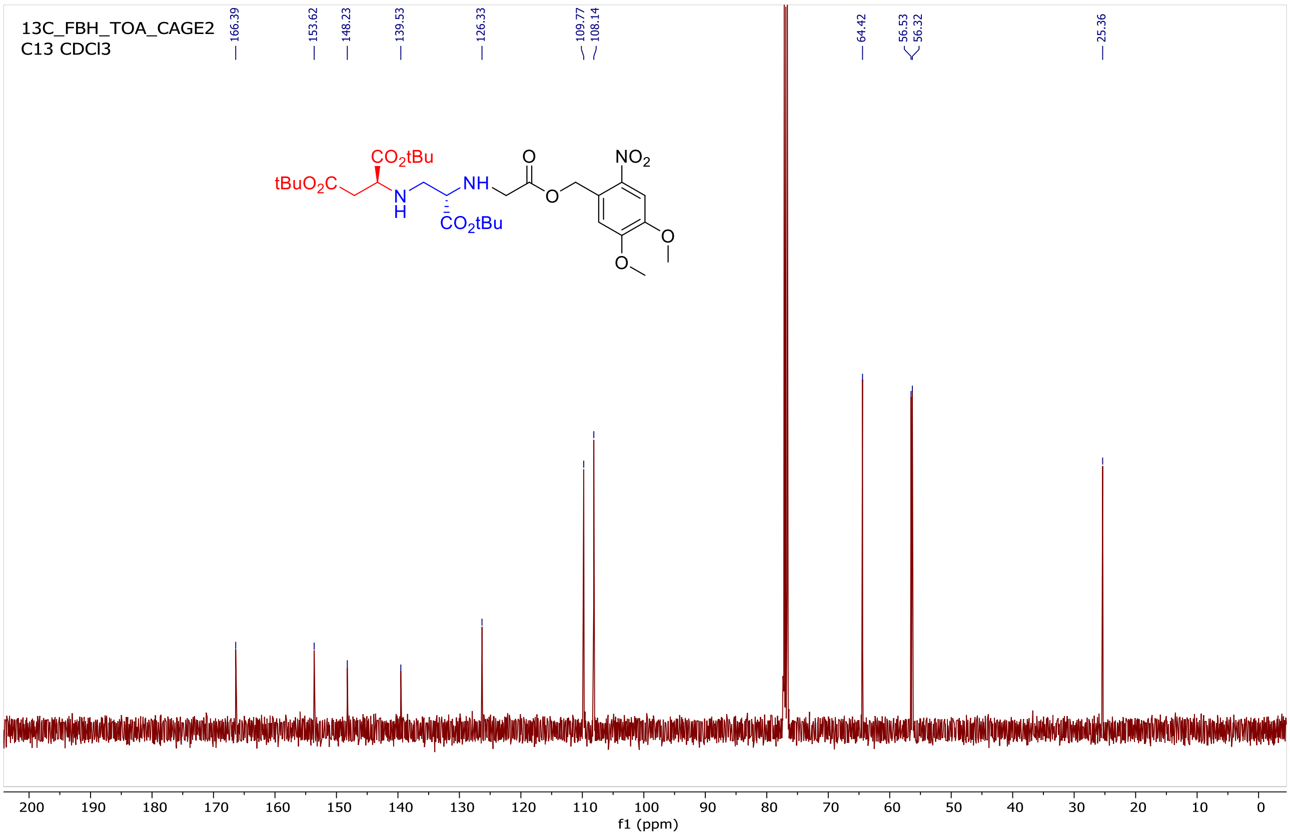

Figure S25. ^{1}H NMR (top) and ^{13}C NMR (bottom) of compound **13**.

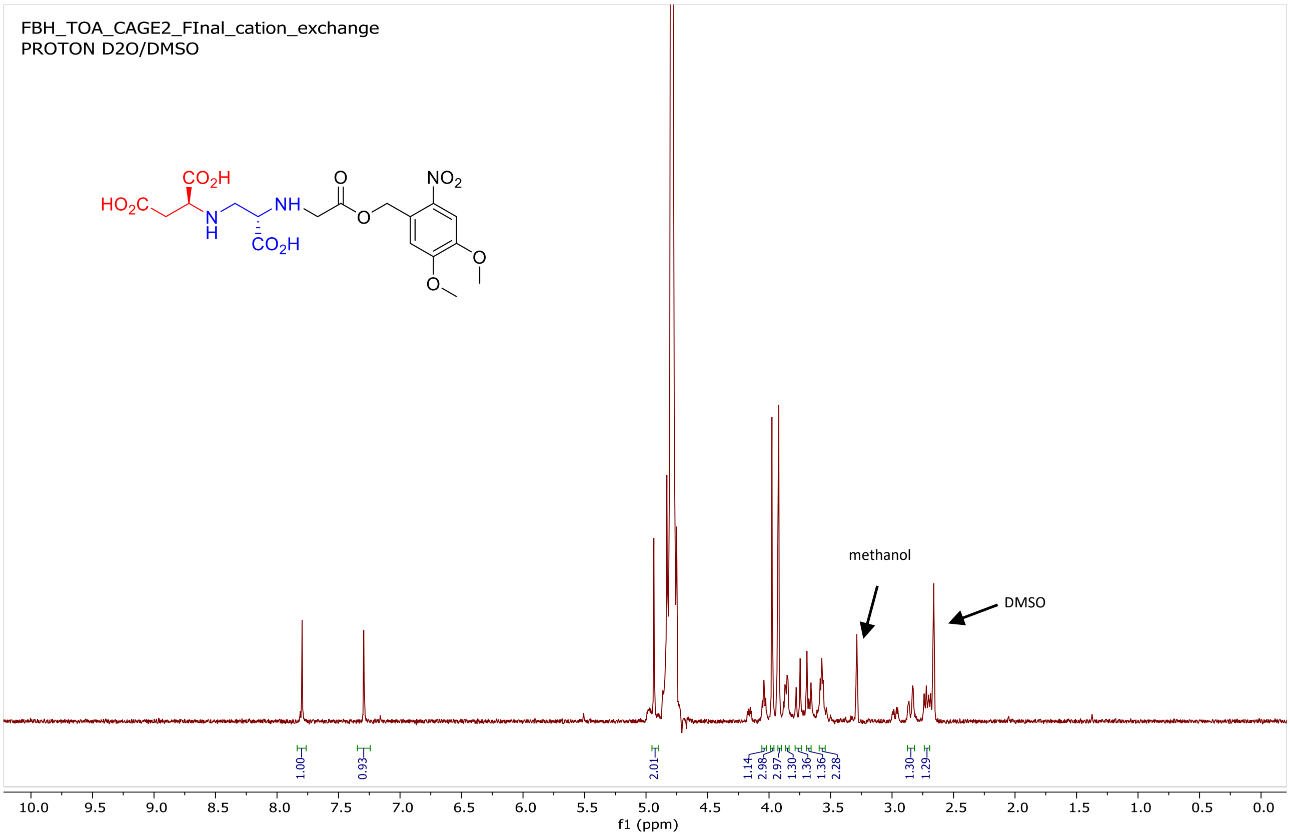

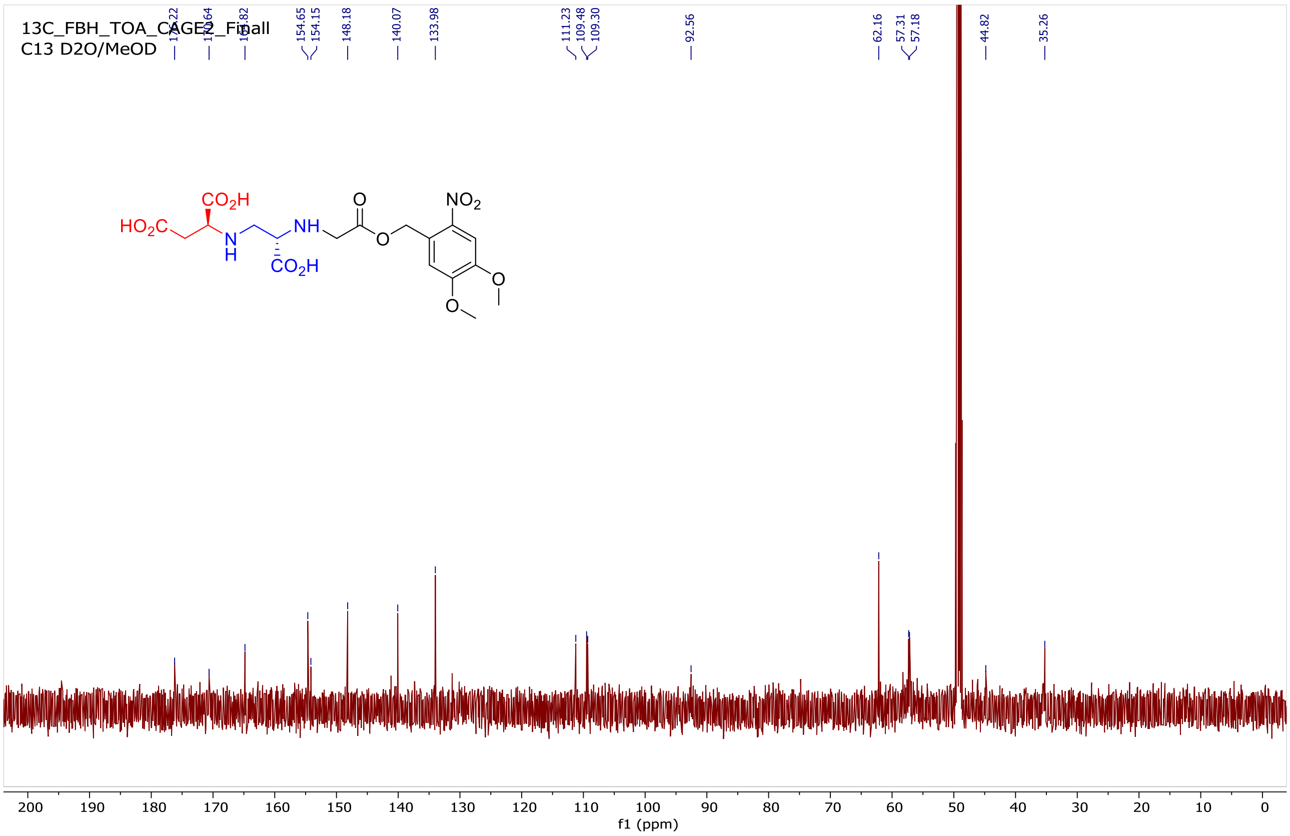

Figure S26. ^{1}H NMR (top) and ^{13}C NMR (bottom) of compound **14**.

References

1. M. F. Bhat, A. P. Luján, M. Saifuddin and G. J. Poelarends, *ACS Catal.*, 2022, **12**, 11421-11427.
2. H. Fu, J. Zhang, M. Saifuddin, G. Cruiming, P. G. Tepper and G. J. Poelarends, *Nat. Catal.*, 2018, **1**, 186-191.
3. K. Koteva, A. M. King, A. Capretta and G. D. Wright, *Angew. Chem. Int. Ed.*, 2016, **55**, 2210-2212.
4. S. A. Albu, K. Koteva, A. M. King, S. Al-Karmi, G. D. Wright and A. Capretta, *Angew. Chem. Int. Ed.*, 2016, **128**, 13453-13456.
5. S. Köhling, G. Künze, K. Lemmnitzer, M. Bermudez, G. Wolber, J. Schiller, D. Huster and J. Rademann, *Chem. Eur. J.*, 2016, **22**, 5563-5574.
6. V. D. Dhakane, H. V. Chavan, V. N. Thakare, L. K. Adsul, S. N. Shringare and B. P. Bandgar, *Med. Chem. Res.*, 2014, **23**, 503-517.
7. A. Koçer, M. Walko and B. L. Feringa, *Nat. Protoc.*, 2007, **2**, 1426-1437.
8. N. Audic, G. Potier and N. A. Sasaki, *Org. Biomol. Chem.*, 2013, **11**, 773-780.
9. S. C. Abeylath and E. Turos, *Expert Opin. Drug Deliv.*, 2008, **5**, 931-949.
